# Isolation of Monoterpene Dihydrochalcones from *Piper montealegreanum* Yuncker (Piperaceae)

**DOI:** 10.3390/molecules22060874

**Published:** 2017-06-09

**Authors:** Harley da Silva Alves, Wilma Raianny Vieira da Rocha, Raimundo Braz-Filho, Maria Célia de Oliveira Chaves

**Affiliations:** 1 Department of Pharmacy/Post-Graduation Program in Pharmaceutical Sciences, State University of Paraíba, 58429-500 Campina Grande-PB, Brazil; wilmaraianny@hotmail.com; 2 Chemical Science Laboratory, CCT, State University of Fluminense North Darcy Ribeiro, 28013-600 Campo dos Goytacazes-RJ, Brazil; braz@uenf.br; 3 Department of Chemistry, DEQUIM-ICE-Federal University Rural of Rio de Janeiro, CEP 23894-374 Seropédica-RJ, Brazil; 4 Research Institute for Drugs and Medicines, Federal University of Paraíba, CEP 58051-900 João Pessoa-PB, Brazil; mariacelia_chaves@yahoo.com.br

**Keywords:** *Piper*, monoterpene dihydrochalcones, *Piper montealegreanum*

## Abstract

Four new compounds were isolated from the branches of *Piper montealegreanum* Yuncker, a shrub found in the Amazon rainforest, including two new dihydrochalcones named claricine (**1**) and maisine (**2**), a cinnamic acid derivative **3** and a phenylalkanoid **4**, along with a porphyrin identified as the known compound phaeophytin a (**5**). The structures were established using spectroscopic experiments, including 1D and 2D NMR and HRESIMS experiments, performed on the two monoterpene dihydrochalcones and their monoacetyl derivatives. The structural diversity of these substances is very important for the *Piper* genus chemotaxonomy.

## 1. Introduction

*Piper* (Piperaceae) is a diverse pantropical plant genus, consisting of approximately 2000 species, and is a rich source of numerous biologically active products [1,2]. Several species of *Piper* are used in traditional medicine as analgesics, anti-inflammatory compounds, and for the treatment of snakebites [3]. The phytochemical investigation of the genus *Piper*, widely distributed in the tropical and subtropical regions of the world, has allowed the identification of many classes of compounds such as aristolactams [4,5], alkamides [6,7], lignans [8], chalcones, and dihydrochalcones [9]. Recently we reported the isolation of some bioactive agents including (*S*)-8-formyl-3′,5-dihydroxy-7-methoxy-6-methylflavanone (**A**), 3′-formyl-3,4′,6′-trihydroxy-2′-methoxy-5′-methylchalcone (**B**) and the phenylpropanoid ethyl-3,4-methylenedioxy-5-methoxy-7,8-dihydrocinnamate (**C**) from a crude ethanolic extract (CEE) of the branches of *Piper montealegreanum* [10], a native species of the Amazon region, north of Brazil [11]. We also recently disclosed the analgesic and anti-inflammatory effects of compounds **A** and **B** [12].

*Piper montealegreanum* is a branching shrub upper with elongate internodes and oblong or lance elliptic leaves with an acuminate apex and subequilaterally rounded. It presents spikes of 3 mm thick and up to 9 cm long; drupes are oblong or obovoid, laterally compressed. It was first recorded in 1908 in the city of Monte Alegre, Pará-Brazil [11].

In the present paper, we report the isolation and structure elucidation of two new compounds **1** and **2**, which we have given the trivial names claricine and maisine, respectively, ethyl-3,4,5-trimethoxy-7,8-dihydrocinnamate (**3**), (5-(3*E*,5*E*)-undeca-3,5-dienyl)benzo[*d*][1,3]dioxole (**4**) and the known compound phaeophytin a (**5**). The structures of the compounds were determined by interpretation of the spectral data, including 1D and 2D (HMQC, HMBC, COSY and NOESY) NMR and HRESIMS experiments, of the new monoterpene dihydrochalcones and their monoacetyl derivatives in conjunction with data reported in the literature.

## 2. Results and Discussion

**Scheme 1:**
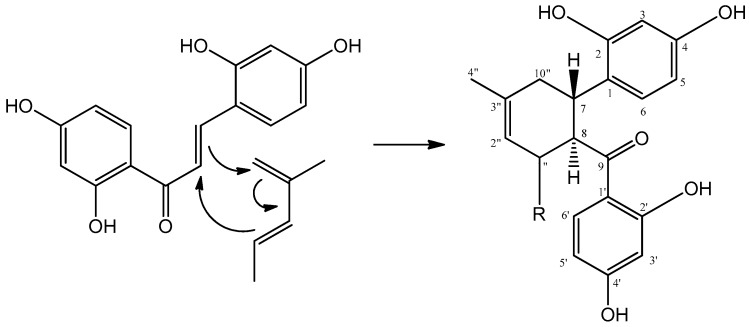
Probable biosynthetic route for monoterpene dihydrochalcones.

**Figure 1:**
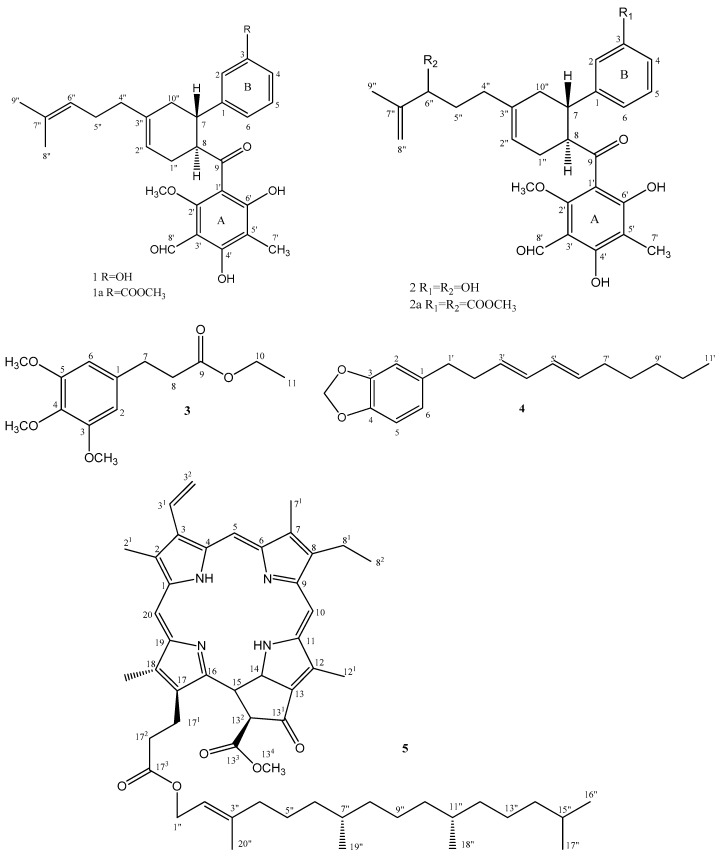
Compounds isolated from *P. montealegreanum*.

Chromatographic procedures led to the isolation of five compounds from *P. montealegreanum* (Figure 1). Compounds **1** and **2** were identified as monoterpene dihydrochalcones. One of the most characteristic features of the structural chemistry of chalcones and dihydrochalcones is their ability to act as dienophiles in enzyme-catalyzed Diels-Alder reactions [13]. In the biosynthetic route proposed by Hano et al. [14] it was possible predicted how an initial adduct reacted with the 2′,4′,2,4-tetrahydroxychalcone to form the monoterpene dihydrochalcone (Scheme 1). Few plant families such as the Anonnaceae [15], Moraceae [16] and Piperaceae [17] have a group of adducts. The crinatusins, obtained from the stems of *Cyathocalyx crinatus*, showed lethal toxicity in a brine shrimp bioassay [15]. Palodesangrens A–E, obtained from the bark of *Brosimum rubescens*, effectively inhibited the formation of a 5a-dihydrotestosterone androgen receptor complex implicated in androgen-dependent diseases [18]. Cytotoxicity toward KB cells was recorded for both fissistin and isofissistin [19].

The general metabolism of phenylpropanoid **3** can generate an enormous array of secondary metabolites (flavonoids, coumarins, lignins, hydrolyzable tannins) based on the few intermediates of the shikimate pathway as the core unit [20]. Phenylalkanoids like **4** act as chemotaxonomy markers and exhibit antifungal activity [21]. The porphyrinic compounds (such as phaeophytin a) are derived from chlorophyll a and are widely present in the plant kingdom [22].

The various compounds isolated from different *Piper* species have been classified under twelve categories, viz. alkaloids/amides, propenylphenols, lignans, neolignans, terpenes, steroids, kawapyrones, piperolides, chalcones and dihydrochalcones, flavones, flavanones and miscellaneous compounds [4]. Most of the chalcones and dihydrochalcones isolated from the *Piper* species are oxygenated at the C-2′, C-4′ and C-6′ positions and the B-ring is unsubstituted in all the chalcones and dihydrochalcones, except asebogenin [23], flavokawains A and C [24] in which C-4 position is also oxygenated. Five new unusual monoterpene-substituted dihydrochalcones, adunctins A–E [25], were isolated from the leaves of *P. aduncum.* Various propenylphenols have been isolated so far from different *Piper* species, such as ω-hydroxyisodillapiole [26] and safrole [27]. Besides the above mentioned groups of compounds, several long chain alkanes, alcohols, acids, esters and oxygenated cyclohexanes have been isolated from *Piper* species (e.g., villiramulin B) [28].

Compound **1** was obtained as yellow gummy solid and its IR spectrum showed absorption bands at ν_max_ 3433 cm^−1^ (OH), 2850 cm^−1^ (formyl C–H), 1618 cm^−1^ (suggesting conjugated carbonyl), and 1458 and 1419 cm^−1^ (aromatic ring). The molecular formula C_28_H_32_O_6_ was determined by negative-mode HRESIMS measurement of the molecular ion at *m*/*z* 463.2150 ([M − H]^−^, calcd. for C_28_H_31_O_6_, 463.2126, delta 5.2 ppm), corresponding to 13 double-bond equivalents.

The UV spectrum (in MeOH) showed two absorption bands at λ_max_ 274 and 338 (sh) nm. The use of AlCl_3_ produced bathochromic shift to λ_max_ 299, 313 and 371 (sh) nm, respectively, suggesting the presence of a flavonoid-sustaining chelated hydroxyl group and/or *ortho*-dihydroxy system [13,29].

The ^13^C-NMR spectrum revealed carbon signals (Table 1) corresponding to the presence of four methyl (including methoxyl group at δ_C_ 67.23), four sp^3^ methylene (δ_C_ 29.79, 26.38, 36.88 and 37.23), nine methine (two sp^3^ (δ_C_ 45.19 and 51.0) and seven sp^2^ (including one formyl group at δ_C_ 192.36), and eleven sp^2^ non-hydrogenated (including one conjugated carbonyl at δ_C_ 209.55) carbon atoms, compatible with partial expanded molecular formula (C=O)(C)_10_(CHO)(CH)_8_(CH_2_)_4_(CH_3_)_3_(CH_3_O) = C_28_H_29_O_3_, suggesting the presence of three hydroxyl groups consistent with the ^13^C chemical shifts observed for oxygenated aromatic carbon atoms at δ_C_ 155.46 (C-3), 165.21 (C-4′, *para*-conjugated with carbonyl) and 167.23 (C-6′, *ortho*-conjugated with carbonyl), along with one methoxylated C-2′ at δ_C_ 166.65 (Table 1).

**Scheme 2:**
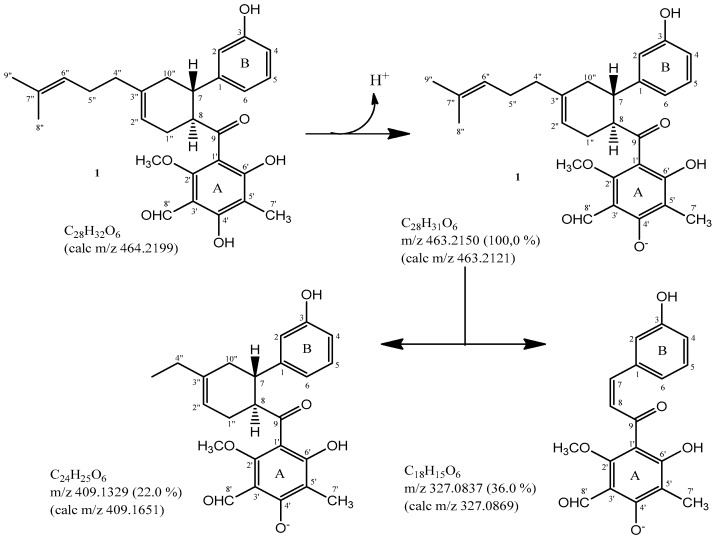
Proposed fragmentation mechanisms (only peaks classified as principals) in the HRESITOFMS of **1**.

This data, involving a comparison with ^1^H- and ^13^C-NMR data of hostmanin D [30], suggested **1** to be a prenylated dihydrochalcone with a totally substituted ring A. This deduction was supported by differences justified by the presence of formyl and hydroxyl groups at rings A and B of **1**, in agreement with HRESITOFMS (negative mode) compatible with the proposed fragmentation summarized in Scheme 2.

**Figure 2:**
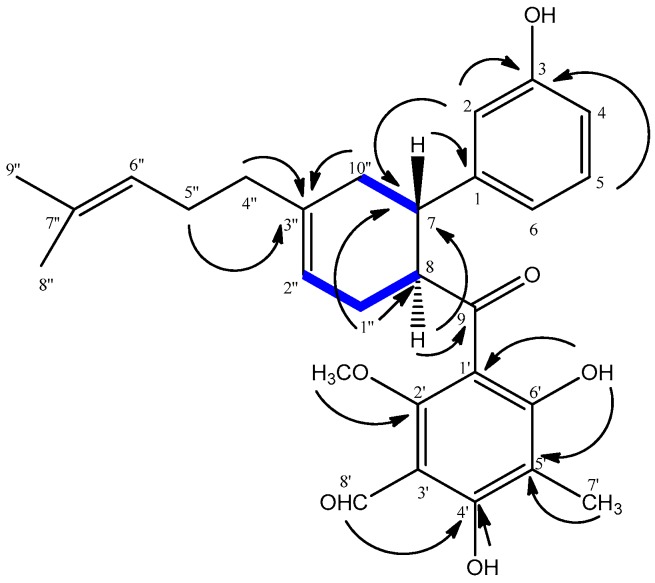
The 2D NMR correlations of compound **1**: ^1^H-^1^H correlation spectroscopy (^1^H-^1^H COSY) (bold) and selected heteronuclear multiple bond correlations (HMBC) (arrows).

The ^1^H-NMR and HMQC spectra of the compound **1** (Table 1) exhibited signals attributed to dimethylallyl groups CH_3_-8″ (δ_H_ 1.62 (d, *J* = 0.5 Hz)/δ_C_ 17.84, shielded by γ-effect of the methylene group CH_2_-5″) and CH_3_-9″ (δ_H_ 1.70 (d, *J* = 1.0 Hz, 3H)/δ_C_ 25.69), the aromatic methoxy group MeO-2′ (δ_H_ 3.90 (s)/δ_C_ 67.23, located in a position steric hindrance), the aryl formyl hydrogen 4-CHO (δ_H_ 10.04 (s)/δ_C_ 192.36), the aromatic methyl group CH_3_-7′ (δ_H_ 1.89 (s)/δ_C_ 6.56), two olefinic signals CH-2″ (δ_H_ 5.53 (m)/δ_C_ 118.73) and CH-6″ (δ_H_ 5.12 (qt, *J* = 7.0, 1.5 Hz)/δ_C_ 124.02), two hydrogen-bonded OH at δ_H_ 12.88 (1H, s, OH-6′) and 12.46 (1H, s, OH-4′), and a disubstituted phenyl group. Concerning this phenyl group, the NMR and HMQC spectra displayed four methine carbons: CH-2 at δ_H_ 6.58 (dd, *J* = 2.5, 1.5 Hz)/δ_C_ 114.48, CH-4 at δ_H_ 6.48 (ddd, *J* = 7.5, 2.5, 1.5 Hz)/δ_C_ 113.30, CH-5 at δ_H_ 6.94 (t, *J* = 7.5 Hz)/δ_C_ 129.35, and CH-6 at δ_H_ 6.64 (dd, *J* = 7.5, 1.5 Hz)/δ_C_ 119.82. These data suggested a 1,3-disubstituted aromatic ring. The HMBC spectrum showed heteronuclear correlations of the signals corresponding to non-hydrogenated carbon C-5′ at δ_C_ 108.85 with hydrogens 3H-7′ at δ_H_ 1.89 (^2^*J*_CH_), HO-4′ at δ_H_ 12.46 (^3^*J*_CH_) and HO-6′ at δ_H_ 12.88 (^3^*J*_CH_). Thus, these data were used to locate the methyl group (δ_H_ 1.89) at C-5′ and the hydroxyl groups (δ_H_ 12.88 and 12.46) in C-6′ and C-4′, respectively. The correlations between the signal at δ_C_ 107.97 (C-3′) with the chelated hydroxyl group at δ_H_ 12.46 (HO-4′, ^3^*J*_CH_) and the formyl hydrogen at δ_H_ 10.04 (CHO-8′, ^3^*J*_CH_) were used to assign the aldeidic group to C-3′ and to confirm the chelated hydroxyl group at δ_H_ 12.46 to C-4′. Correlations observed in the HMBC spectrum between δ_H_ 3.90/δ_c_ 166.65 permitted us to assign the methoxy group at δ_H_ 3.90 in C-2′. The presence of signals corresponding to two methine carbons α (δ_C_ 51.06, CH-8) and β (δ_C_ 45.19, CH-7) of the dihydrochalcone skeleton suggested that a diprenylated moiety was located in these positions. This deduction is confirmed by the correlations revealed by the HMBC spectrum: δ_C_ 209.55 (C-9), 45.19 (CH-7), 29.79 (CH_2_-1″) and 36.88 (CH_2_-10″) with δ_H_ 4.13 (H-8) (Figure 2). Additional heteronuclear long-range couplings are summarized in Table 1.

**Figure 3:**
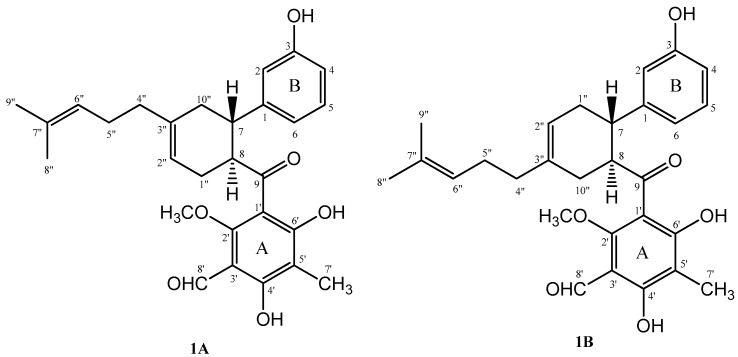
Structural alternatives preliminarily proposed for compound **1**.

The relative stereochemistry at CH-7 and CH-8 was deduced from the *J*-value between H-7 and H-8 (*J* = 11.0 Hz) characteristic of a *trans* axial-axial coupling [30]. The HMBC correlations observed between H-4″ (δ_H_ 2.02 (m)), H-10″ (δ_H_ 2.23 (m) and 2.20 (m)) and H-5″ (δ_H_ 2.14 (m)) with C-3″ (δ_C_ 137.41) confirmed the attachment of the prenyl unit at C-3″ (Table 1). However, there was two structural alternatives for the diprenylated moiety. The COSY spectrum visualized correlations between δ_HH_ 2H-10″/H-7 and δ_HH_ 2H-1″/H-8 and established the first structural alternative [15,30] (Figure 3).

In the ^13^C-NMR spectrum of the acetylated derivative (1a) of **1**, a magnetic deshield was observed at CH-2 and CH-4 (*ortho* positions) and at CH-6 (*para* position) of δ_C_ 5.7, 6.1 and 5.2 ppm, respectively, in accordance with the acetylation of OH-3 located in the B aromatic ring and confirming that this type of monosubstituition is relatively rare in the B ring of flavonoids. In addition, the presence of the acetate group was confirmed from absorption of methyl carbon at δ_C_ 20.86 and of the ester carbonyl at δ_C_ 169.05.

**Scheme 3:**
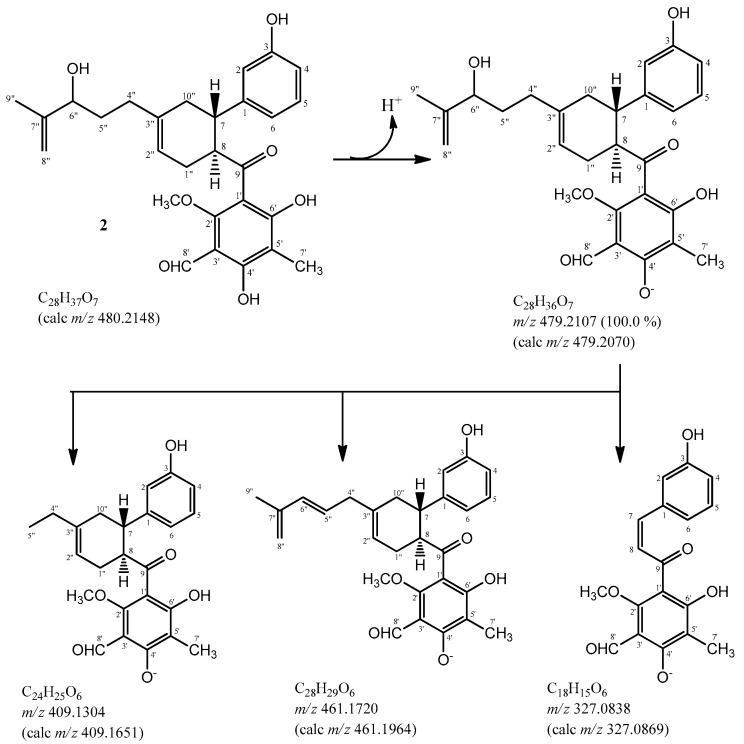
Fragments proposed to justify the main peaks observed in the HRESITOFMS of **2**.

Compound **2** was isolated as yellow gum with molecular formula C_28_H_32_O_7_ determined by ESI-Q-TOF-HRMS at *m*/*z* 479.2107 ([M − H]^−^, calcd. 480.2149) (Scheme 3).

The IR spectrum showed a broad band at ν_max_ 3429 cm^−1^ due to hydrogen bonded OH, conjugated formyl carbonyl (1716 cm^−1^), conjugated cetonic carbonyl (ν_max_ 1620 cm^−1^) and aromatic ring (ν_max_ 1454 and 1419 cm^−1^). In its UV spectrum in MeOH, the presence of an absorption band was observed at λ_max_ 274 nm plus a shoulder at 338 nm. The use of a shift reagent (AlCl_3_) promoted a bathochromic shifts λ_max_ 312 nm and 368 nm, suggesting the presence of one dihydrochalcone [12,29]. In the ^1^H-NMR spectrum of **2**, characteristic signals of a 2′-methoxy-3′-formyl-4′,6′-dihydroxy-5′-(methyl-phenyl)-7-phenyl-(3-hydroxy)-cyclohex-2″-enyldihydrochalcone derivative were observed (Table 1). These spectroscopic data are quite similar to those of **1**. The ^13^C-NMR spectrum contained six additional resonances with one quaternary carbon (δ_C_ 147.33, C-7″), one methyl at δ_C_ 17.63 (CH_3_-9″), three methylenes at δ_C_ 33.20 (CH_2_-4″), 32.91 (CH_2_-5″) and 111.19 (CH_2_-8″), and one methine at δ_C_ 75.73 (CH-6″) bearing an O-atom. The presence of an olefinic methylene signal (δ_C_ 111.19) and only a methyl signal (δ_C_ 17.63), associated with molecular formula (C_28_H_32_O_7_), suggested that **2** has a hydroxyl group at C-6″ and a double bond at C-7″, in contrast to **1** that has one quaternary carbon (δ_C_ 131.70) and two methyl (δ_C_ 17.84 and 25.69), two methylenes (δ_C_ 37.23 and 26.38) and one methine (δ_C_ 124.02) signals. The spectroscopic data of 6″-hydroxy-7″-methylpentenyl moiety was established with combined results of HMQC and HMBC experiments and was supported by HMBC correlations between CH-6″ (δ_C_ 75.73) and 2H-8″ (δ_H_ 4.93 (m), δ_H_ 4.84 (m) and Me-9″ (δ_H_ 1.73 (m)).

Spectral analyses of 2a showed a magnetic deshielding effect at CH-2, CH-4 and CH-6 for δ_C_ 120.30, 119.35 and 124.97, respectively. The absorptions at δ_C_ 170.30 (C-10) and 21.08 (CH_3_-11) confirmed the presence of the acetate group. A marked difference was also observed in the chemical shift at CH-6″ for δ_C_ 71.17, suggesting the presence of the acetate group at C-6′ and a shield of C-7″ and CH_2_-5″ by γ-effect of the carbonyl carbon.

The phenylpropanoid was obtained of a mixture with two substances: the majority being **1** and other encoded as **3** (Figure 3). In the ^13^C-NMR spectrum, the presence of 39 signals was observed (APT experiment), 11 assigned to **3**, including four for quaternary carbons (δ_C_: 136.00 (C-1), 153.21 (C-3/5), 136.50 (C-4) and 173.80 (C-9)), one for methine carbon (δ_C_ 105.36 (CH-2/CH-6)), three for methylenes carbons (δ_C_: 31.37 (CH_2_-7), 36.09 (CH_2_-8) and 60.47 (CH_2_-10)) and three methyl carbons (δ_C_ 60.82 (MeO-4), 56.09 (MeO-3/5), and 14.22 (CH_3_-11)). In the ^1^H-NMR spectrum, three singlets were seen at δ_H_: 6.40 (2H, brs, H-2/H-6), 3.82 (6H, s, MeO-3/MeO-5) and 3.80 (3H, s, MeO-4), the last two being characteristic of aromatic methoxyls, suggesting the presence of a tetrasubstituted aromatic ring in the structure. Other signals observed were δ_H_: 2.87 (t, *J* = 8.0 Hz, 2H-7), 2.59 (t, *J* = 8.0 Hz, 2H-8), 4.10 (m, 2H-10) and 1.22 (t, *J* = 7.0 Hz, 3H-11). The NMR assignments and comparison with literature data [31] allowed us to deduce **3** was a new cinnamic acid derivative, 3,4,5-trimethoxy-hydrocinnamic acid ethyl ester.

Compound **4** was isolated as a yellow oil. The IR spectrum showed bands at ν_max_ 2924 and 2852 cm^−1^ characteristic of aliphatic C–H stretching; at ν_max_ 1489 and 810 cm^−1^ consistent with C=C trisubstituted aromatic ring; and at ν_max_ 1442 and 1246 cm^−1^ suggestive of C–H and C–O stretch, respectively. Broad bands at ν_max_ 1718, 1653 and 939 cm^−1^ suggested the presence of bond-conjugated C=C with *trans* stereochemistry [32].

The ^1^H-NMR (δ, CDCl_3_, 500 MHz) showed three signals at δ_H_: 6.70 (d, *J* = 8.0 Hz, H-5), 6.65 (brd, *J* = 1.5 Hz, H-2) and 6.60 (dd, *J* = 8.0, 1.5 Hz, H-6) and a singlet at δ_H_ 5.89 (2H, OCH_2_O-3-4), characteristic of methylenedioxy group. These data suggested the presence of trisubstituted aromatic ring. The signals at δ_H_: 5.99 (brt, *J* = 14.5, 1.0 Hz, H-4′/H-5′), 5.58–5.52 (m, H-4′/H-6′), 2.59 (t, *J* = 7.5 Hz, 2H-1′), 2.30 (td, *J* = 7.5, 8.0 Hz, 2H-2′), 2.02 (td, *J* = 7.5, 7.0 Hz, 2H-7′), 1.35–1.31 (m, 2H-8′), 1.29–1.26 (m, 2H-9′, 2H-10′) and 0.86 (t, *J* = 7.0 Hz, 3H-11′) suggested the presence of an unsaturated acyclic chain with a terminal aliphatic methyl. In the ^13^C-NMR APT experiment, 18 signals were observed , three of which were for quaternary carbons at δ_C_ 147.47 (C-3), 145.54 (C-4) and 135.80 (C-1); seven for methine carbons at δ_C_ 133.10 (CH-6′), 130.99 (CH-4′), 130.84 (CH-3′), 130.08 (CH-5′), 121.08 (CH-6), 108.85 (CH-2), 108.05 (CH-5); seven for methylene carbons at δ_c_ 100.69 (OCH_2_O), 35.62 (CH_2_-1′), 34.70 (CH_2_-2′), 32.59 (CH_2_-7′), 31.89 (CH_2_-9′), 29.40 (CH_2_-8′) and 22.67 (CH_2_-10′); and one methyl carbon at δ_c_ 14.12 (Me-11′) [22]. The structure of **4** was supported by 2D NMR experiments (Table 2) and literature data [28] and elucidated as (5-(3*E*,5*E*)-undeca-3,5-dienyl)benzo[*d*][1,3]dioxole (Figure 3). Using similar methods as above along with literature data [22,33], compound **5** was identified as phaeophytin a (Figure 3), isolated for the first time from *P. montealegreanum.*

## 3. Materials and Methods

### 3.1. General Procedures

UV spectra were recorded on a Vankel-50 UV-Vis spectrophotometer (Varian, Palo Alto, CA, USA) and AlCl_3_ was used as shift reagent. IR spectra were obtained on an FT-IR-1750 spectrophotometer (Perkin-Elmer, Waltham, MA, USA) using KBr disks. The spectra mass were obtained on a LCMS-IT-TOF (225-07100-34) (Shimadzu, Tokay, Japan) equipped with a Z-spray ESI (electrospray) source and operated in negative mode. ^1^H- and ^13^C-NMR (1D and 2D) spectra were recorded on a Mercury 500 and 200 MHz spectrometer (Varian, Palo Alto, CA, USA) in CDCl_3_ using TMS as internal standard. Sephadex LH-20 and silica gel 60 (PF 254 art. 7749 and art. 7731) were purchased from Merck (Kenilworth, NJ, USA). Hexane, chloroform, ethyl acetate and methanol were of analytical grade and were purchased from Merck. The acetylated flavonoids were obtained by dissolving the samples in pyridine and acetic anhydride (1:1—*v*/*v*), and the mixture was maintained under stirring for 48 h. The reaction progress was monitored by TLC. After this period, the mixture was treated with ice-cold distilled water until the formation of a precipitate. The precipitate was filtered and washed several times with distilled water, dissolved in chloroform, and dried with anhydrous sodium sulfate. The remaining solvent was evaporated in a rotating evaporator [34].

### 3.2. Plant Material

Branches from *P. montealegreanum* Yuncker were collected in Belém (Pará State, Brazil; Latitude 14°10′00″ S, longitude 53°05′00″ W). The botanical material was identified by Dra. Elsie F. Guimarães, at the Botanical Department of UFRJ, and a specimen voucher was deposited at Emilio Goeldi Museum, Belém, under serial number MSP-010.

### 3.3. Extraction and Isolation

A powered material of *P. montealegreanum* (1.3 kg) was treated with EtOH (4 × 2.0 L) at room temperature (30 °C) and for three consecutive days, then the solvent was removed under reduced pressure to furnish the crude ethanolic extract (CEE, 115 g), which was partitioned in a series of solvents to yield hexane (44 g), chloroform (37 g), ethyl acetate (8 g) and hydroalcoholic (4.5 g) fractions. A portion (9.5 g) of the hexane fraction was chromatographed over a silica gel column using a gradient of solvents with increasing polarity (hexane, chloroform and methanol) yielding 109 fractions. The fractions 38–42 (1.169 mg) were rechromatographed using Sephadex LH-20 and eluted with methanol. Of the 19 fractions obtained, fractions 12–13 and 14 were purified over silica gel column and preparative TLC to yield **1** (9 mg) and **2** (6 mg), respectively. Fraction 10–11 was chromatographed on Sephadex LH-20 and purified by preparative TLC yielding **4** (12 mg). The supernatant of 12–13 was chromatographed by preparative TLC to obtain a mixture of **1** and **3** (14 mg). 11.0 g of chloroform phase, eluted over Sephadex LH-20 column with a binary mixture of methanol−chloroform (1:1—*v*/*v*), provided compound **5** (25 mg).

(*2′-Methoxy-3′-formyl-4′,6′-dihydroxy-5′-methylphenyl)-[3″-(7″-dimethylbut-6″-enyl)-7-phenyl-(3-hydroxy)-cyclohex-2″-enyl]-methyl-9-one* (claricine, **1**). Yellow gum; UV (MeOH) λ_max_ 274, 338 (sh) nm; (MeOH + AlCl_3_) λmax 299, 313, 371 (sh) nm; IR (KBr disc) ν_max_ 3.433, 2.922, 1.618, 1.458, 1.419 cm^−1^; for ^1^H-NMR and ^13^C-NMR spectroscopic data, see Table 1; HRESIMS *m*/*z* 463, 2150 ([M − H]^−^, (calcd. for C_28_H_32_O_6_, 464,2200).

*Acetylated monoterpene dihydrochalcone* 1a. For ^1^H-NMR ^1^H-NMR and ^13^C-NMR spectroscopic data, see Table 1.

(*2′-Methoxy-3′-formyl-4′,6′-dihydroxy-5′-methylphenyl)-[3″-(6′-hydroxy-7″-methylpentenyl)-7-phenyl-(3-hydroxy)-cyclohex-2″-enyl]-methyl-9-one* (maisine, **2**). Yellow gum; UV (MeOH) λ_max_ 274, 338 (sh) nm; (MeOH + AlCl_3_) λ_max_ 300, 312, 368 (sh) nm; IR (KBr disc) ν_max_ 3429, 2924, 1716, 1620, 1454, 1419 cm^−1^; for ^1^H-NMR and ^13^C-NMR spectroscopic data, see Table 1; HRESIMS *m*/*z* 479,2107 [M − H]^−^ (calcd. for C_28_H_32_O_7_, 480,2149).

*Acetylated monoterpene dihydrochalcone* 2a. For ^1^H-NMR and ^13^C-NMR spectroscopic data, see Table 1.

*Ethyl-3,4,5-trimethoxy-7,8-dihydrocinnamate* (**3**). ^1^H-NMR (CDCl_3_, 500 MHz) δ_H_ 6.40 (2H, s), 4.10 (2H, m), 3.82 (6H, s, OCH_3_-3 and 5), 3.80 (3H, s, OCH_3_-4), 2.87 (2H, t, *J* = 8.0 Hz, H-7), 2.59 (2H, *J* = 8.0 Hz, H-8), 1.22 (3H, t, *J* = 7.0 Hz); ^13^C-NMR (CDCl_3_) δ_C_ 14.22, 31.37, 36.09, 56.09 (2C), 60.47, 60.82, 105.36, 136.00, 136.50, 153.21, 173.80.

*(5-(3E,5E)-Undeca-3,5-dienyl)benzo[d][1,3]dioxole* (**4**). Yellow oil; IR (KBr disc) ν_max_ 2924, 2852, 1718, 1653, 1489, 1442, 1246, 939, 810 cm^−1^; for ^1^H-NMR and ^13^C-NMR spectroscopic data, see Table 2.

*Phaeophytin a* (**5**). Green powder; IR (KBr disc) ν_max_ 3433, 2924, 2850, 1731, 1701, 1620, and 1377 cm^−1^; ^1^H-NMR and ^13^C-NMR data were consistent with literature [22,33].

## 4. Conclusions

The present study, based on the phytochemical investigations of *P. montealegreanum*, using chromatographic and spectroscopic techniques, led to the identification of five substances, including two flavonoids with rare skeletons, called monoterpene dihydrochalcones. The acetylated derivatives of the dihydrochalcones claricine (**1**) and maisine (**2**) were prepared to identify their structures. The cinnamic acid derivative, ethyl-3,4,5-trimethoxy-7,8-dihydrocinnamate (**3**) and the phenylalkanoid, (5-(3*E*,5*E*)-undeca-3,5-dienyl)benzo[*d*][1,3]dioxole (**4**) were isolated for the first time as natural products, along with the known compound phaeophytin a (**5**). Monoterpene dihydrochalcones are unusual compounds restricted to a few plant genus, such as *Piper*. Besides that, phenylpropanoids are precursors of some classes of secondary metabolites, such as flavonoids. Thus, these structures are important for the chemotaxonomy of the *Piper* genus. These data corroborate with previous studies on the species evaluating the biological properties of the isolated products.

## Figures and Tables

**Table 1:** ^1^H (500 MHz) and ^13^C (125 MHz) NMR for **1**, 1a, **2** and 2a, including results obtained by heteronuclear 2D shift-correlated HMQC (1JCH) and HMBC (*^n^J*_CH_, *n* = 2 and 3), using CDCl_3_ as solvent. Chemical shifts (δ, ppm) and coupling constants (*J*, Hz) are shown in parentheses *.

	1			1a	2			2a
C Type	δ_C_	δ_H_	HMBC	δ_C_	δ_C_	δ_H_	HMBC	δ_C_
C-1	145.56		5, 6, 7	144.27	145.39		5	145.14
CH-2	114.48	6.58, dd (2.5, 1.5)	4, 6	120.24	114.40	6.56, m	6, 7	120.30
C-3	155.46		2, 5	150.63	155.52		5	150.66
CH-4	113.30	6.48, ddd. (7.5, 2.5, 1.5)	2, 6	119.43	113.35	6.47, md	2, 6	119.35
CH-5	129.35	6.94, t (7.5)		128.98	129.27	6.92, dt (7.5, 2.0)		127.82
CH-6	119.82	6.64 dd (7.5, 1.5)	2, 4	125.08	119.79	6.62, md	2, 4, 7	124.97
CH-7	45.19	3.01, dt (11.0, 5.5)	2, 8, 2H-1″	44.84	45.08	3.00, dt (11.0, 5.5)	2, 6	44.79
CH-8	51.06	4.13, dt (11.0, 5.5)	7, 2H-10″	50.93	51.01	4.09, m	7	50.87
C-9	209.55		8	209.21	209.40			209.03
C-10				169.05				170.30
C-1′	109.34		HO-6′	108.95	109.28		HO-6′	109.00
C-2′	166.65		CH_3_O-2′	166.60	166.60		CH_3_O-2′	166.65
C-3′	107.97		8′, HO-4′	108.22	107.98		8′, HO-4′	108.06
C-4′	165.21		8′, HO-4′, 3H-7′	165.34	165.26		HO-4′, 8′, 3H-7′	165.36
C-5′	108.85		3H-7′, HO-4′, HO-6′	108.88	108.89		3H-7′, HO-4′, HO-6′	109.00 119.35
C-6′	167.23		HO-6′, 3H-7′	167.24	167.22		HO-6′, 3H-7′	167.28
CH_2_-1″	29.79	2.40, 2.38	H-7, H-8	29.78	29.71	2.42, m 2.37, m		29.79
CH-2″	118.73	5.53, m		118.69	119.15	5.55, m		119.53
C-3″	137.41		2H-4″, 2H-10″, 2H-5″	137.28	137.17			136.36
CH_2_-4″	37.23	2.02 m	2H-5″	37.17	33.20	2.23-2.18, m		30.60
CH_2_-5″	26.38	2.14 m	2H-4″	26.33	32.91	1.65, m		30.37
CH-6″	124.02	5.12 qt (7.0, 1.5)	2H-5″, 3H-8″, 3H-9″	123.93	75.73	4.08, m	2H-8″, 3H-9″	71.17
C-7″	131.70		H-6″, 3H-8″, 3H-9″	131.63	147.33		3H-9″	145.14
CH_3_-8″	17.84	1.62 d (0.5)	3H-9″	18.00	111.19	4.93, m 4.84, m	6″, 3H-9″	112.98
CH_3_-9″	25.69	1.70 d (1.0)	3H-8″	25.66	17.63	1.73, s	2H-8″	18.07
CH_2_-10″	36.88	2.23, m, 2.20 m	H-7, H-8	36.78	36.67	2.23, m 2.18, m		36.76
OCH_3_-2′	67.23	3.90, s		67.27	67.24	3.90, s		67.33
CH_3_-7′	6.56	1.89, s		6.52	6.57	1.87, s		6.56
CHO-8′	192.36	10.04, s		192.37	192.35	10.03, s		192.41
HO-4′	-	12.46, s				12.44, s		
HO-6′	^-^	12.88, s				12.89, s		
CH_3_-11				20.86				21.08

* Number of hydrogens bound to carbon atoms deduced by comparative analysis of {^1^H} and APT-^13^C-NMR spectra. Chemical shifts and coupling constants (*J*) obtained from 1D ^1^H-NMR spectrum. Superimposed ^1^H signals are described without multiplicity, and chemical shifts deduced by HMQC, HMBC and ^1^H-^1^H-COSY spectra. All ^1^H and ^13^C chemical shift assignments were also based on homonuclear ^1^H-^1^H-COSY and heteronuclear 2D shift-correlated HMQC (^1^*J*_CH_) and HMBC (*^n^J*_CH_, *n* = 2 and 3) NMR.

**Table 2:** ^1^H (500 MHz) and ^13^C (125 MHz) NMR for **4**, including results obtained by heteronuclear 2D shift-correlated HMQC (^1^*J*_CH_), HMBC (*^n^J*_CH_, *n* = 2 and 3), COSY and NOESY, using CDCl_3_ as solvent. Chemical shifts (δ, ppm) and coupling constants (*J*, Hz) are given in parentheses.

HMQC			HMBC		COSY	NOESY
	δ_C_	δ_H_	^2^*J*_CH_	^3^*J*_CH_	^1^H-^1^H	^1^H-^1^H
C						
1	135.80		H-1′	H-5, H-2′		
3	147.47			H-5, H-7		
4	145.54			H-6, H-2, H-7		
CH						
2	108.85	6.65 brd (1.5)		H-1′		
5	108.05	6.70 d (8.0)				
6	121.08	6.60 dd (8.0, 1.5)		H-2, H-1′		
3′	130.84	5.58–5.52 (m)		H-1′	H-2′	H-5′
4′	130.99	5.99 tt (14.5, 1.0)		H-2′, H-3′, H-6′		
5′	130.08	5.99 tt (14.5, 1.0)		H-7′, H-3′		H-3′, H-7′
6′	133.10	5.58–5.52 m	H-7′		H-7′	
CH_2_						
7	100.69	5.89 s				
1′	35.62	2.59 t (7.5)	H-2′	H-2, H-6, H-3′	H-2′	
2′	34.70	2.30 dt (7.5, 8.0)	H-1′, H-3′	H-4′	H-1′, H-3′	
7′	32.59	2.02 dt (7.0, 7.5)	H-6′	H-5′, H-8′	H-6′, H-8′	H-9′
8′	29.40	1.35–1.31 m	H-7′		H-7′	
9′	31.92	1.29–1.25 m	H-10′	H-11′		H-7′, H-11′
10′	22.68	1.29–1.25 m	H-9′, H-11′		H-11′	
CH_3_						
11′	14.12	0.86 t (7.0)			H-10′	H-9′

## Supplementary Materials

Supplementary File 1
