# Supplementary material for: Isolation of Monoterpene Dihydrochalcones from Piper montealegreanum Yuncker (Piperaceae)
Source: Molecules. 2017 Jun 9;22(6):874. doi: 10.3390/molecules22060874 (PMC6152781; doi:10.3390/molecules22060874)
Supplement: Supplementary File 1 [file molecules-22-00874-s001.pdf]

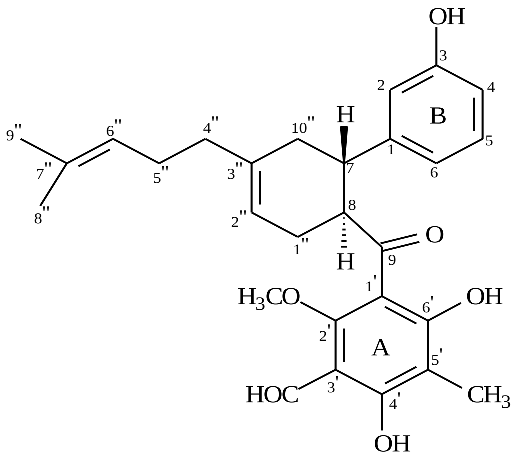

Pmt-1

1 and 3 (M.M = 464u)

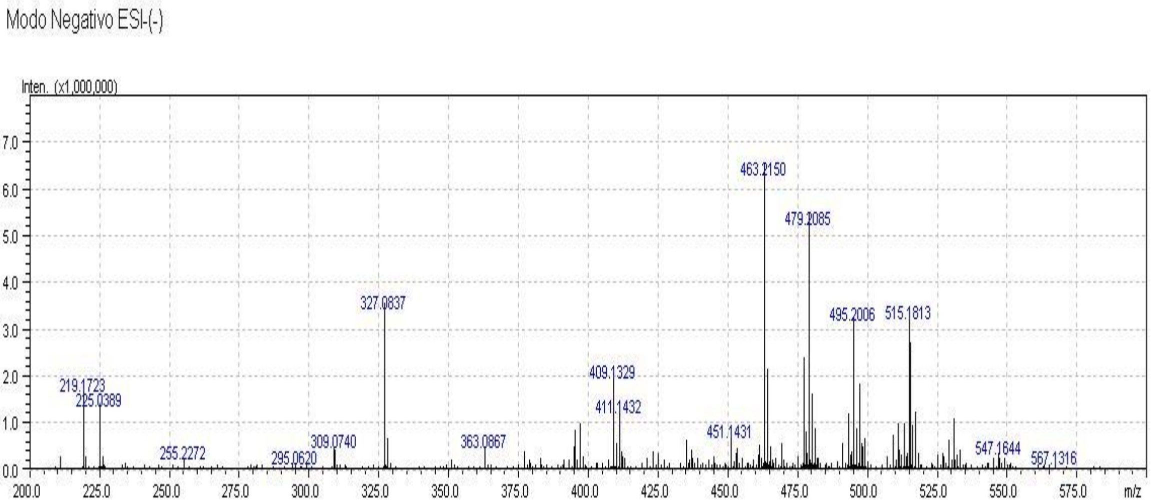

Espectro de massas de alta resolução (EMAR) da Pmt-1:

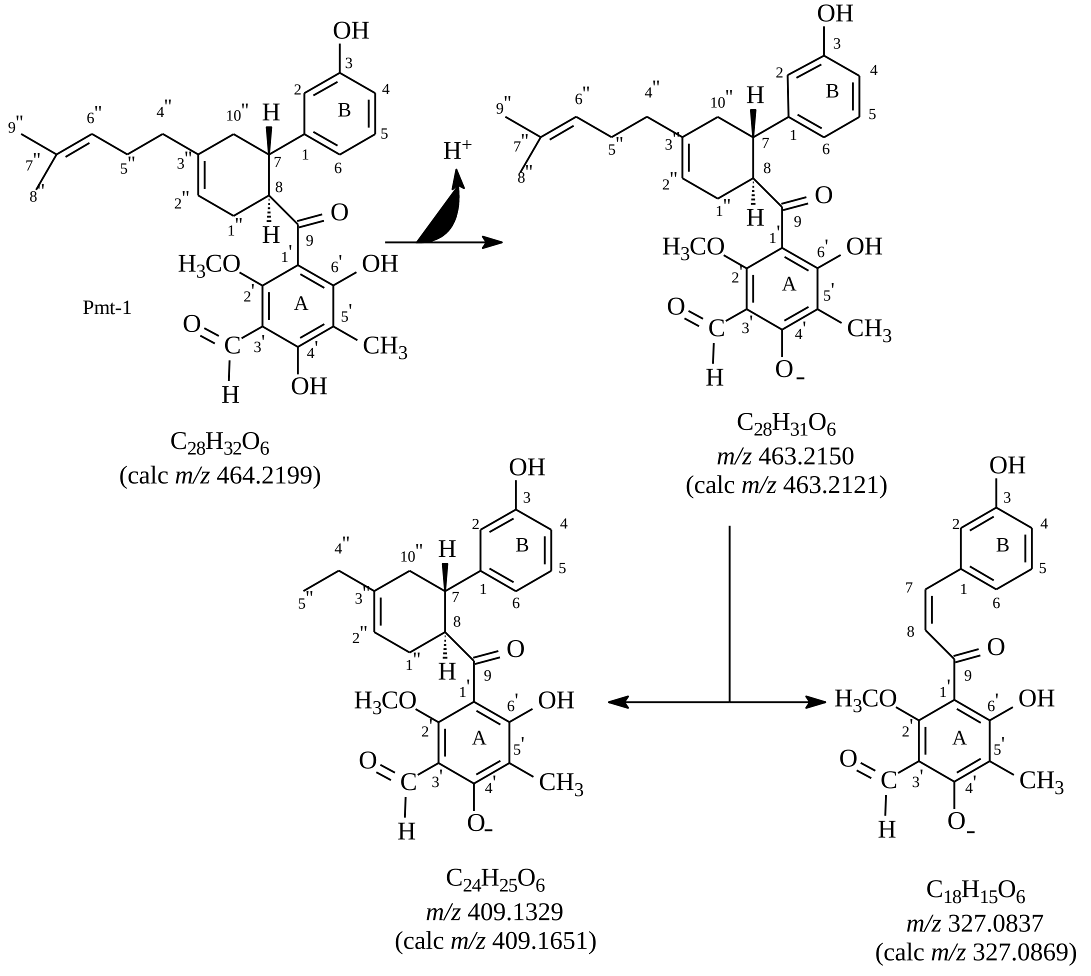

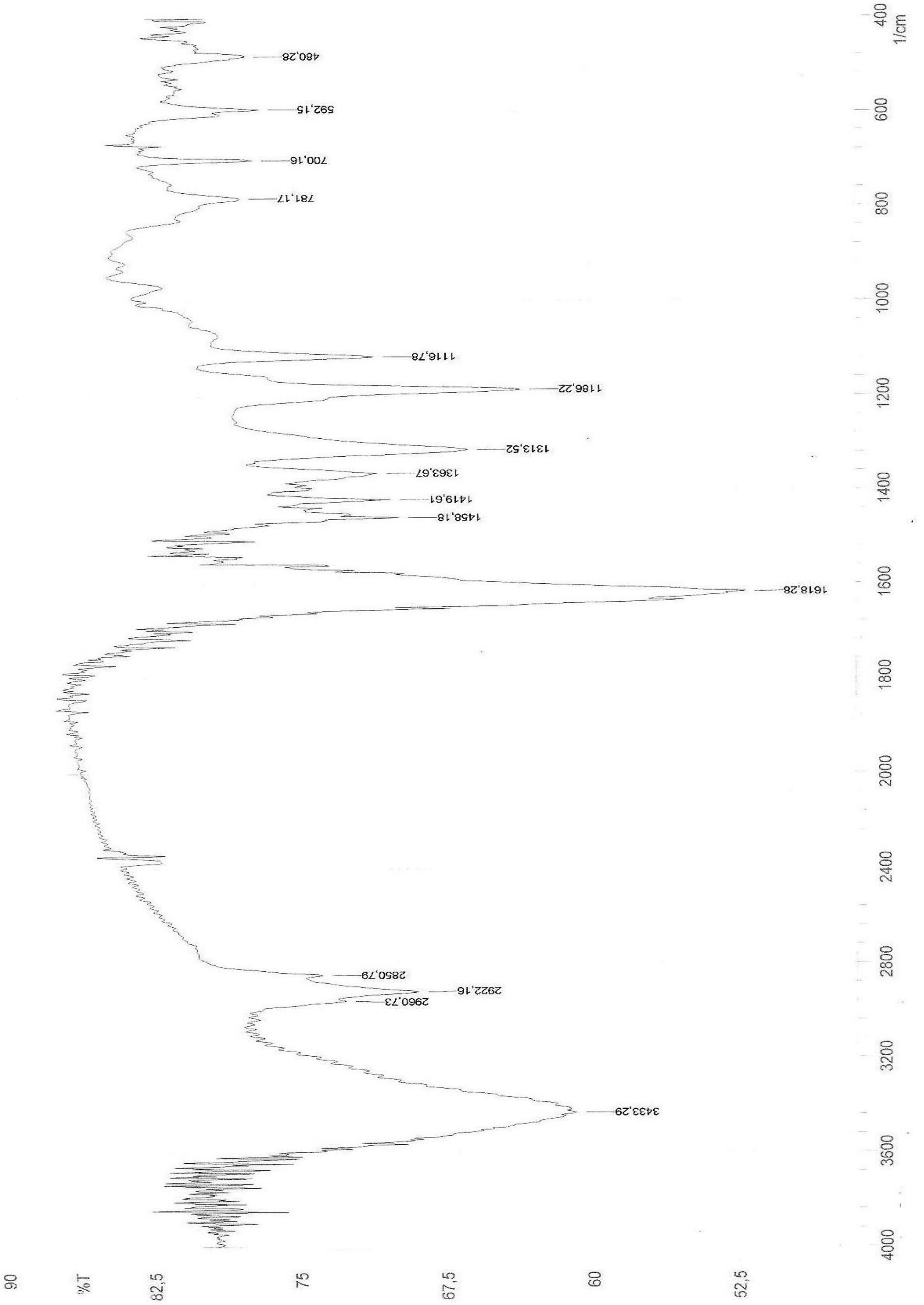

Figura 8: espectro no Infravermelho (λ_{max}, KBr, cm^{-1}) de Pmt-1

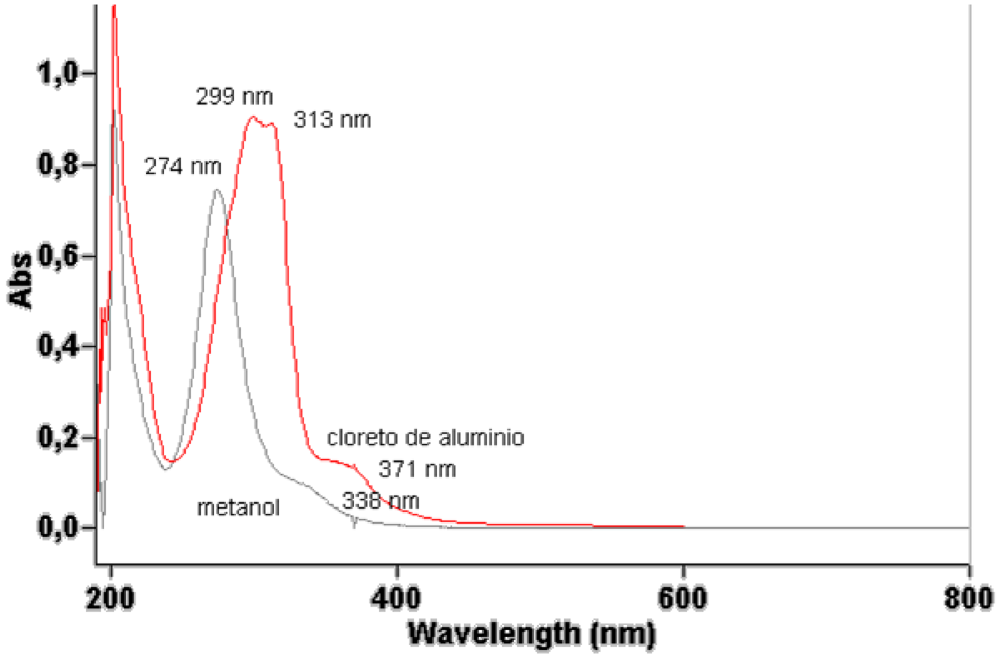

Figura 9: espectro no ultravioleta ($\lambda_{max.}$, MeOH; MeOH + $AlCl_3$) de Pmt-1

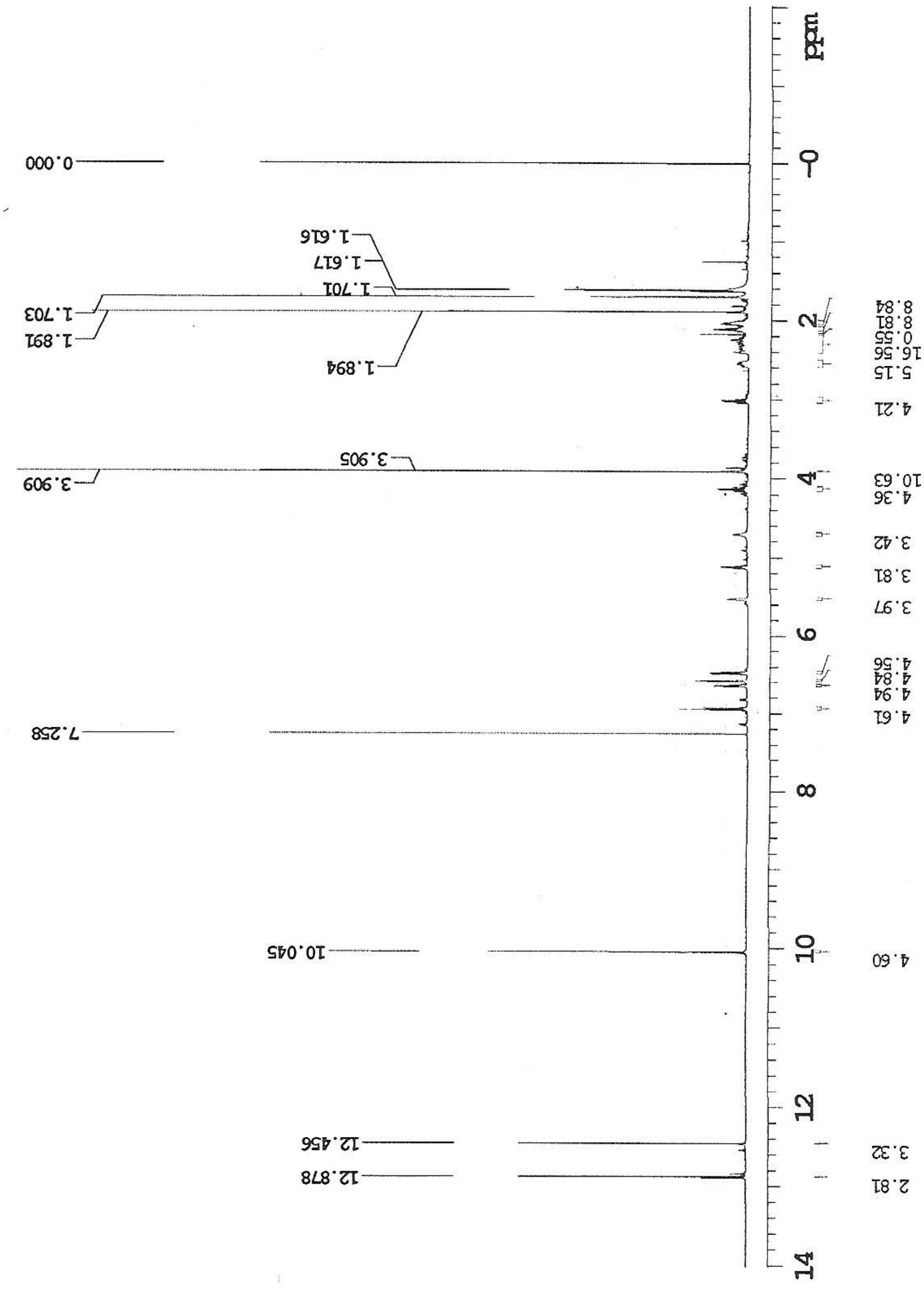

Figura 10 – Espectro de RMN ^{1}H (δ, $CDCl_3$, 500 MHz) de Pmt-1

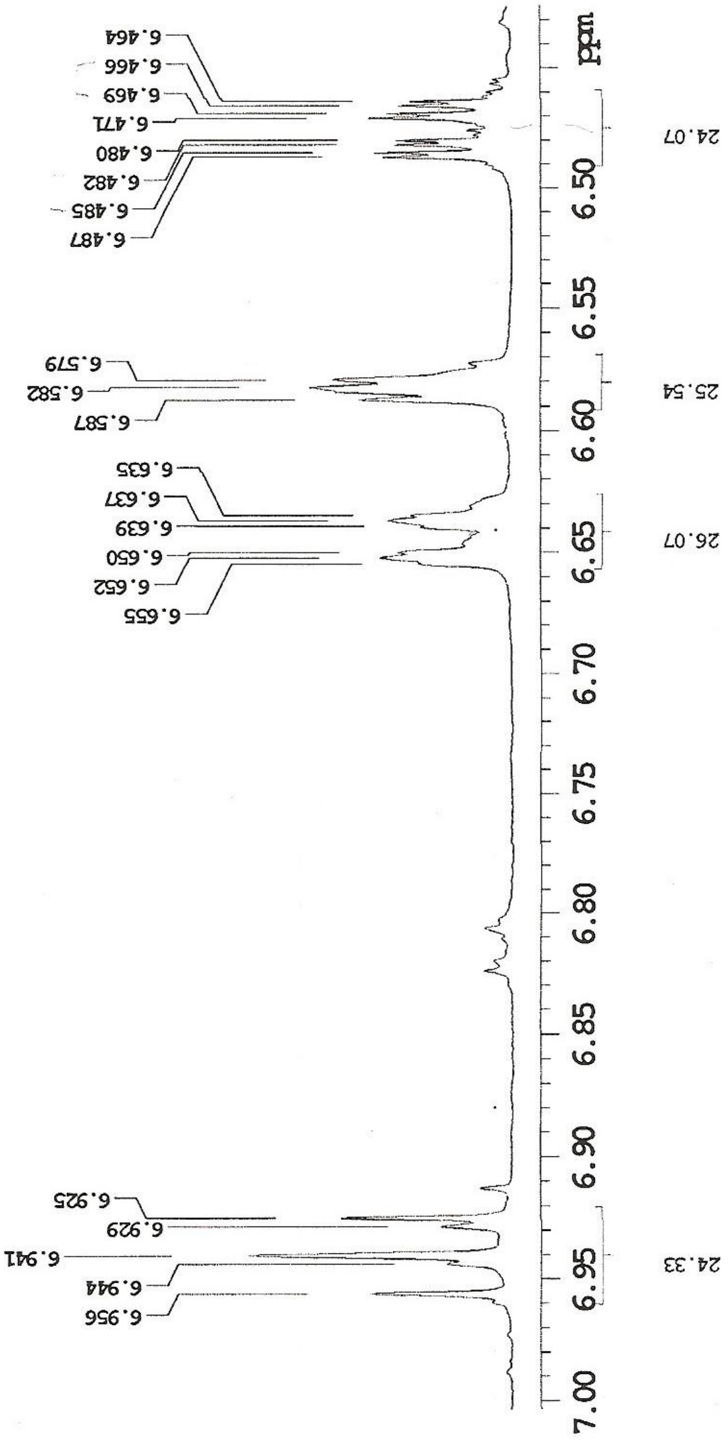

Figura 11 – expansão do espectro de RMN ^{1}H (δ, $CDCl_3$, 500 MHz) de Pmt-1

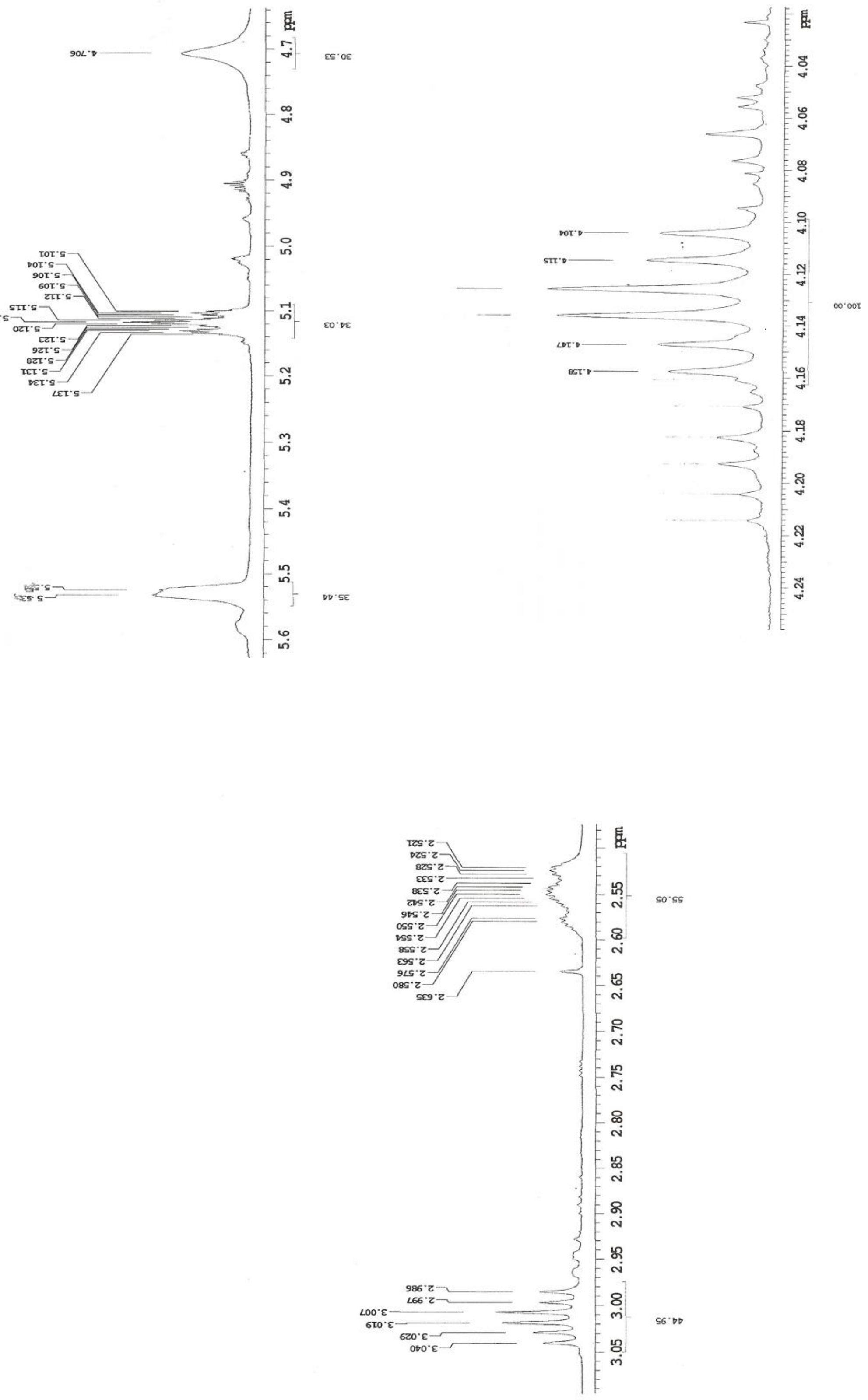

Figura 12, 13 e 14 – expansões do espectro de RMN ^{1}H (δ, $CDCl_3$, 500 MHz) de Pmt-1

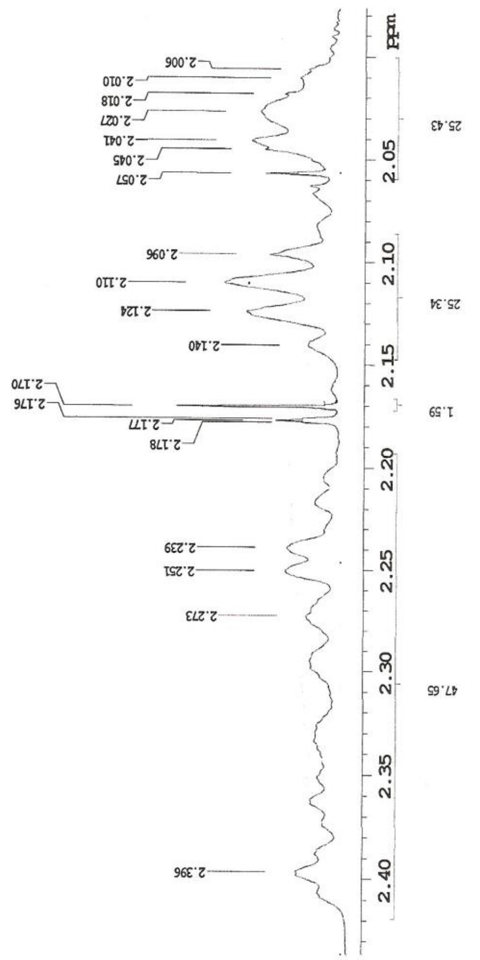

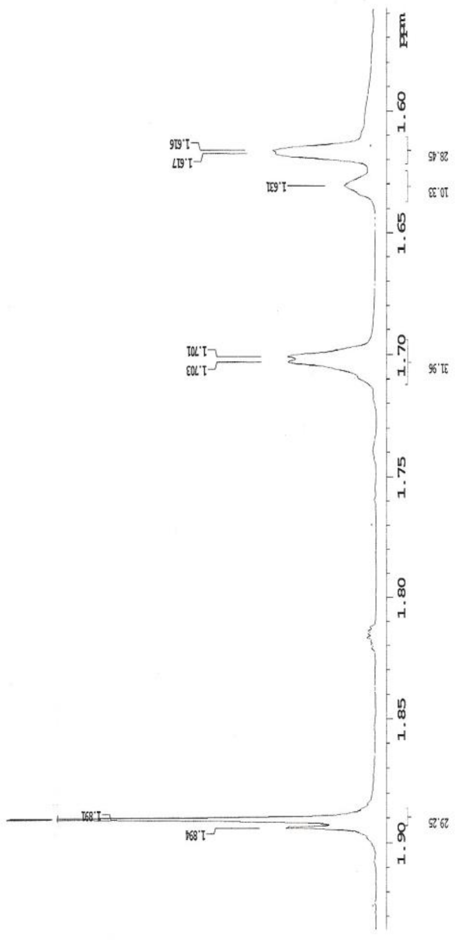

Figura 15 e 16 – expansões do espectro de RMN ^{1}H (δ, $CDCl_3$, 500 MHz) de Pmt-1

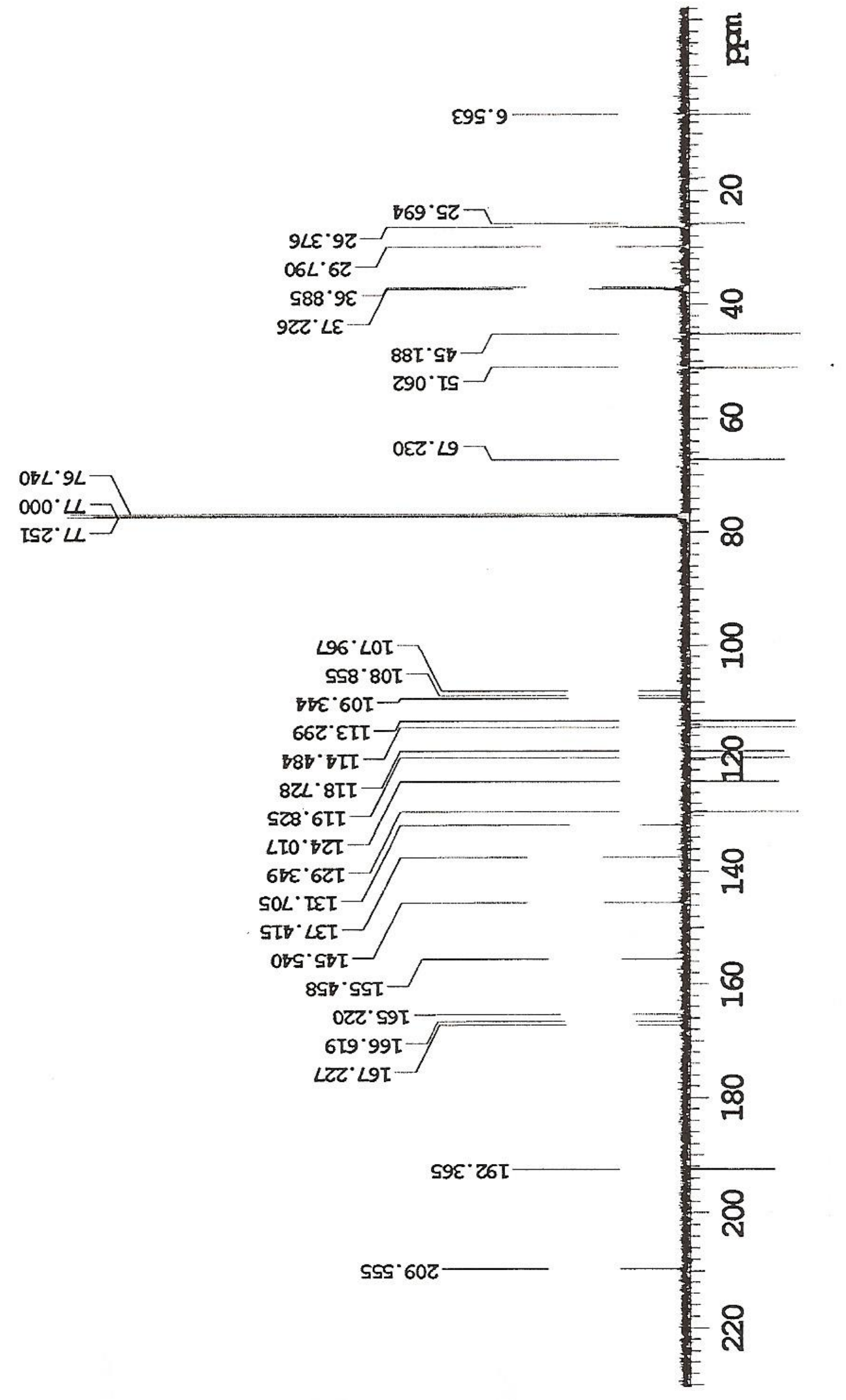

Figura 17 – espectro de RMN de ^{13}C, técnica APT (δ, $CDCl_3$, 125 MHz) de Pmt-1

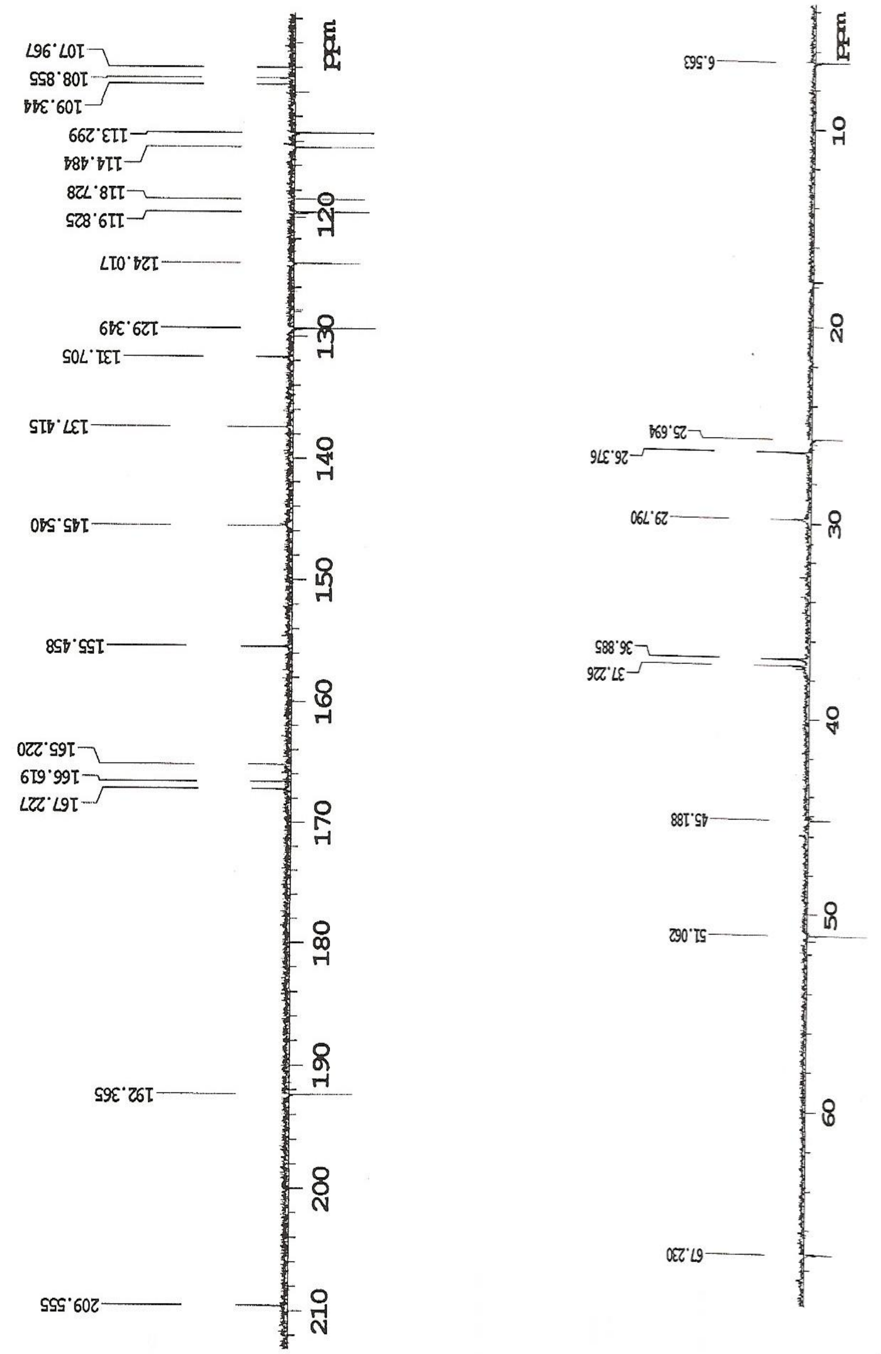

Figura 18 e 19 – expansões do espectro de RMN de ^{13}C (δ, $CDCl_3$, 125 MHz) de Pmt-1

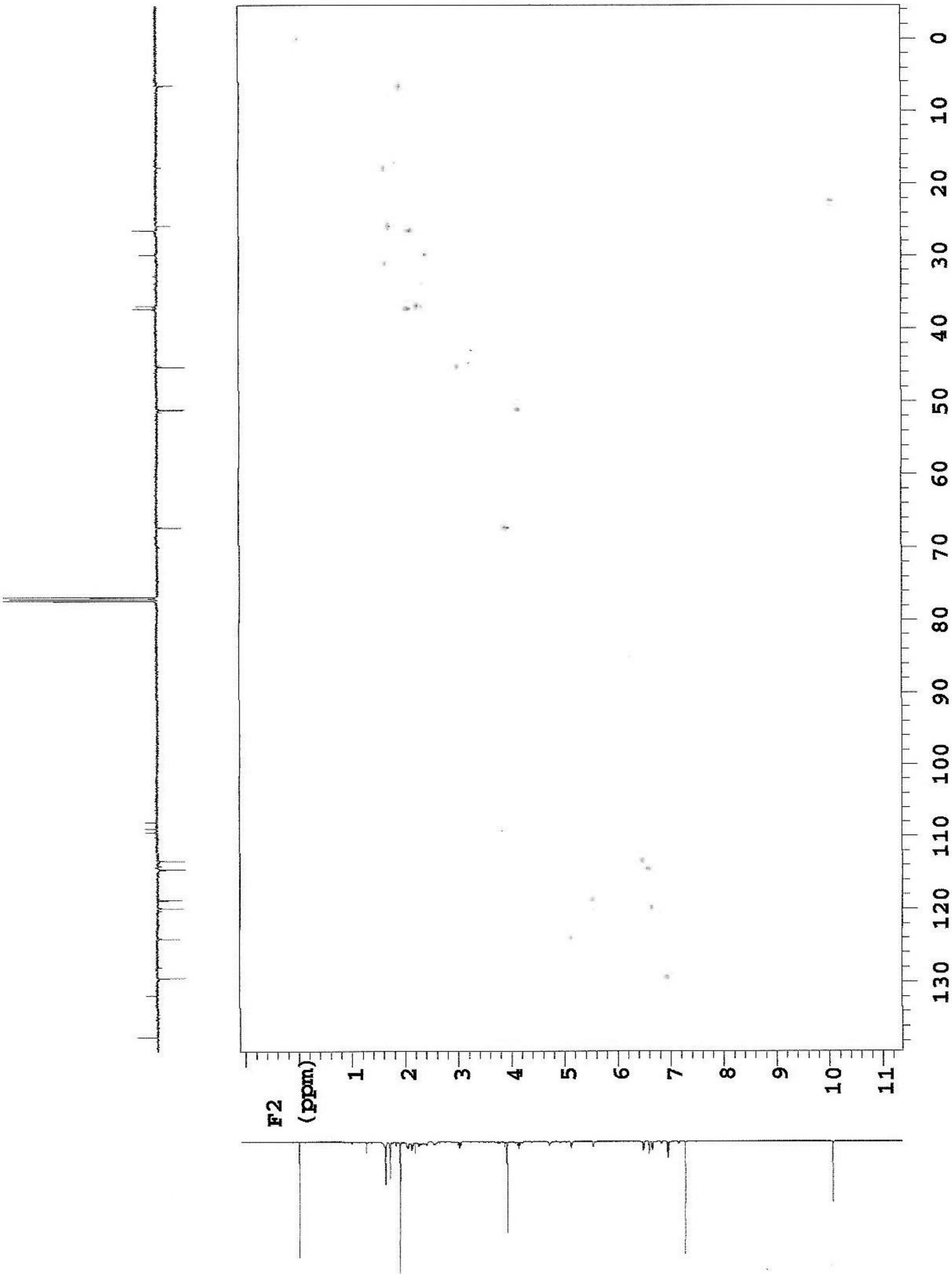

Figura 20 – espectro de correlação heteronuclear HMQC – ^{1}H x ^{13}C de Pmt-1

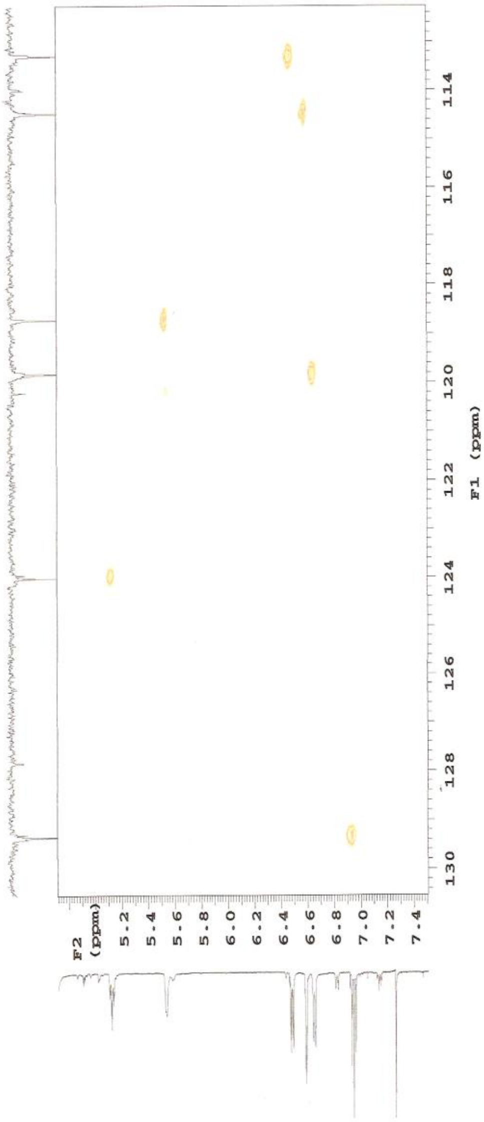

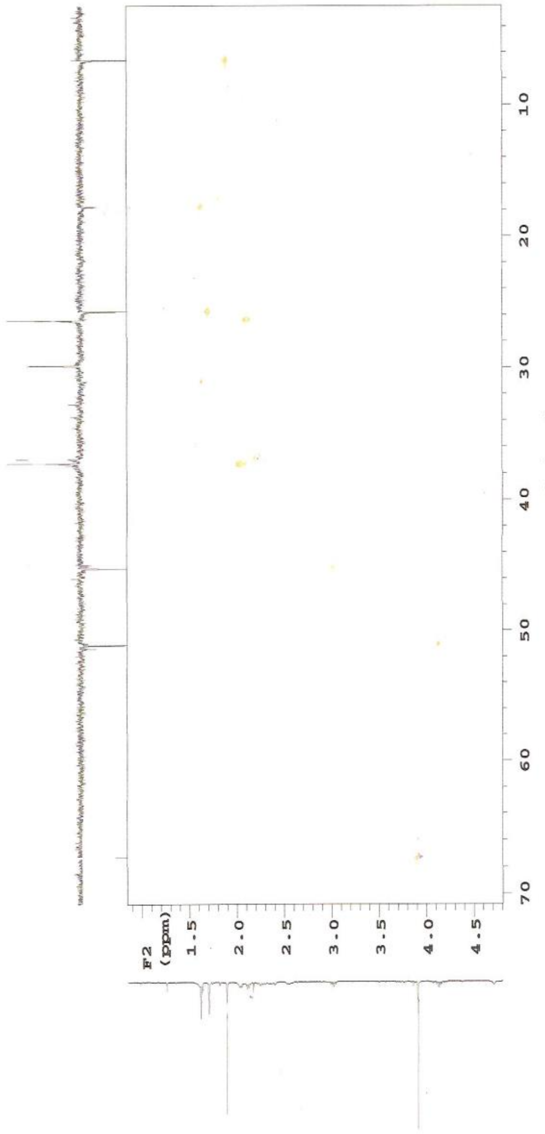

Figura 21 e 22 – expansões do espectro de correlação heteronuclear HMQC – ^{1}H x ^{13}C de Pmt-1

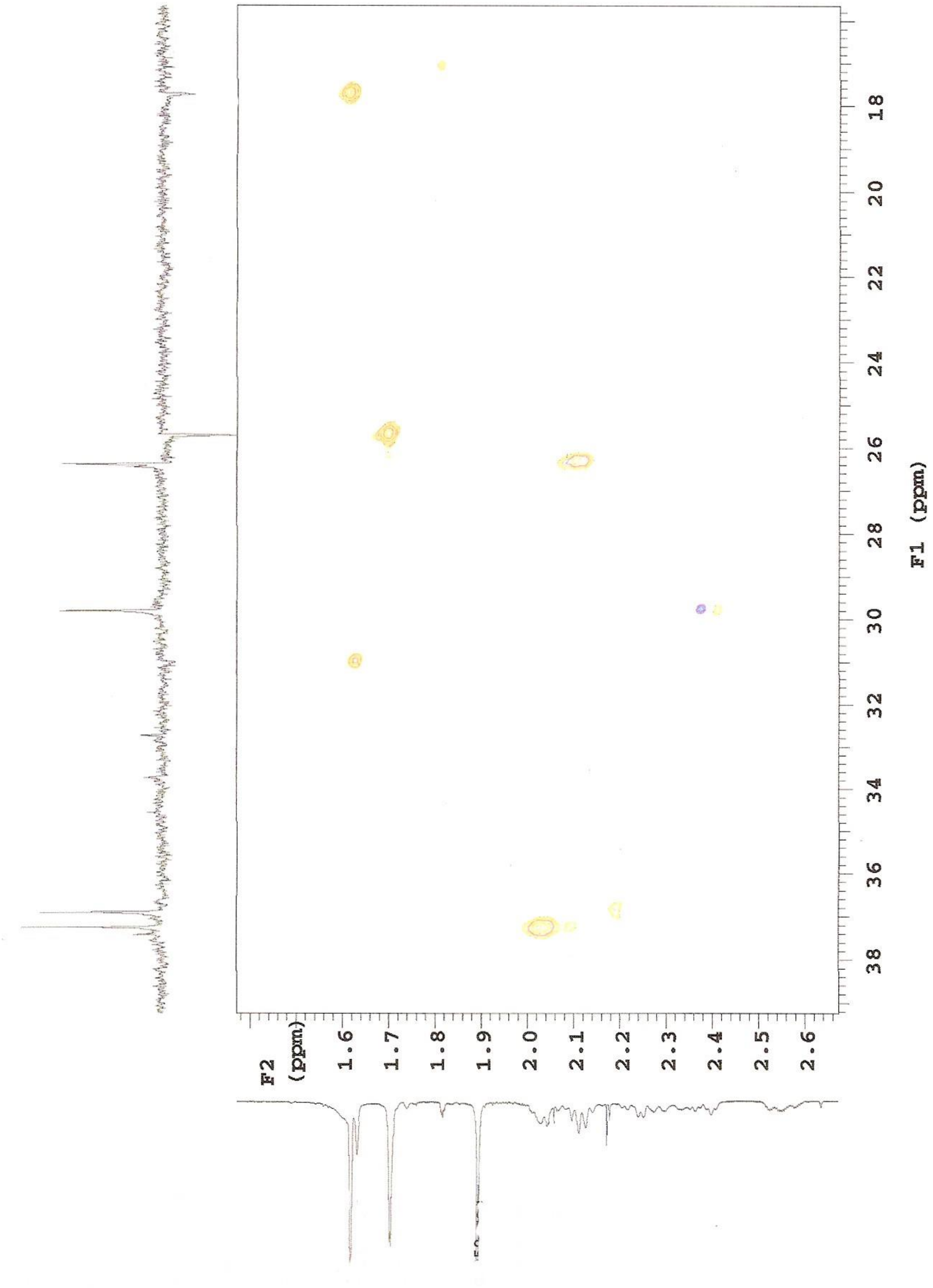

Figura 23 – expansão do espectro de correlação heteronuclear HMQC – ^{1}H x ^{13}C de Pmt-1

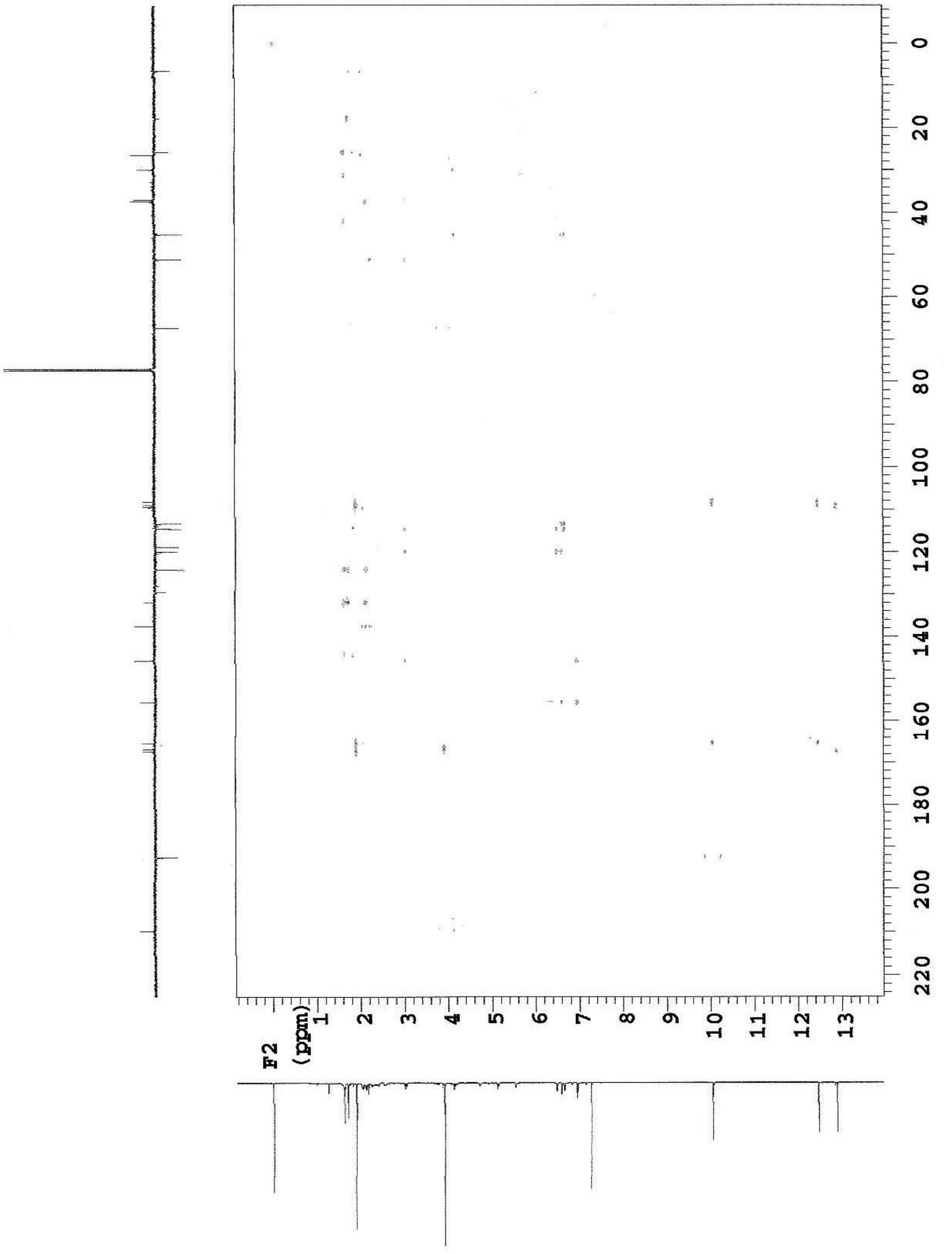

Figura 24 – espectro de correlação heteronuclear HMBC – ^{1}H x ^{13}C de Pmt-1

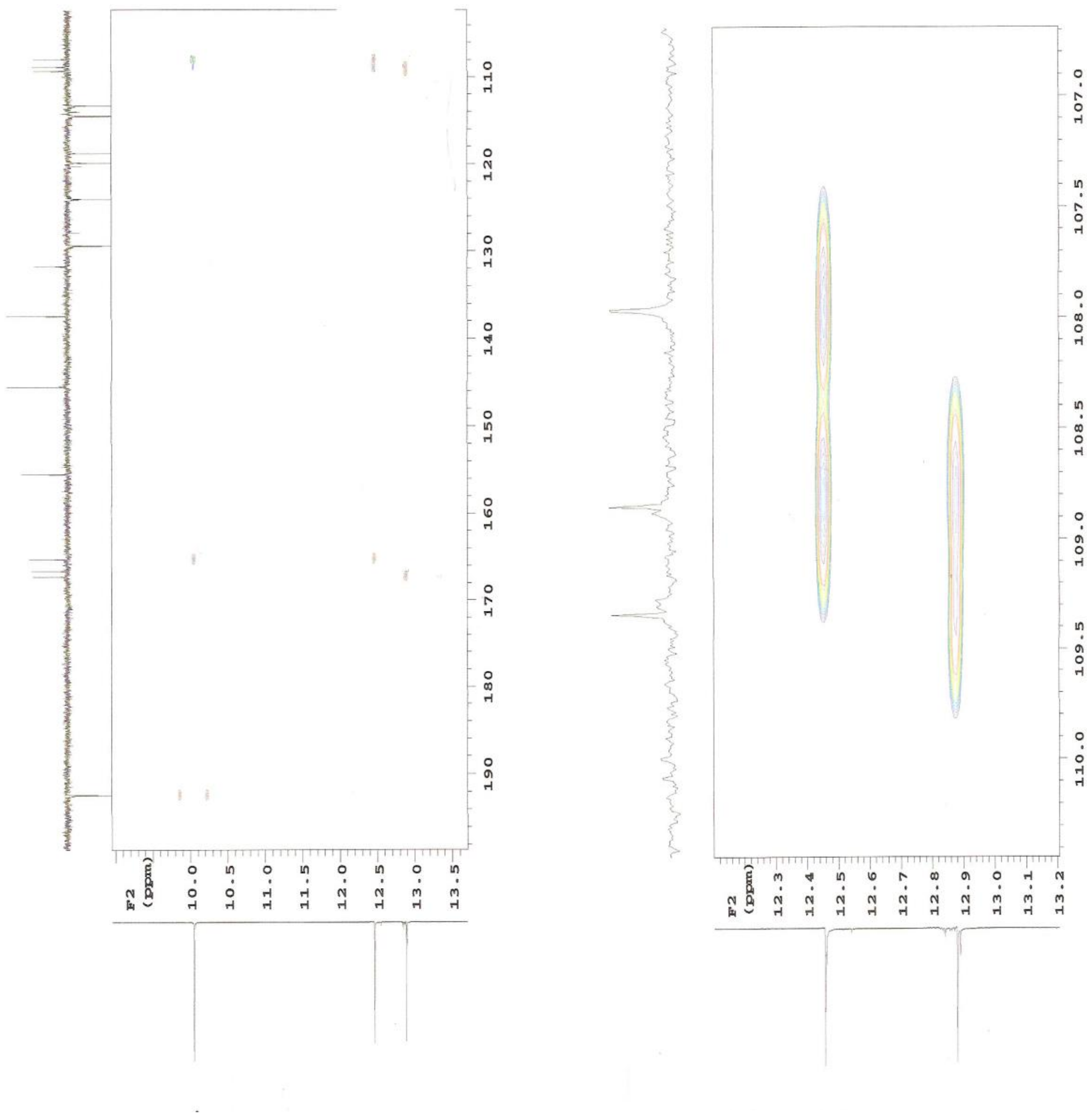

Figura 25 e 26 – expansões do espectro de correlação heteronuclear HMBC – ^{1}H x ^{13}C de Pmt-1

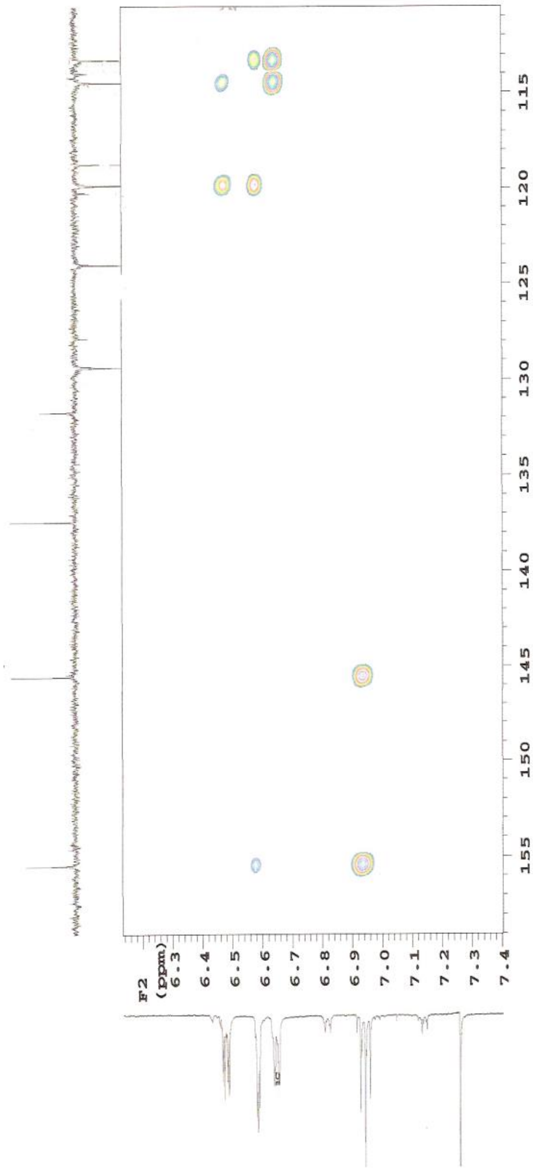

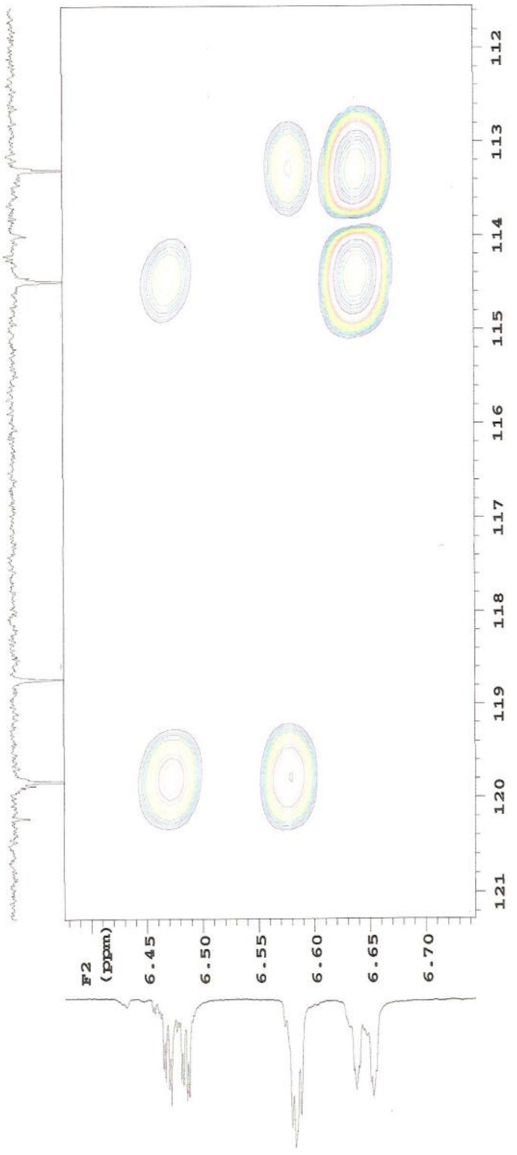

Figura 27 e 28 – expansões do espectro de correlação heteronuclear HMBC – ^1H x ^{13}C de Pmt-1

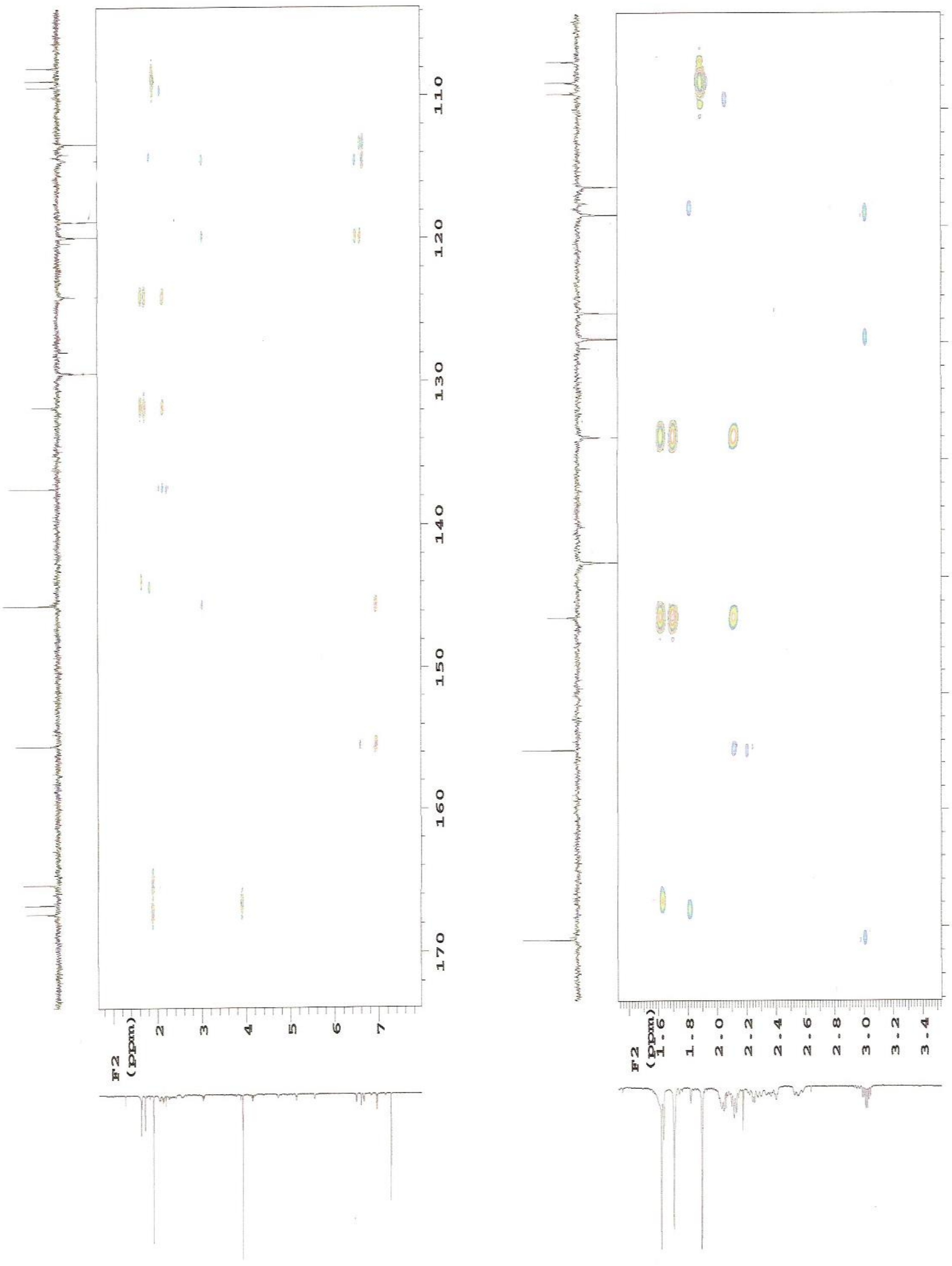

Figura 29 e 30 – expansões do espectro de correlação heteronuclear HMBC – ^{1}H x ^{13}C de Pmt-1

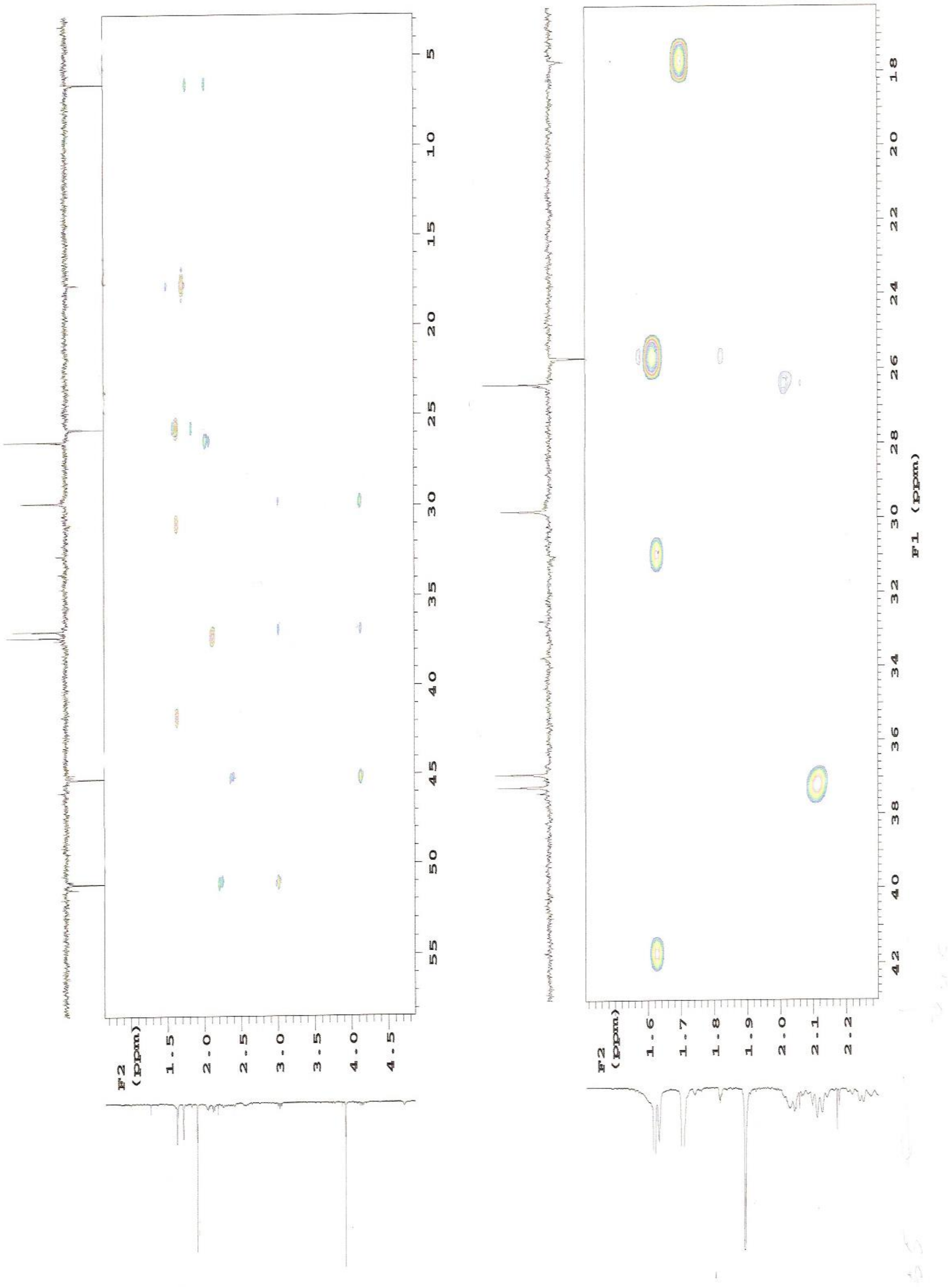

Figura 31 e 32 – expansões do espectro de correlação heteronuclear HMBC – ^{1}H x ^{13}C de Pmt-1

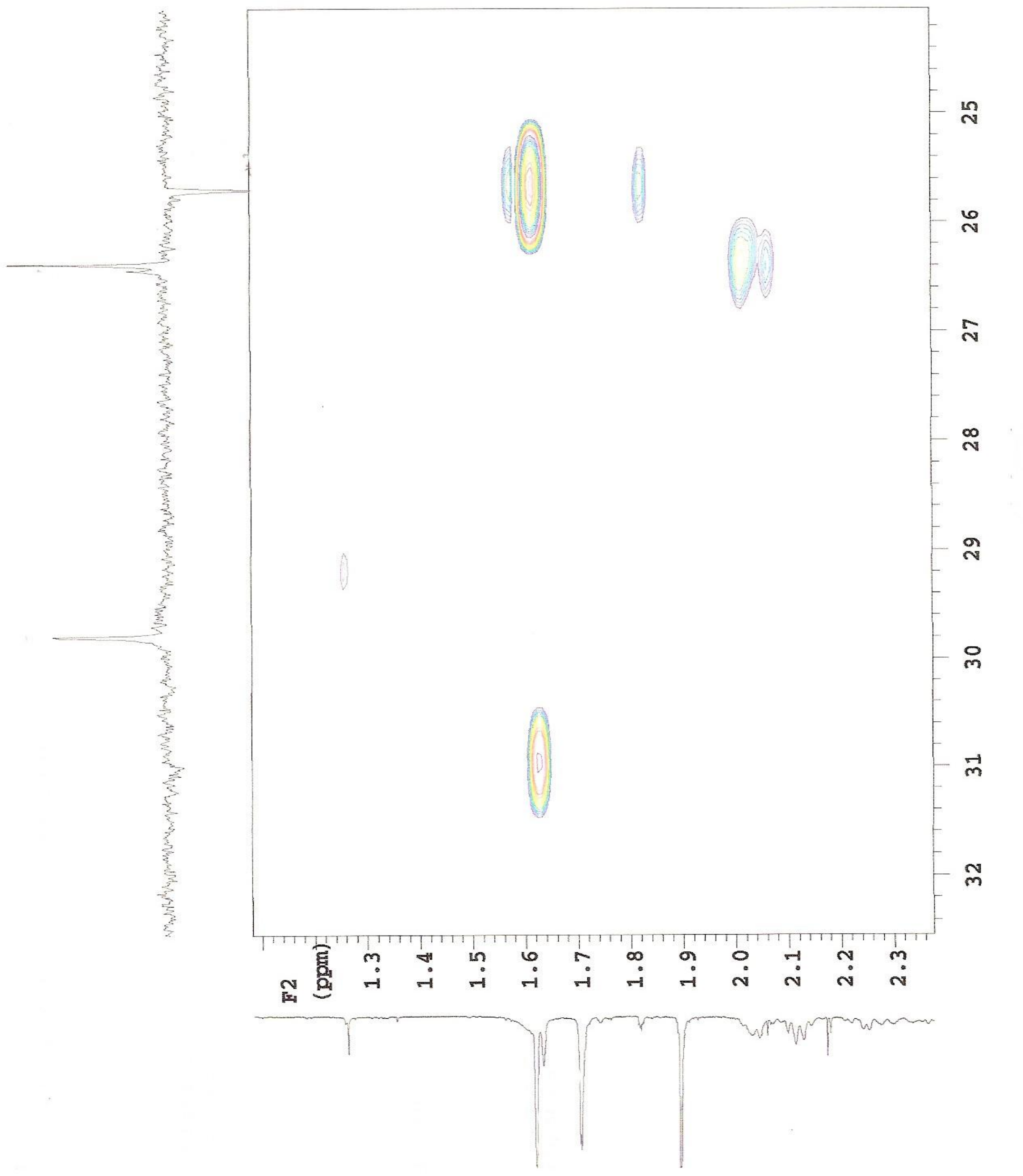

Figura 33 – expansão do espectro de correlação heteronuclear HMBC – ^{1}H x ^{13}C de Pmt-1

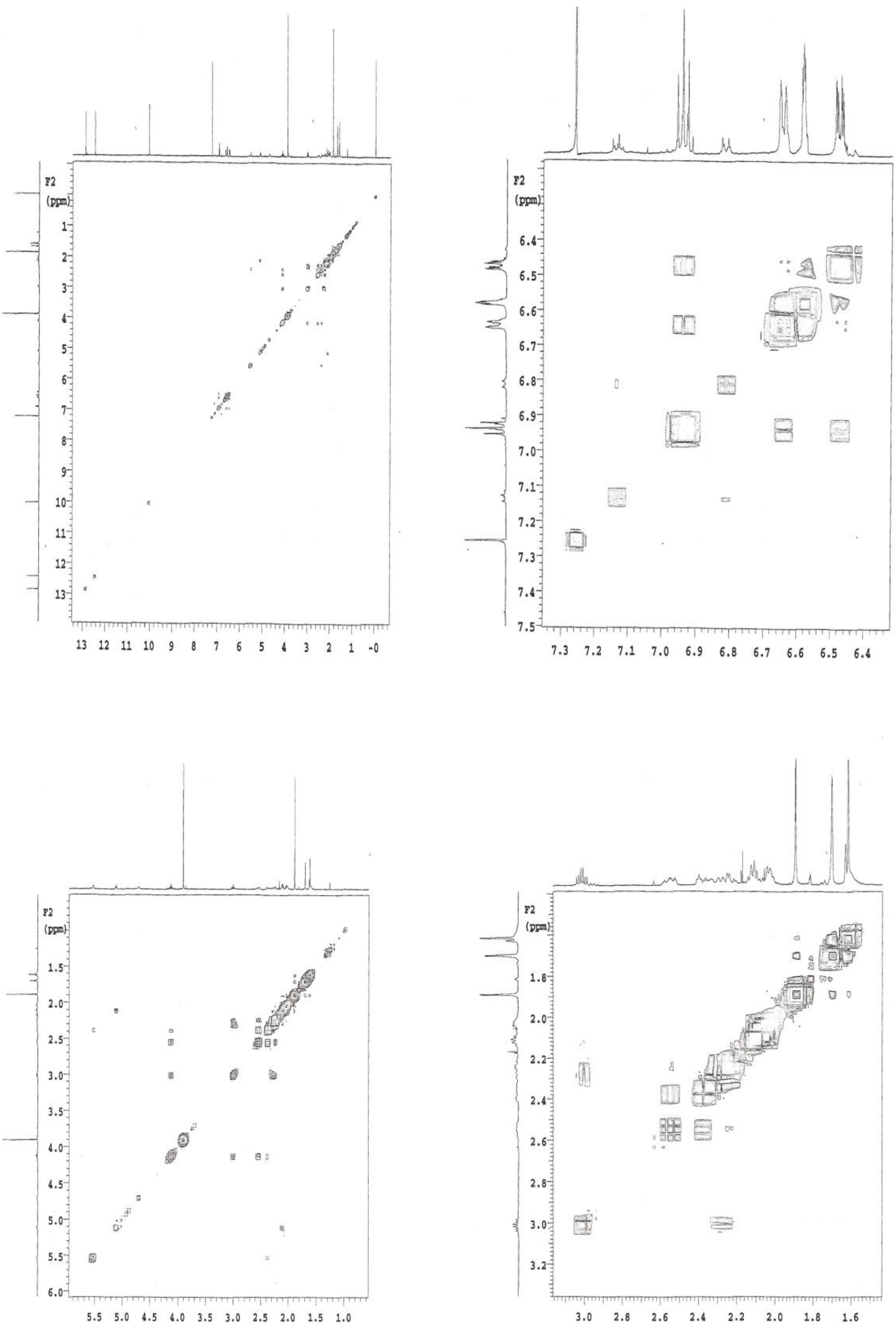

Figura 34-37 – expansões do espectro de correlação homonuclear COSY – ^{1}H x ^{1}H de Pmt-1

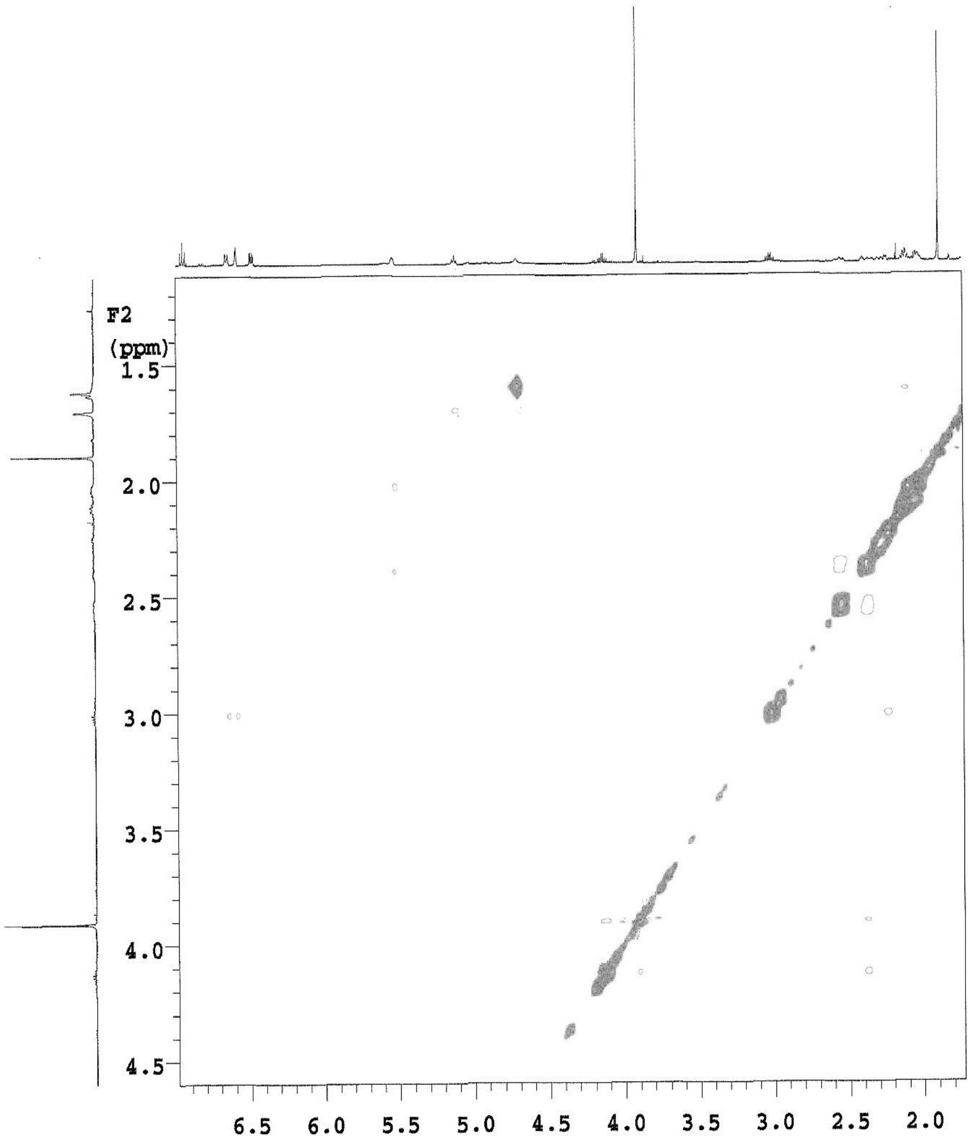

Figura 38: expansão do espectro de correlação homonuclear NOESY – ^{1}H x ^{1}H de Pmt-1

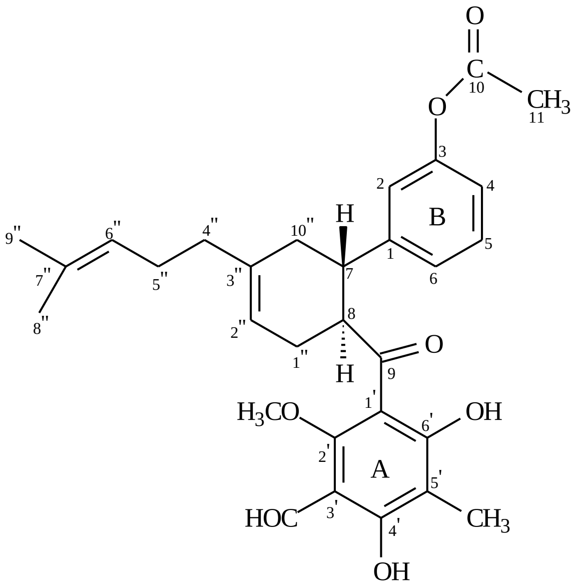

Pmt-1 ACETILADO

Sample: Pmonte-C2.10.1.4-Fr3acet.
File: xp

Pulse Sequence: s2pul

Solvent: cdcl3
Temp. 27.0 C / 300.1 K
Operator: vnmr1
VNMRS-500 "varian500.1tf.ufpb.br"

Relax. delay 0.847 sec
Pulse 45.0 degrees
Acq. time 4.153 sec
Width 8012.8 Hz
16 repetitions
OBSERVE H1, 499.5804893 MHz
DATA PROCESSING
Resol. enhancement -0.0 Hz
FT size 131072
Total time 1 min, 30 sec

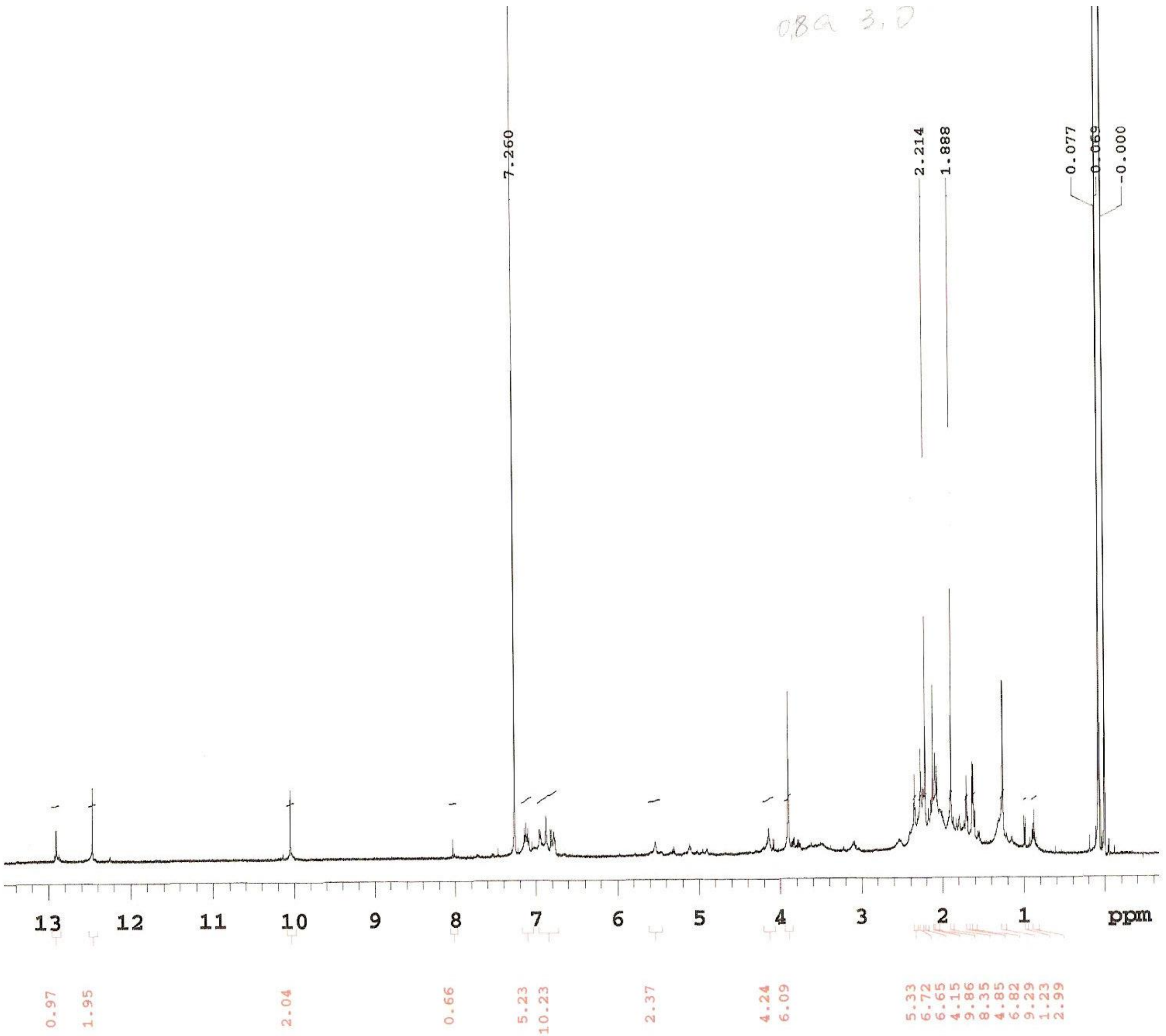

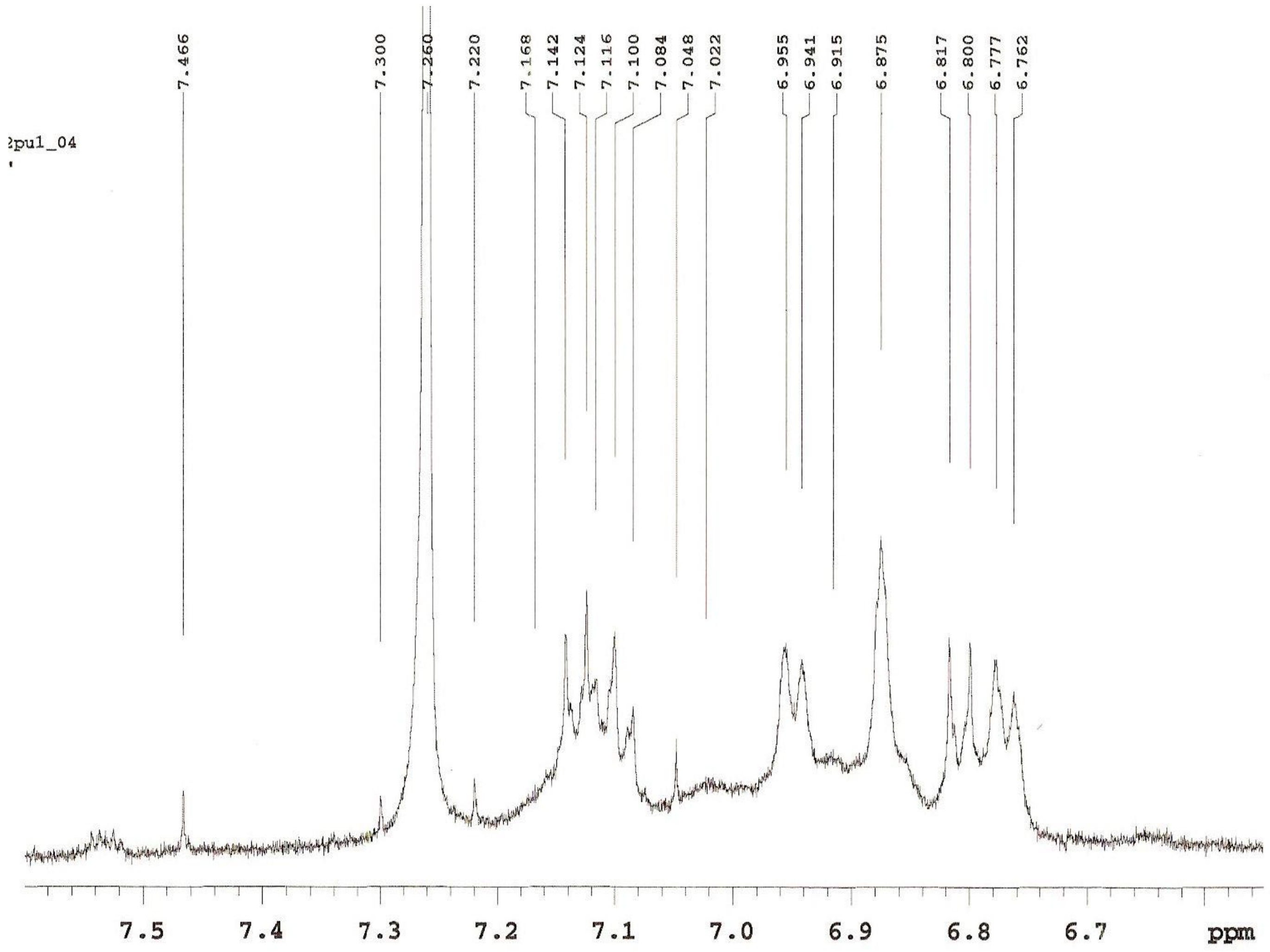

2pu1_04
7.466
7.300
7.260
7.220
7.168
7.142
7.124
7.116
7.100
7.084
7.048
7.022
6.955
6.941
6.915
6.875
6.817
6.800
6.777
6.762
7.5
7.4
7.3
7.2
7.1
7.0
6.9
6.8
6.7
ppm

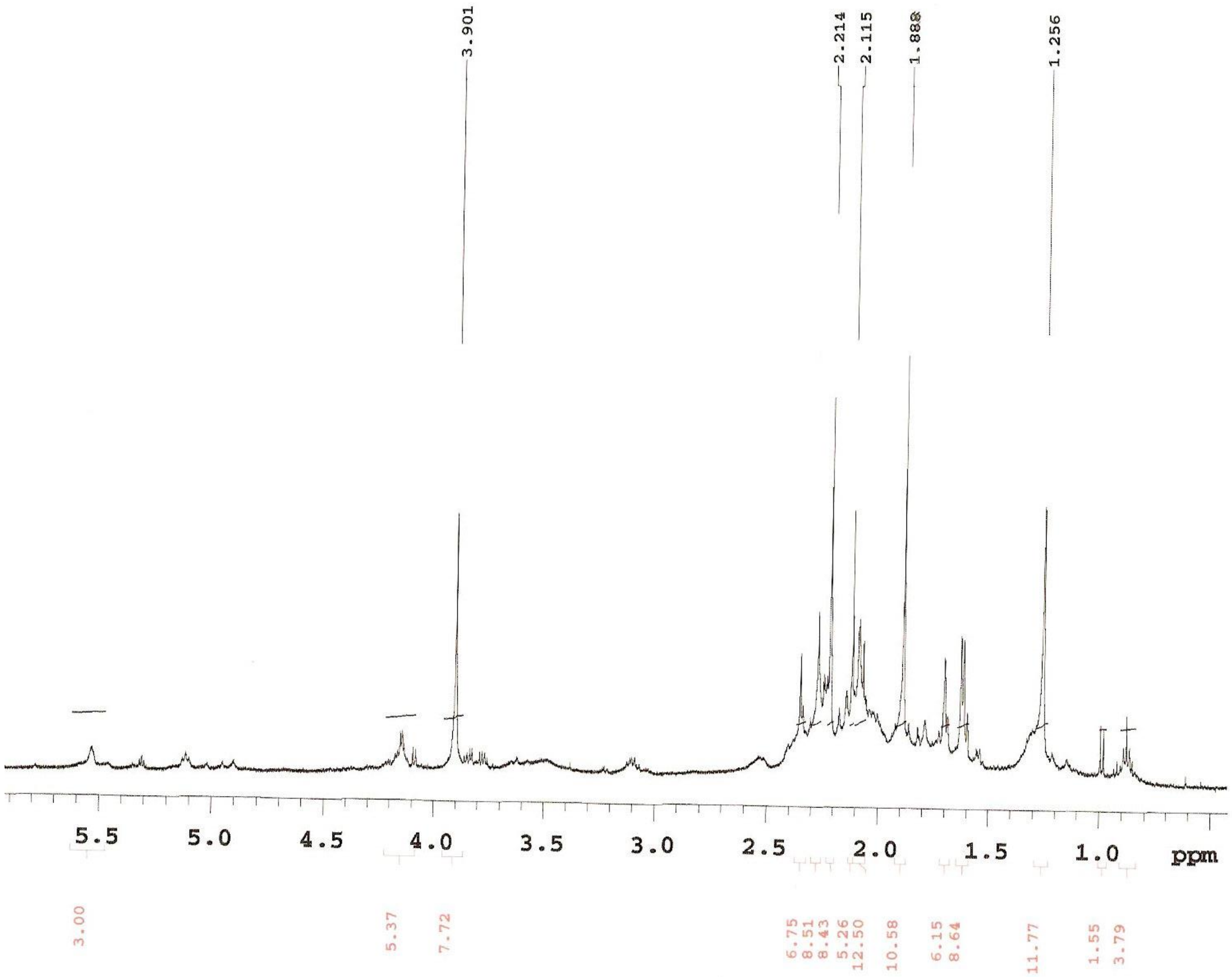

3.901
2.214
2.115
1.888
1.256
5.5
5.0
4.5
4.0
3.5
3.0
2.5
2.0
1.5
1.0
ppm
3.00
5.37
7.72
6.75
8.51
8.43
5.26
12.50
10.58
6.15
8.64
11.77
1.55
3.79

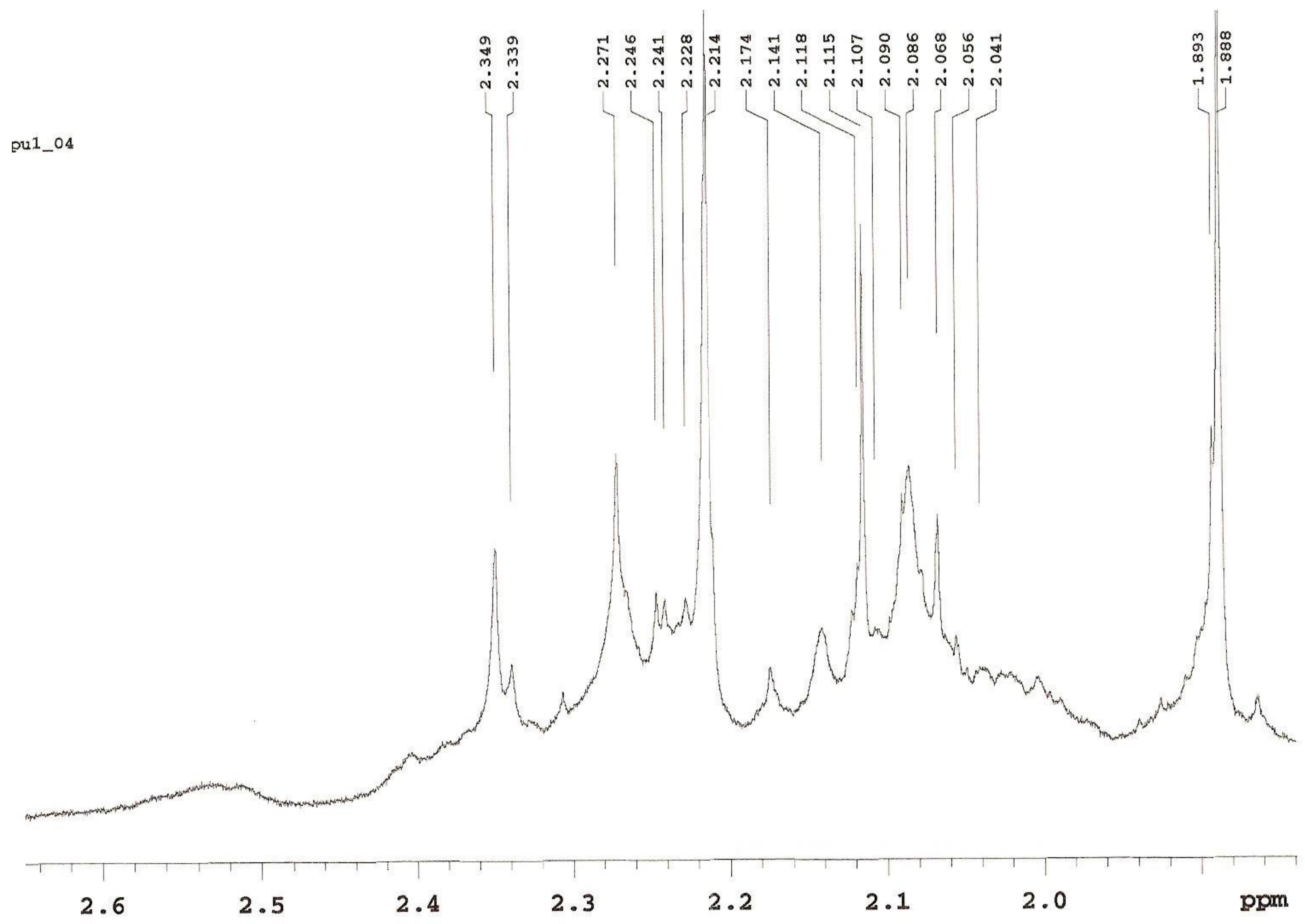
pu1_04
2.349
2.339
2.271
2.246
2.241
2.228
2.214
2.174
2.141
2.118
2.115
2.107
2.090
2.086
2.068
2.056
2.041
1.893
1.888
2.6
2.5
2.4
2.3
2.2
2.1
2.0
ppm

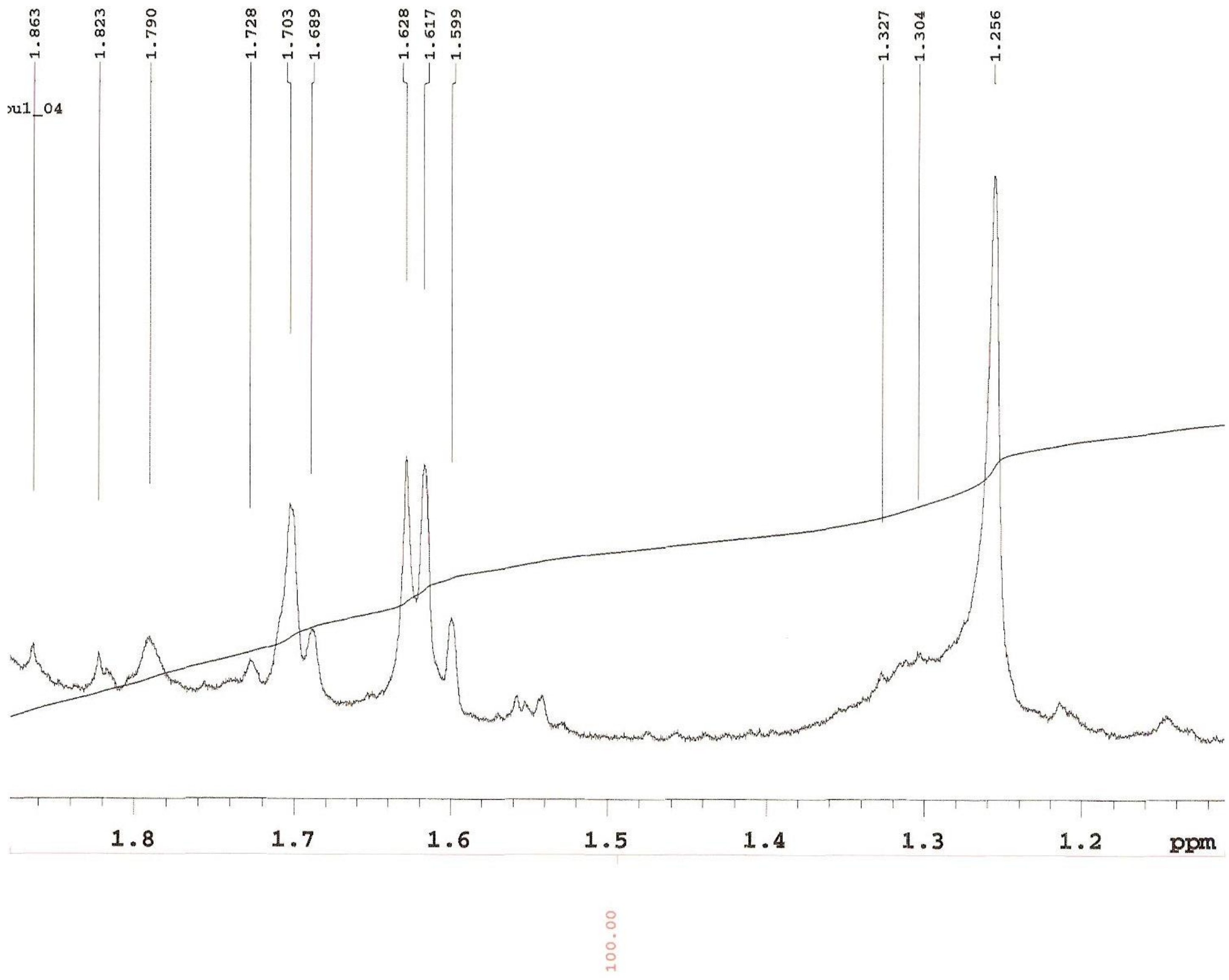
1.863
1.823
1.790
1.728
1.703
1.689
1.628
1.617
1.599
1.327
1.304
1.256
ou1_04
1.8
1.7
1.6
1.5
1.4
1.3
1.2
ppm
100.00

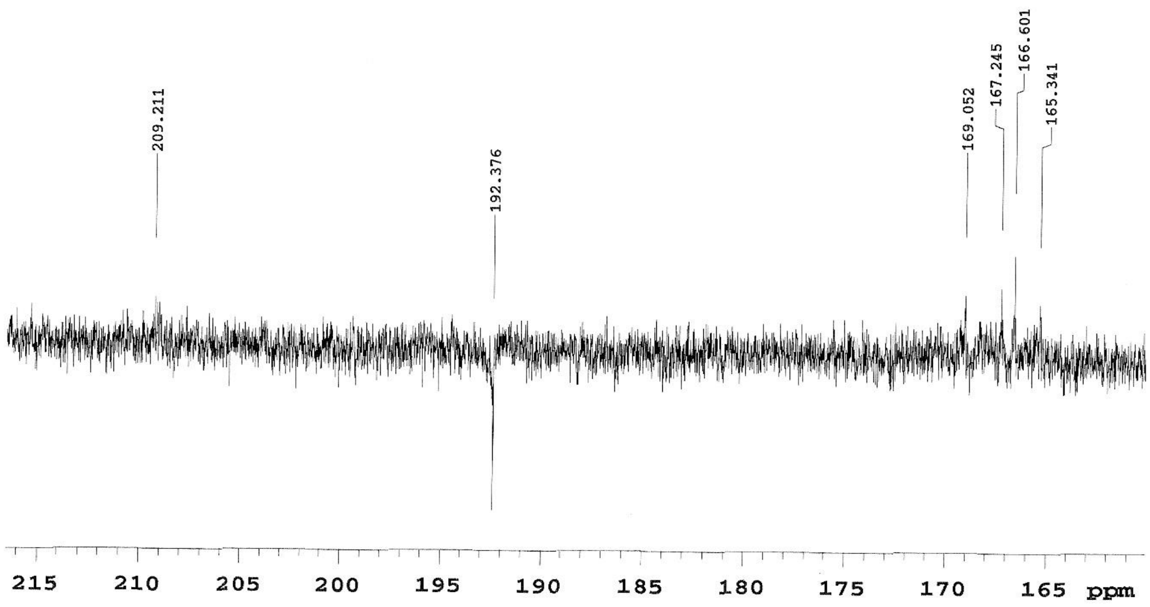

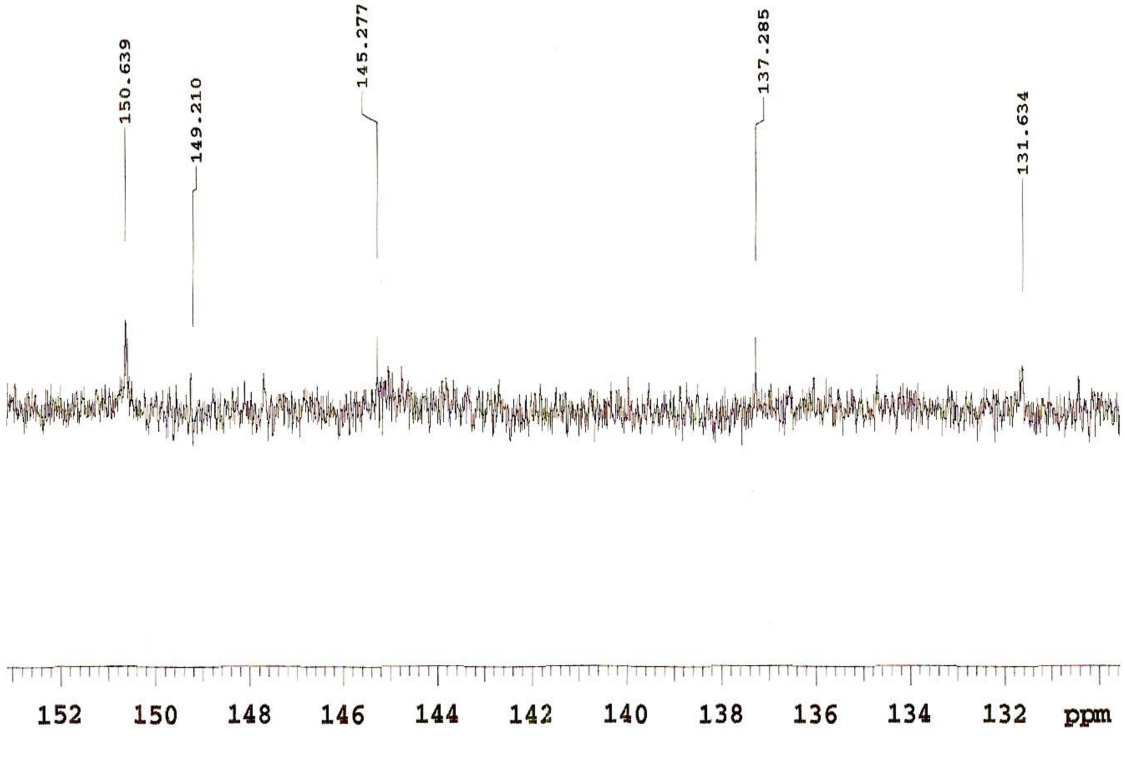

Figuras 39 e 40 – expansões do espectro de RMN de ^{13}C (APT) de Pmt-1 acetilado

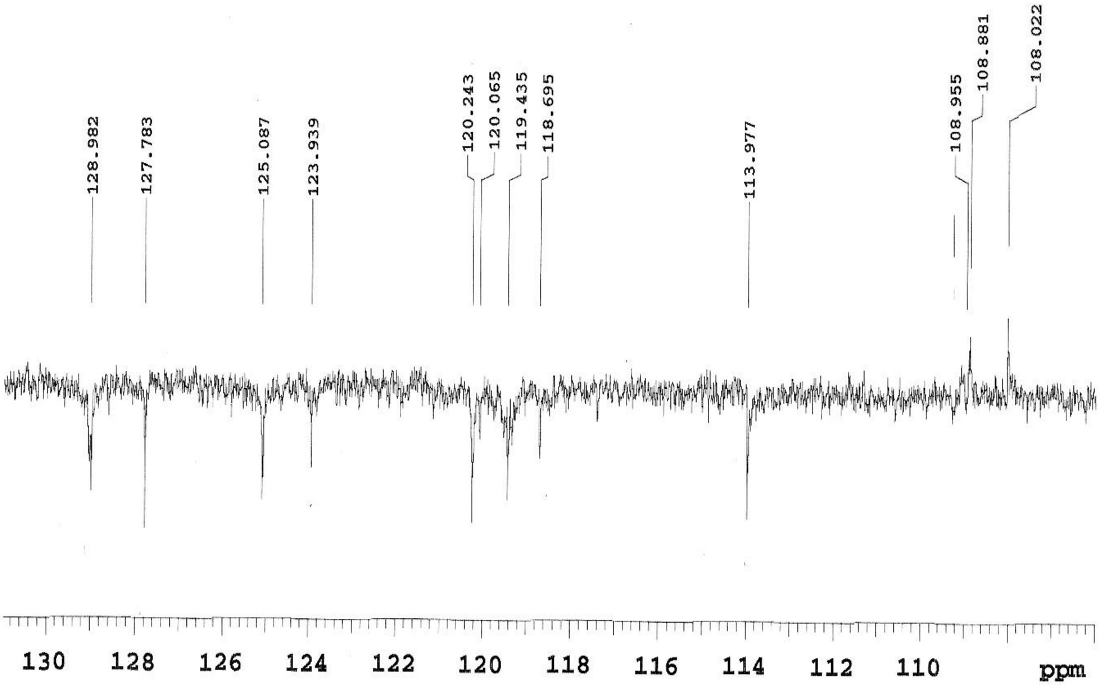

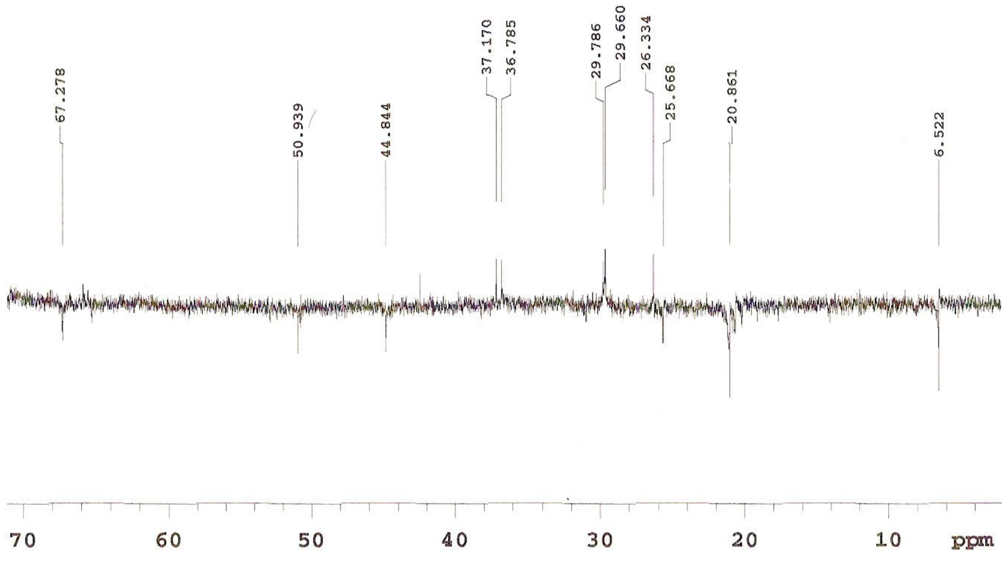

Figuras 41 e 42 – expansões do espectro de RMN de ^{13}C (APT) de Pmt-1 acetilado

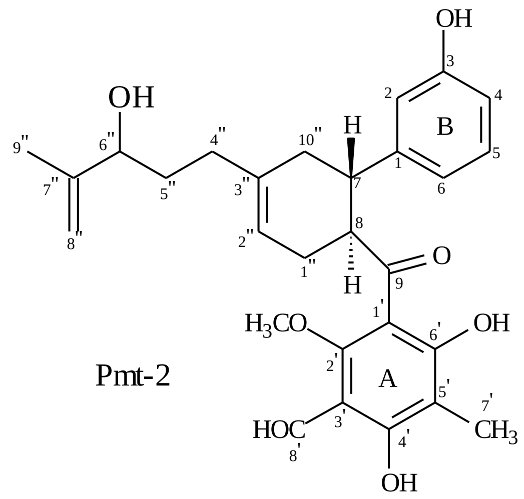

2 (M.M. = 480u)

Modo Negativo ESI-(-)

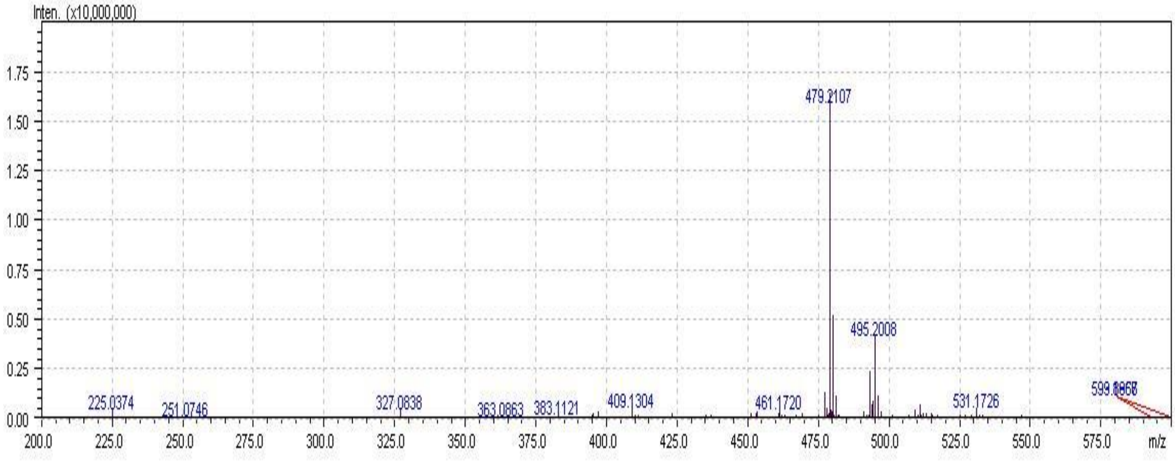

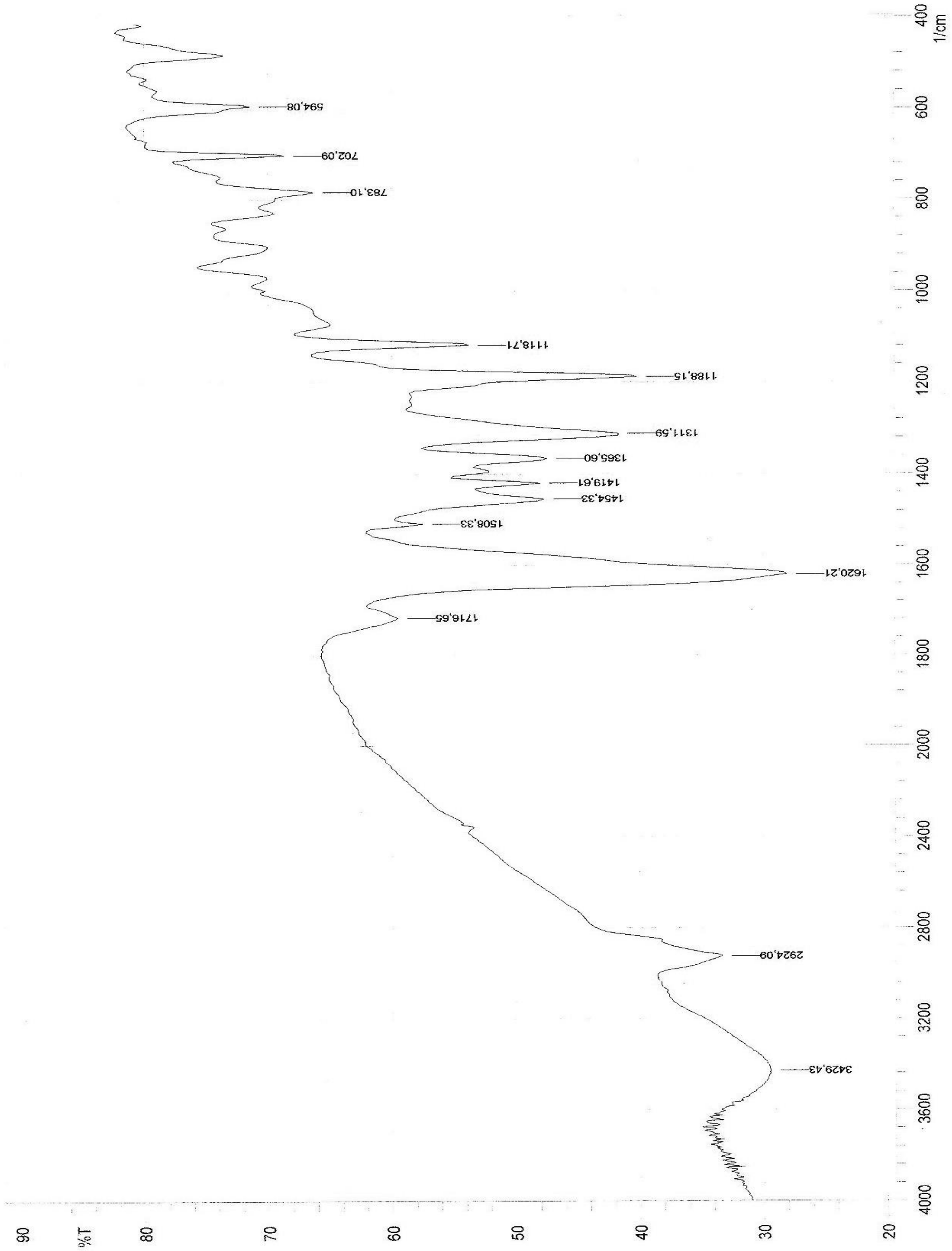

Figura 43: espectro no Infravermelho ($\lambda_{max,}$ KBr, cm^{-1}) de Pmt-2

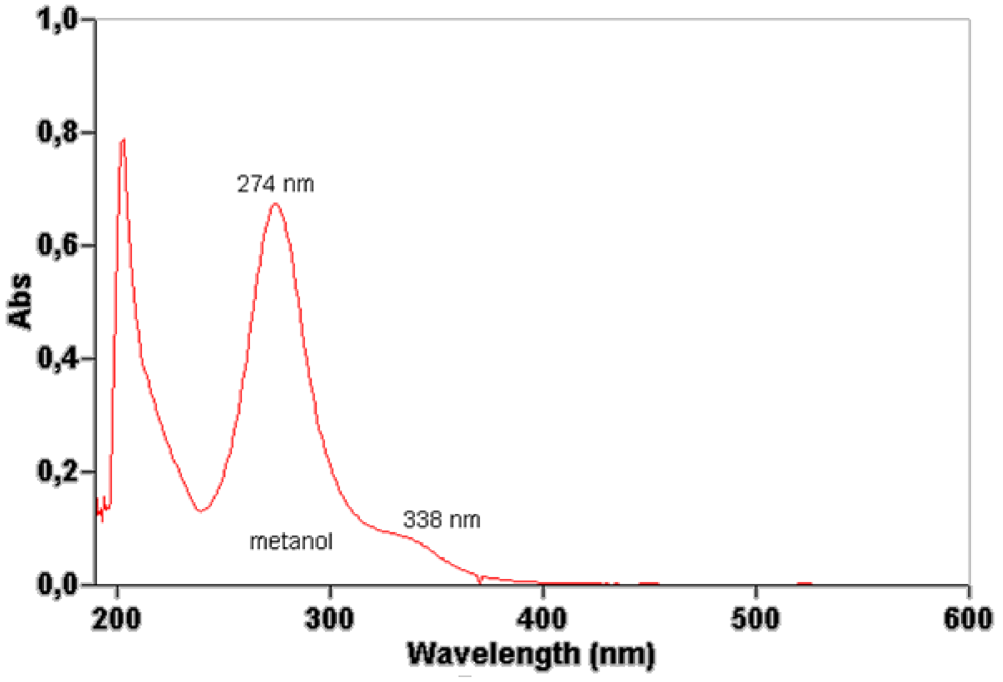

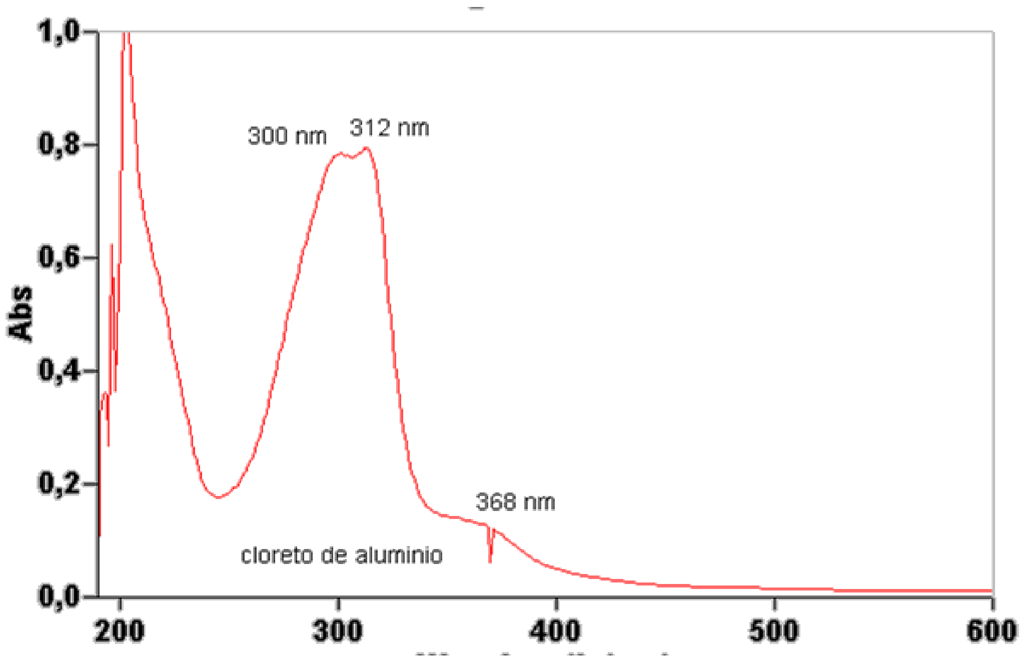

Figuras 44a e 44b: espectro no ultravioleta ($\lambda_{max.}$, MeOH; MeOH + $AlCl_3$) de Pmt-2

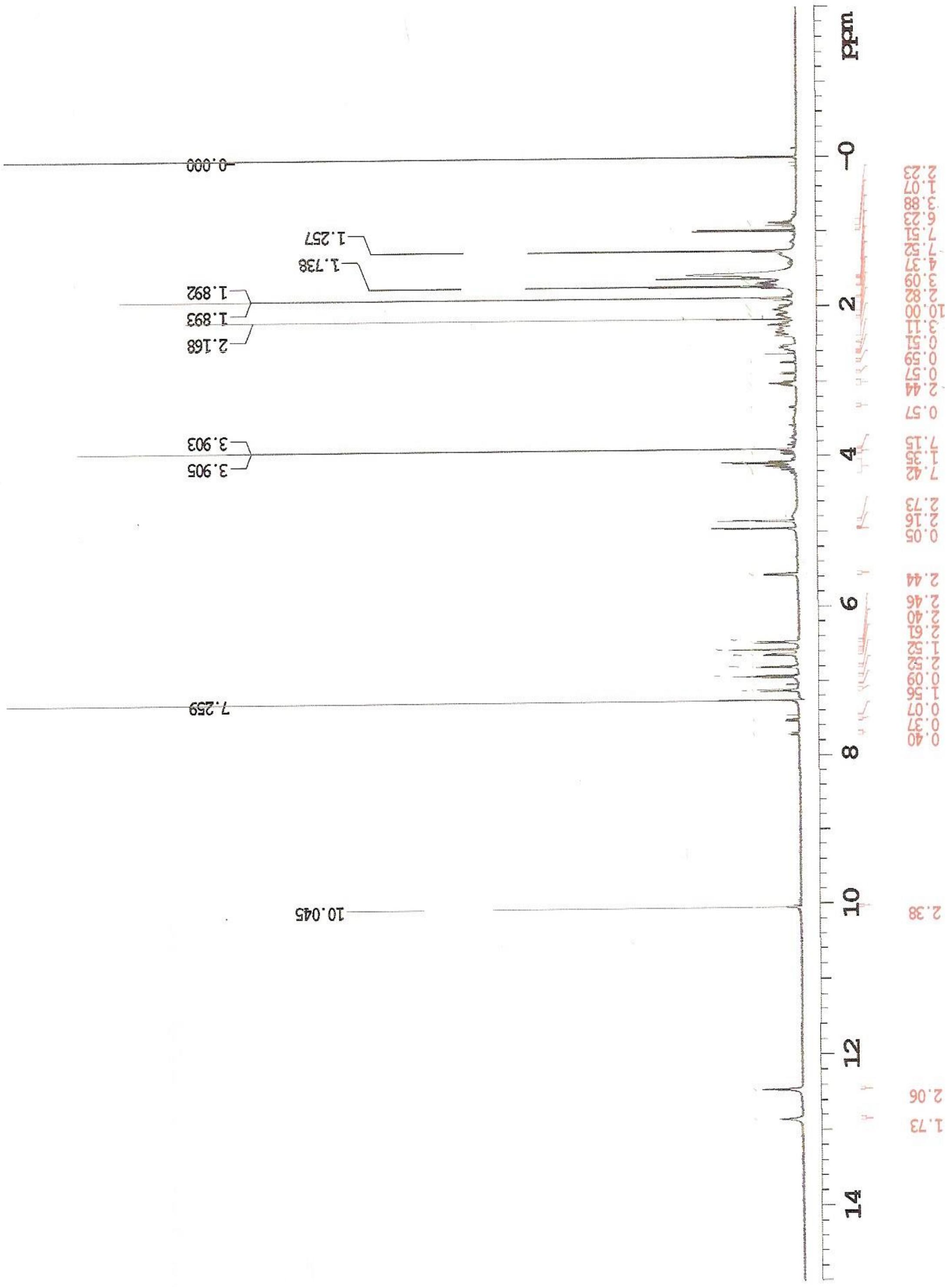

Figura 45: espectro de RMN de ^{1}H (δ, 500 MHz, $CDCl_3$) de Pmt-2

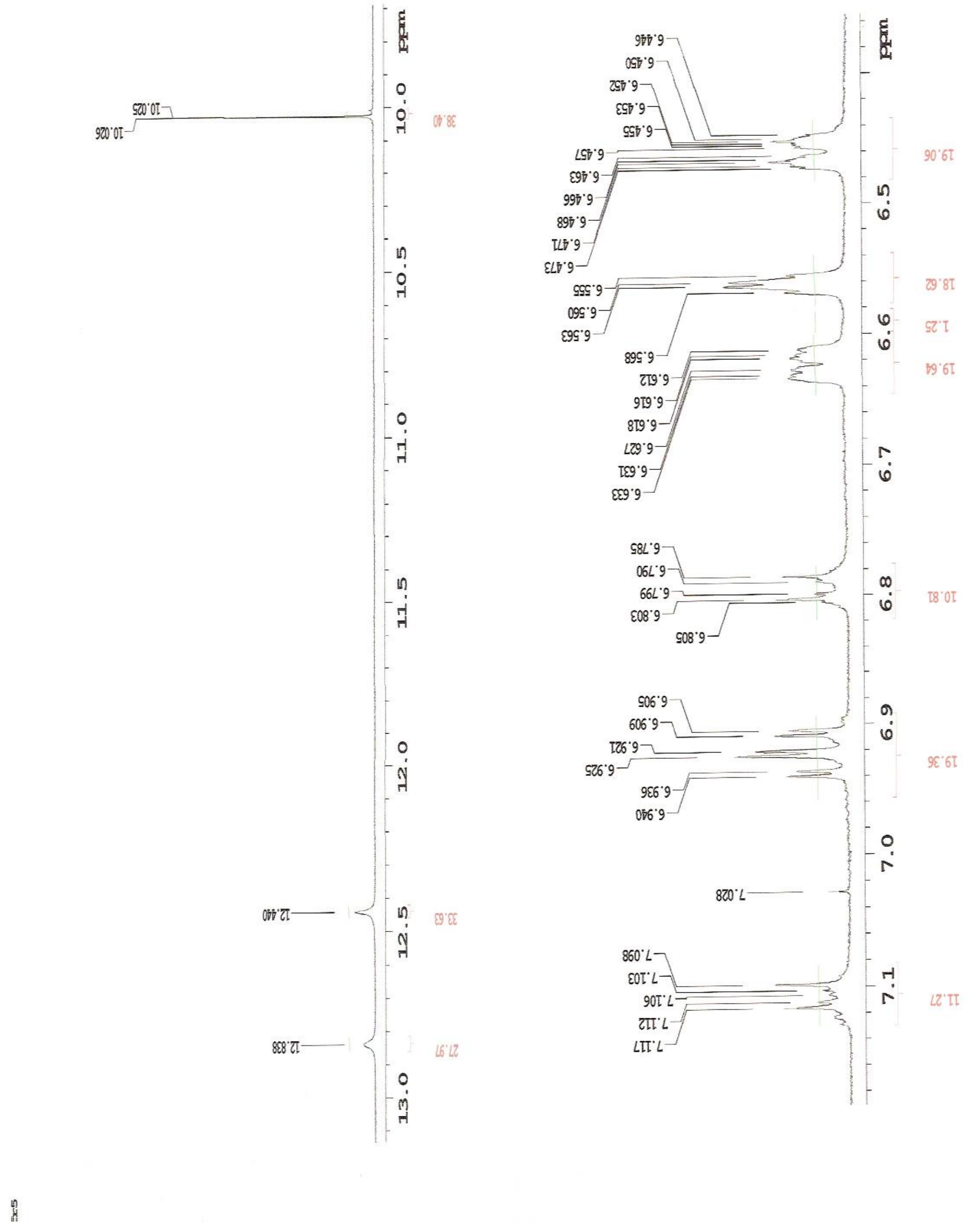

Figura 46 e 47: expansões do espectro de RMN de ^1H (δ, 500 MHz, $CDCl_3$) de Pmt-2

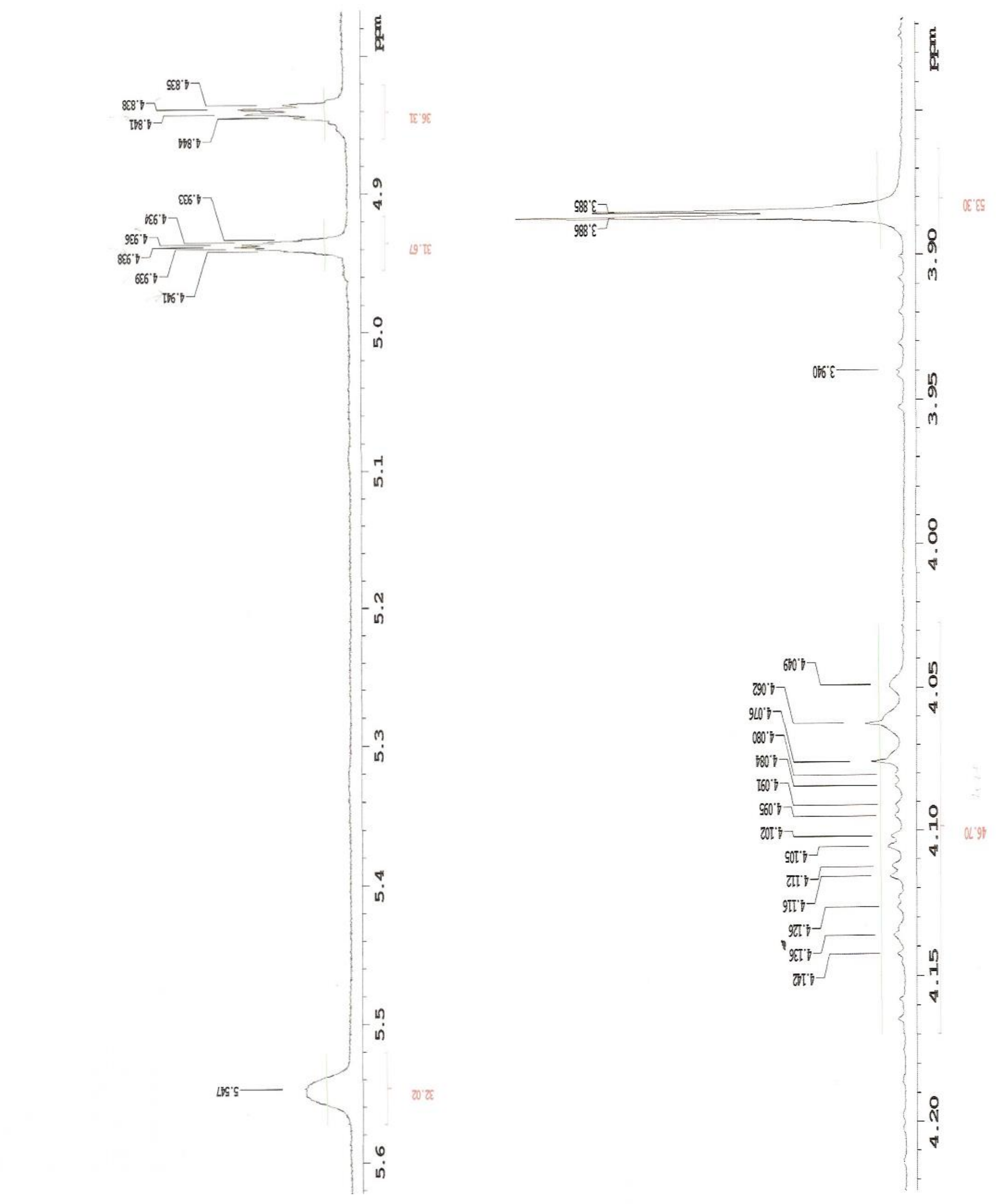

Figura 48 e 49: expansões do espectro de RMN de ^{1}H (δ, 500 MHz, $CDCl_3$) de Pmt-2

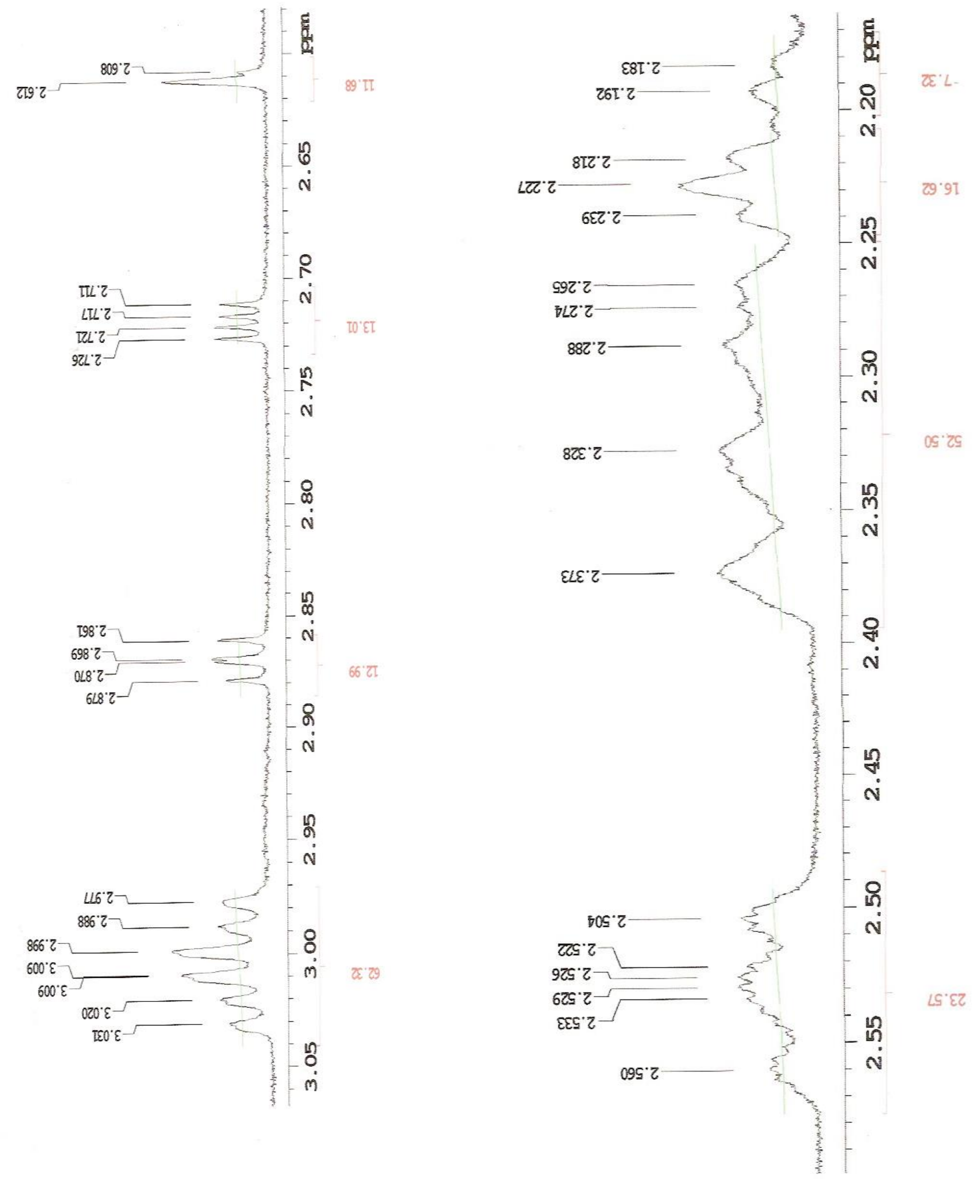

Figura 50 e 51: expansões do espectro de RMN de ^{1}H (δ, 500 MHz, $CDCl_3$) de Pmt-2

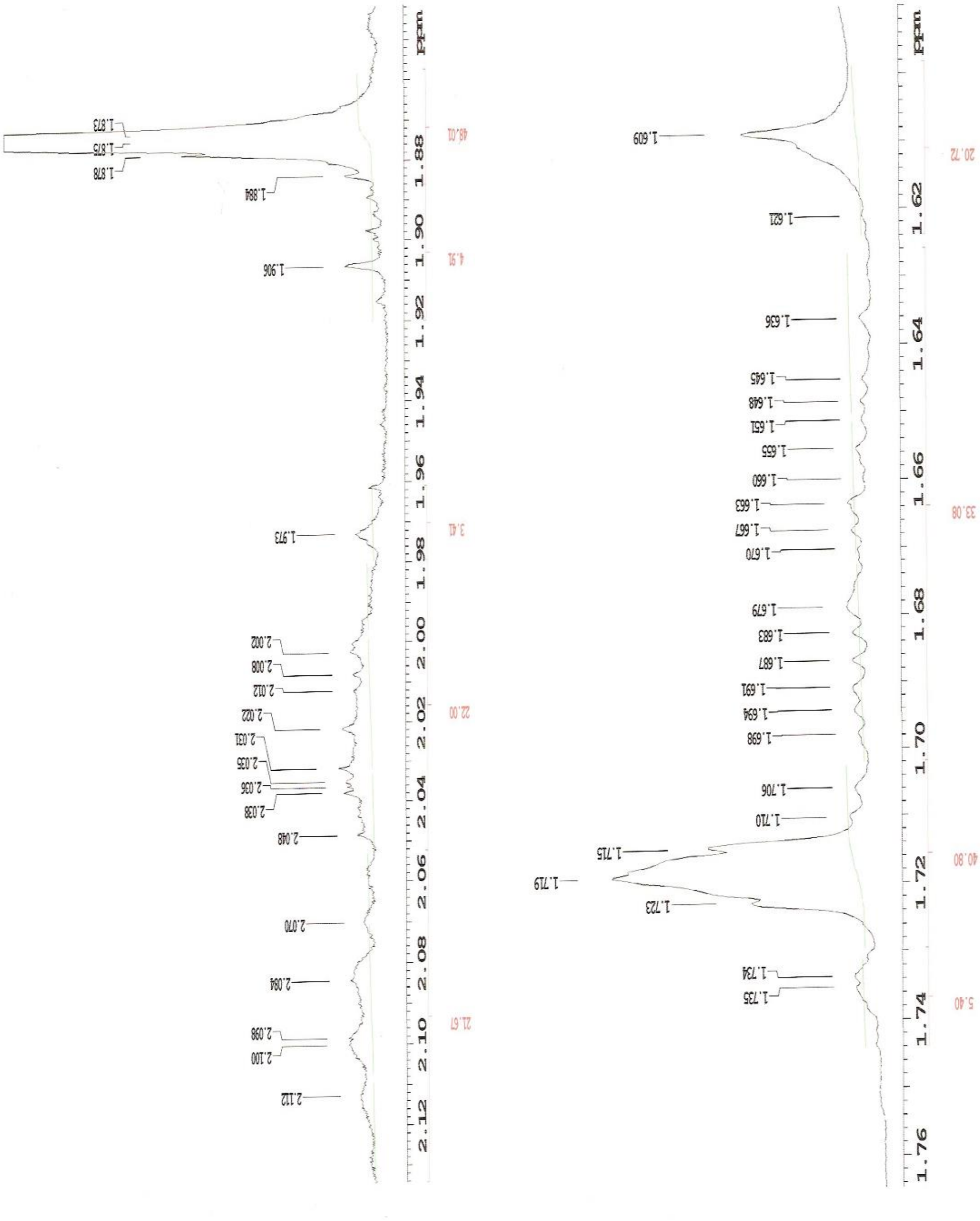

Figura 52 e 53: expansões do espectro de RMN de ^{1}H (δ, 500 MHz, $CDCl_3$) de Pmt-2

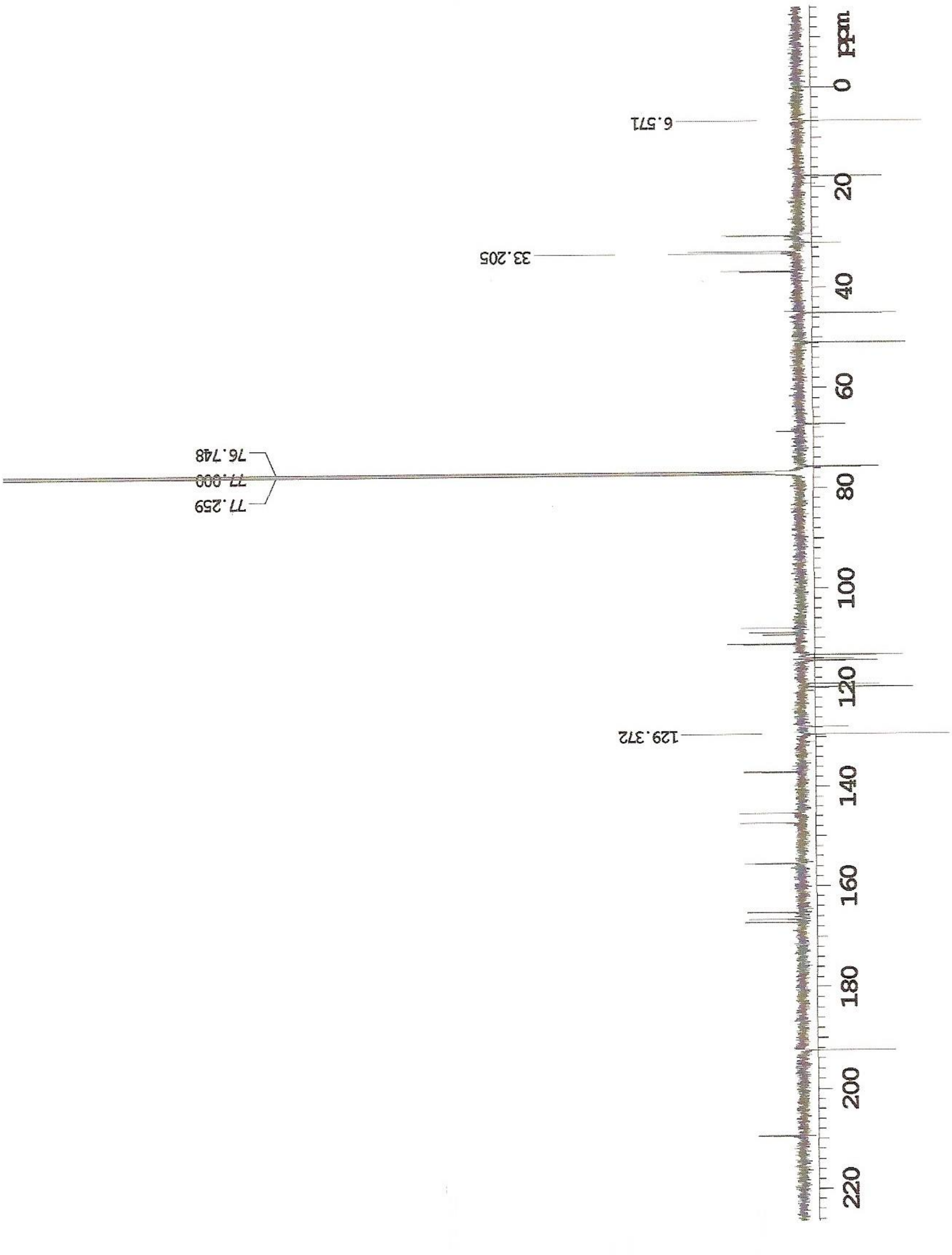

Figura 54: espectro de RMN de ^{13}C (δ, 500 MHz, $CDCl_3$) de Pmt-2

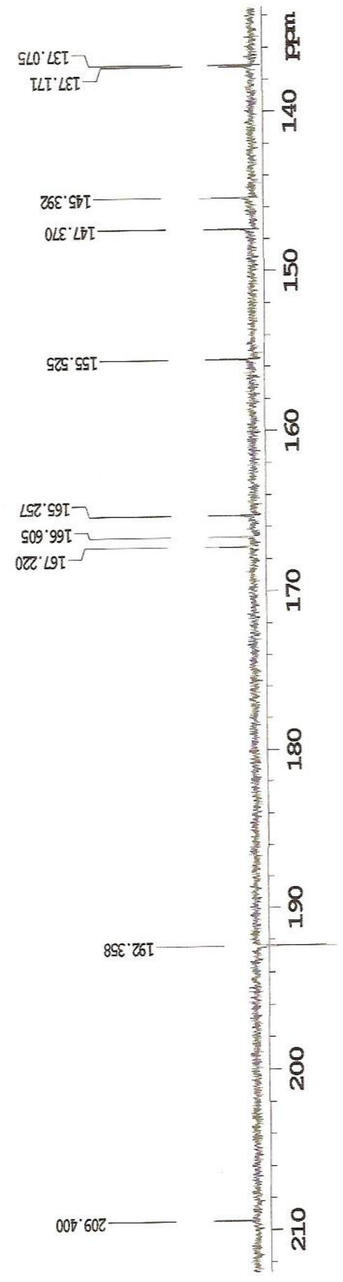

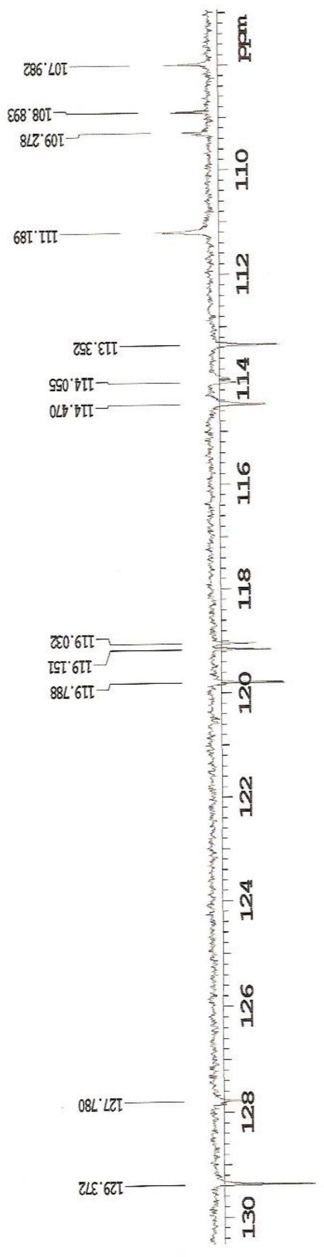

Figura 55 e 56: expansões do espectro de RMN de ^{13}C (δ, 500 MHz, $CDCl_3$) de Pmt-2

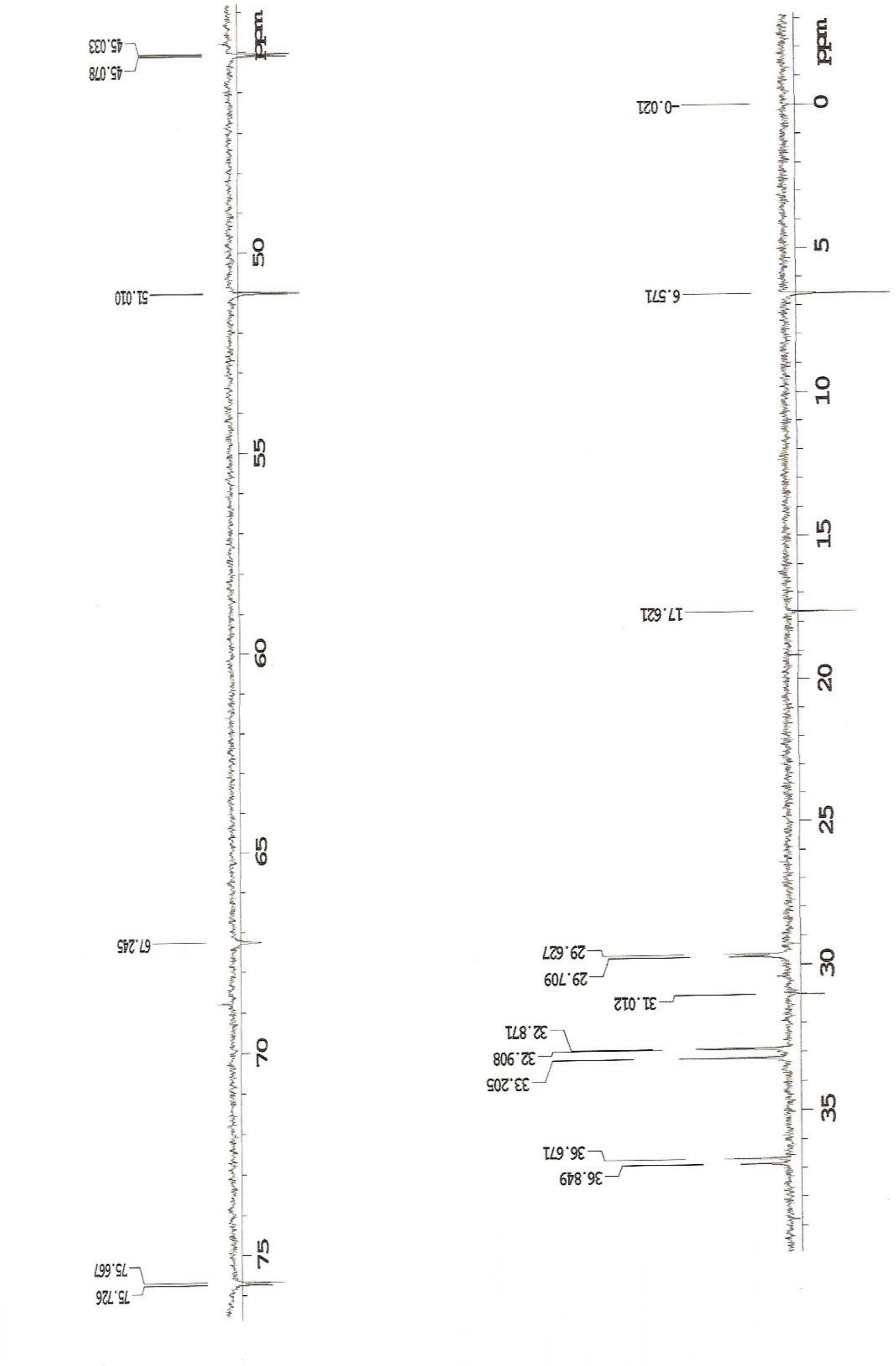

Figura 57 e 58: expansões do espectro de RMN de ^{13}C (δ, 500 MHz, $CDCl_3$) de Pmt-2

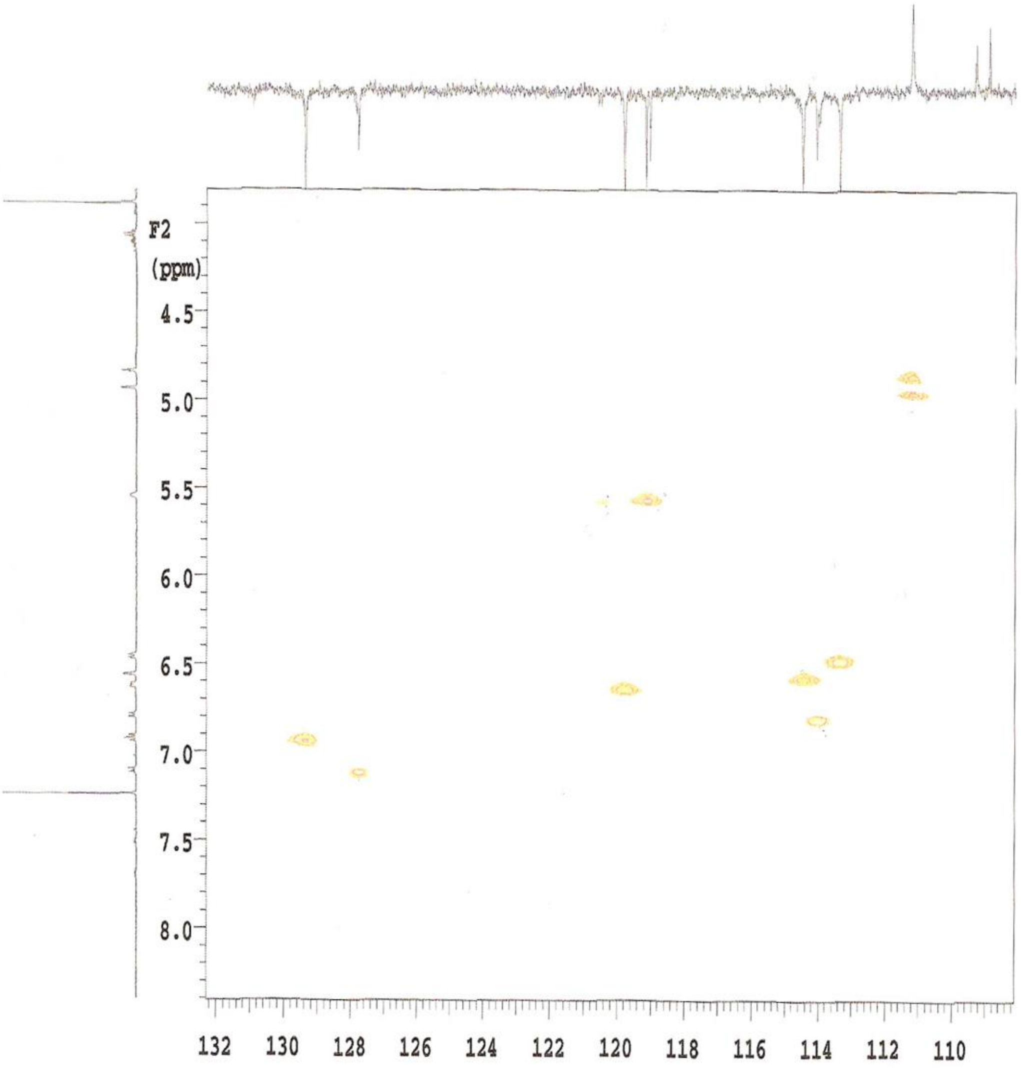

Figura 59: expansão do espectro de correlação heteronuclear HMQC – ^{1}H x ^{13}C de Pmt-2

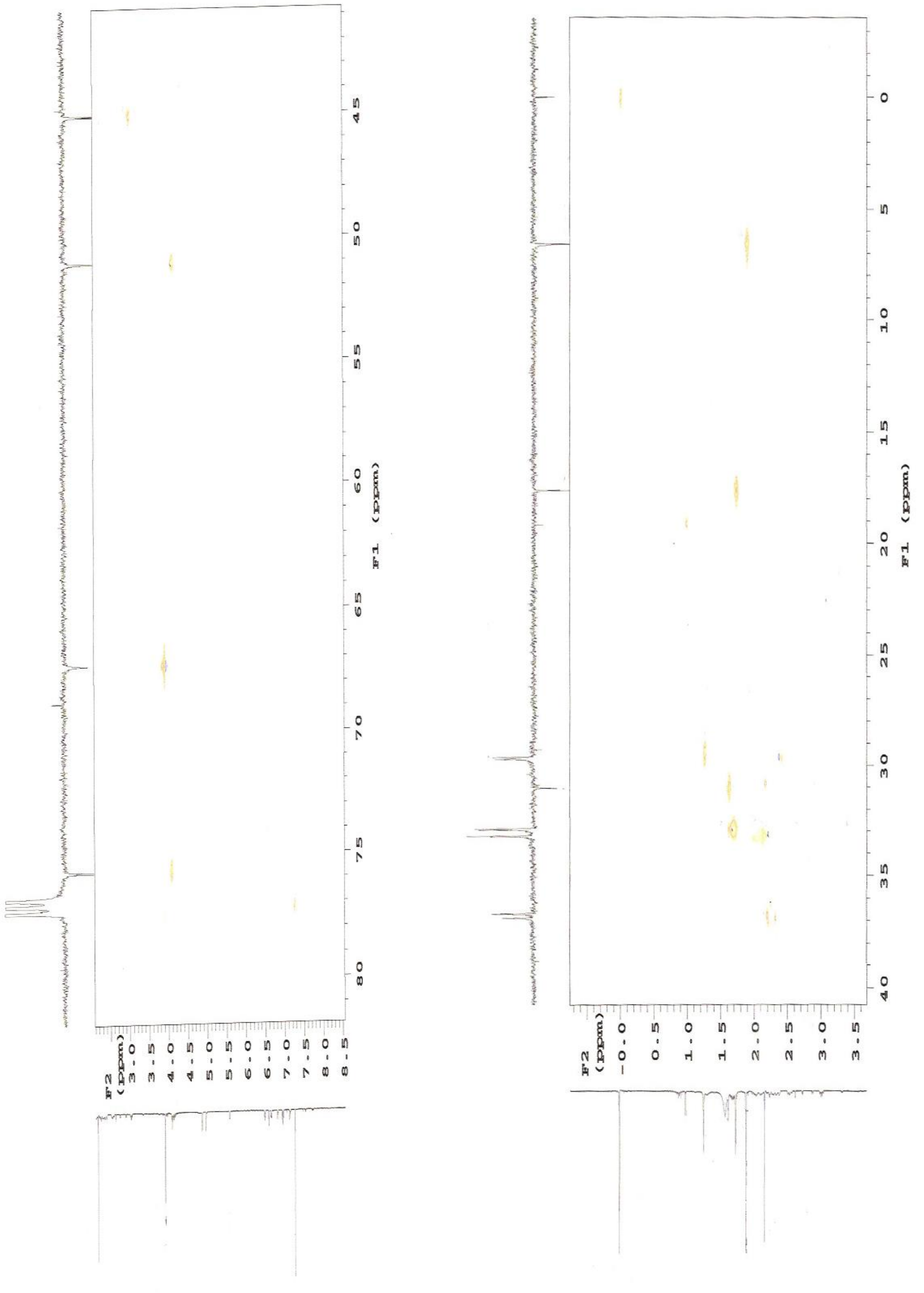

Figura 60 e 61: expansões do espectro de correlação heteronuclear HMQC – ^{1}H x ^{13}C de Pmt-2

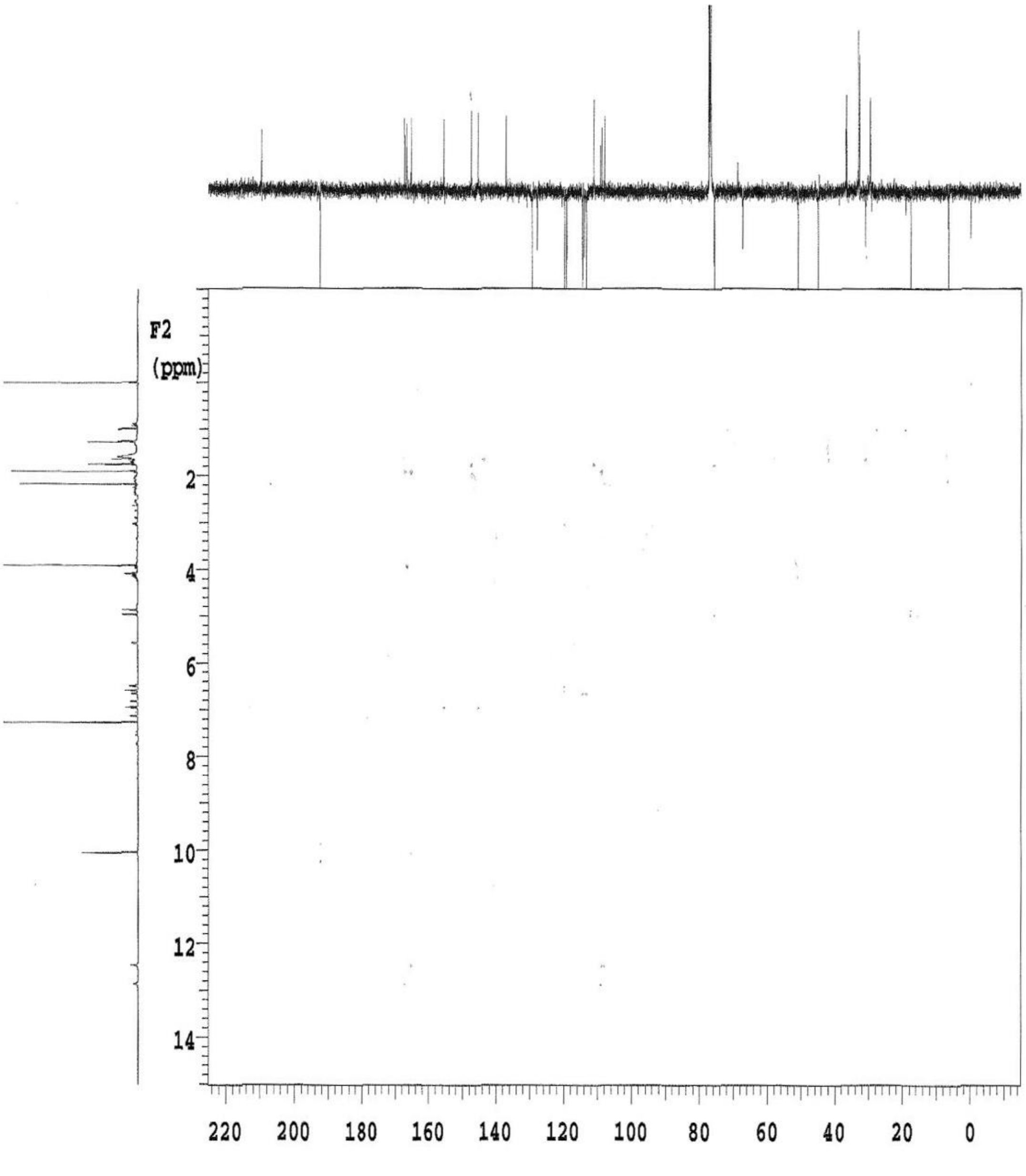

Figura 62: espectro de correlação heteronuclear HMBC – ^{1}H x ^{13}C de Pmt-2

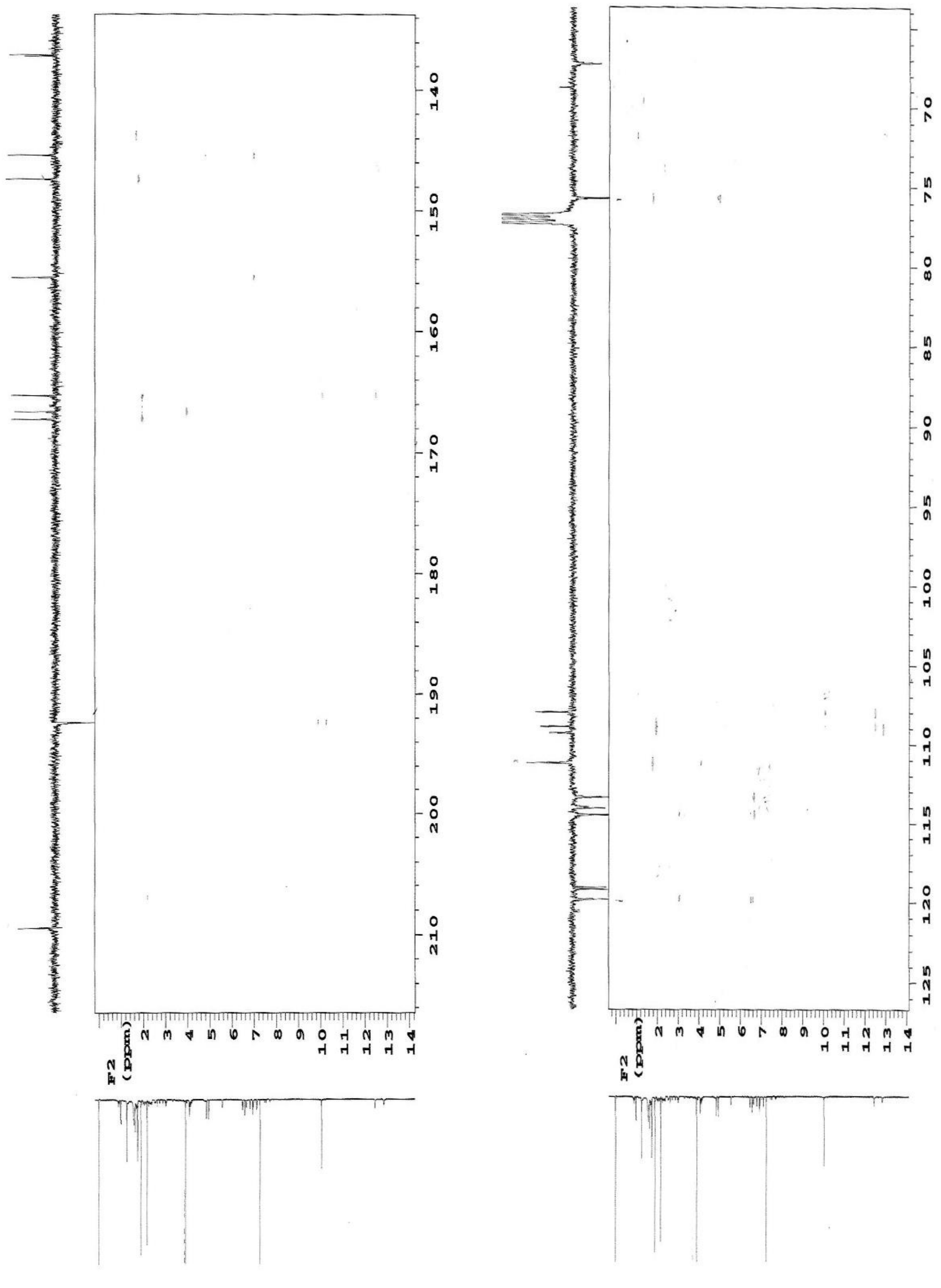

Figura 63 e 64: espectro de correlação heteronuclear HMBC – ^{1}H x ^{13}C de Pmt-2

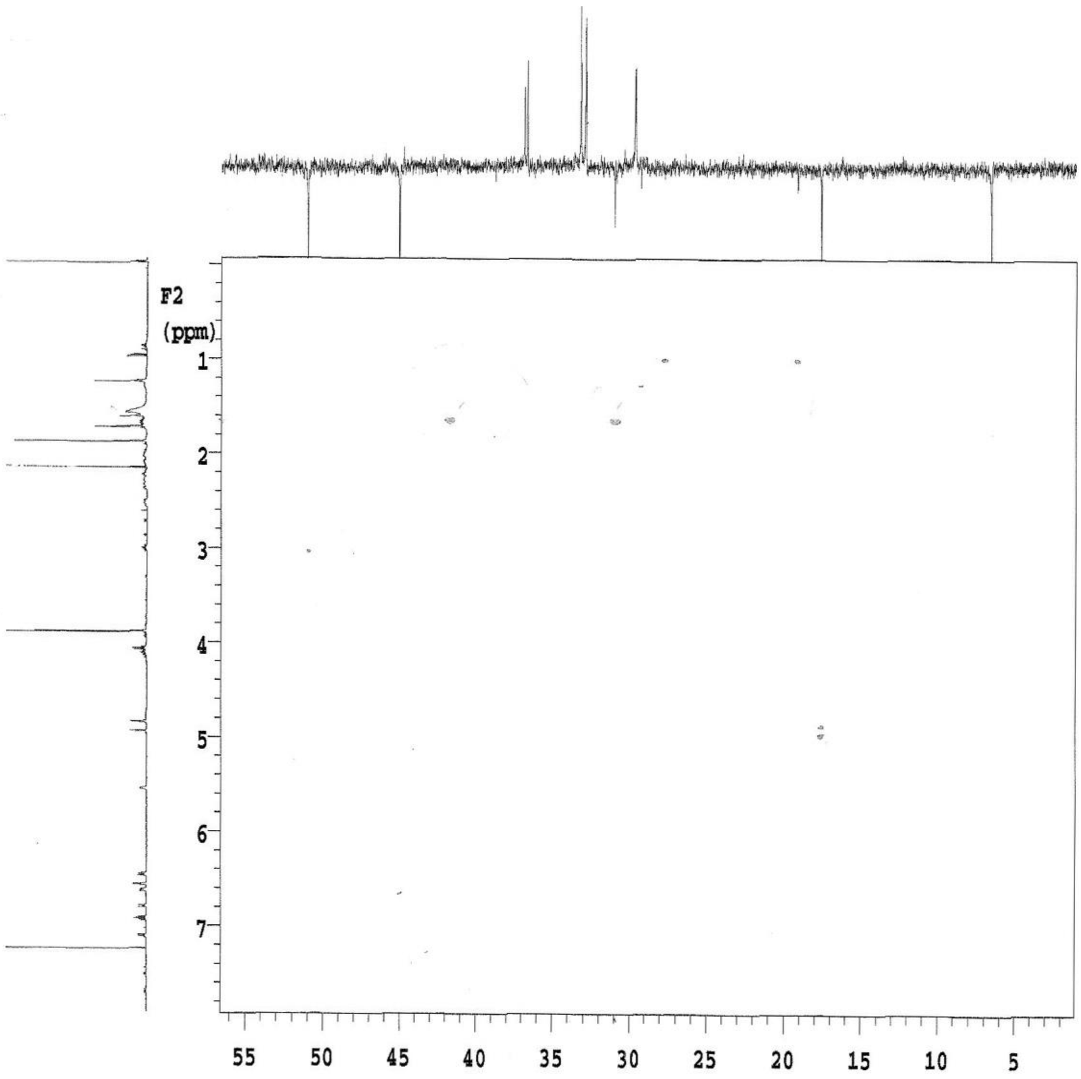

Figura 65: expansão do espectro de correlação heteronuclear HMBC – ^{1}H x ^{13}C de Pmt-2

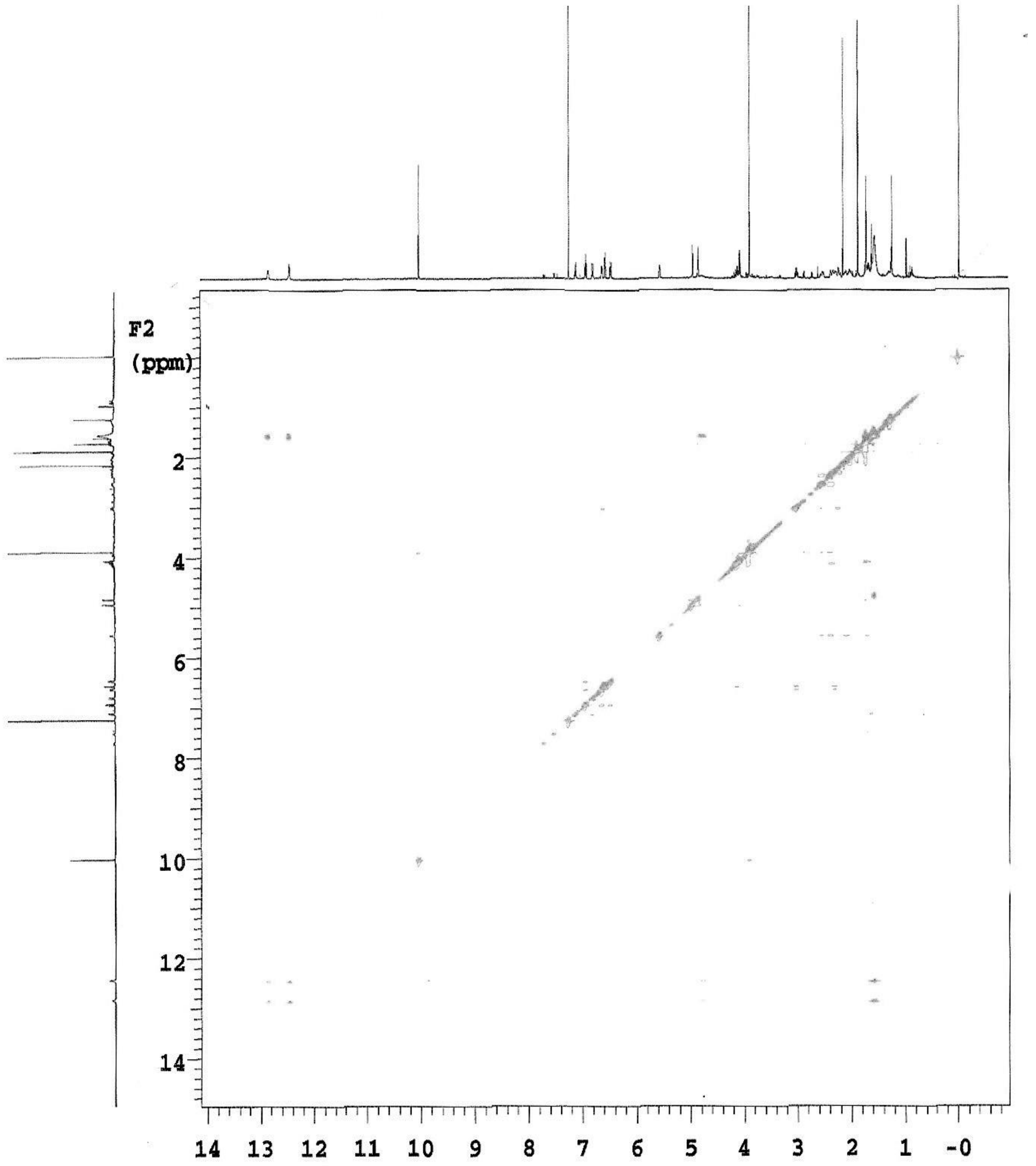

Figura 66: espectro de correlação homonuclear NOESY – ^{1}H x ^{1}H de Pmt-2

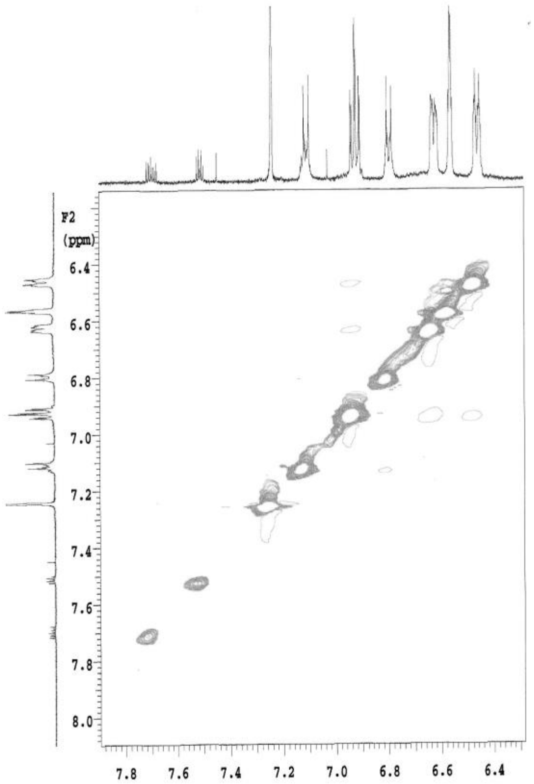

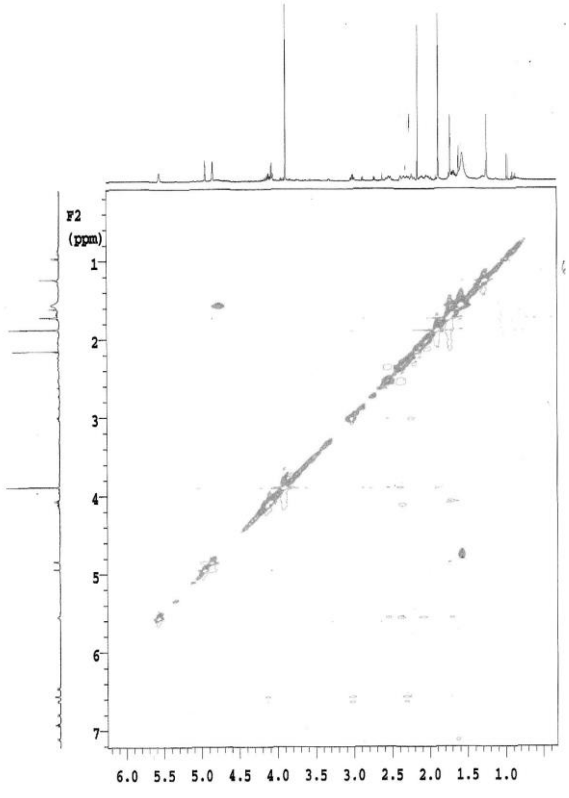

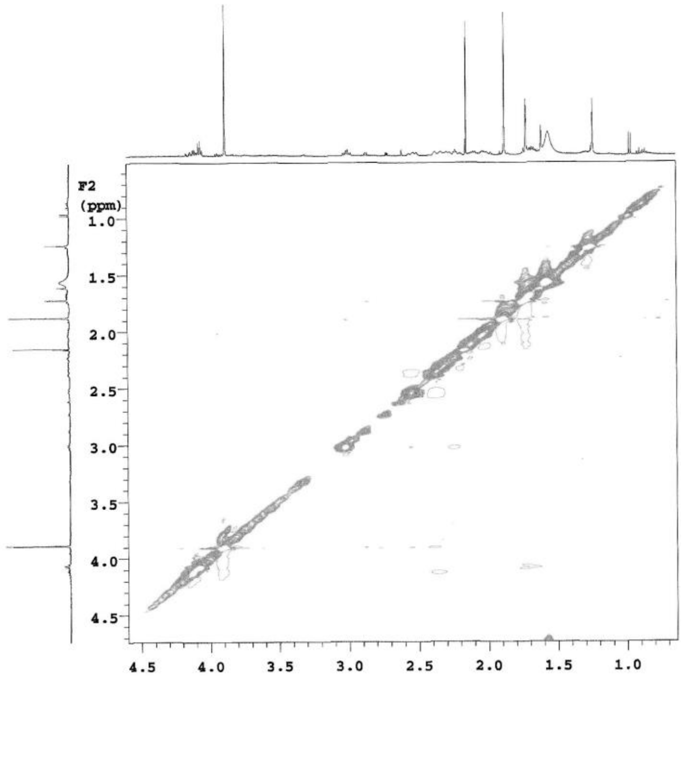

Figura 67, 68 e 69: expansões do espectro de correlação homonuclear NOESY – ^{1}H x ^{1}H de Pmt-2

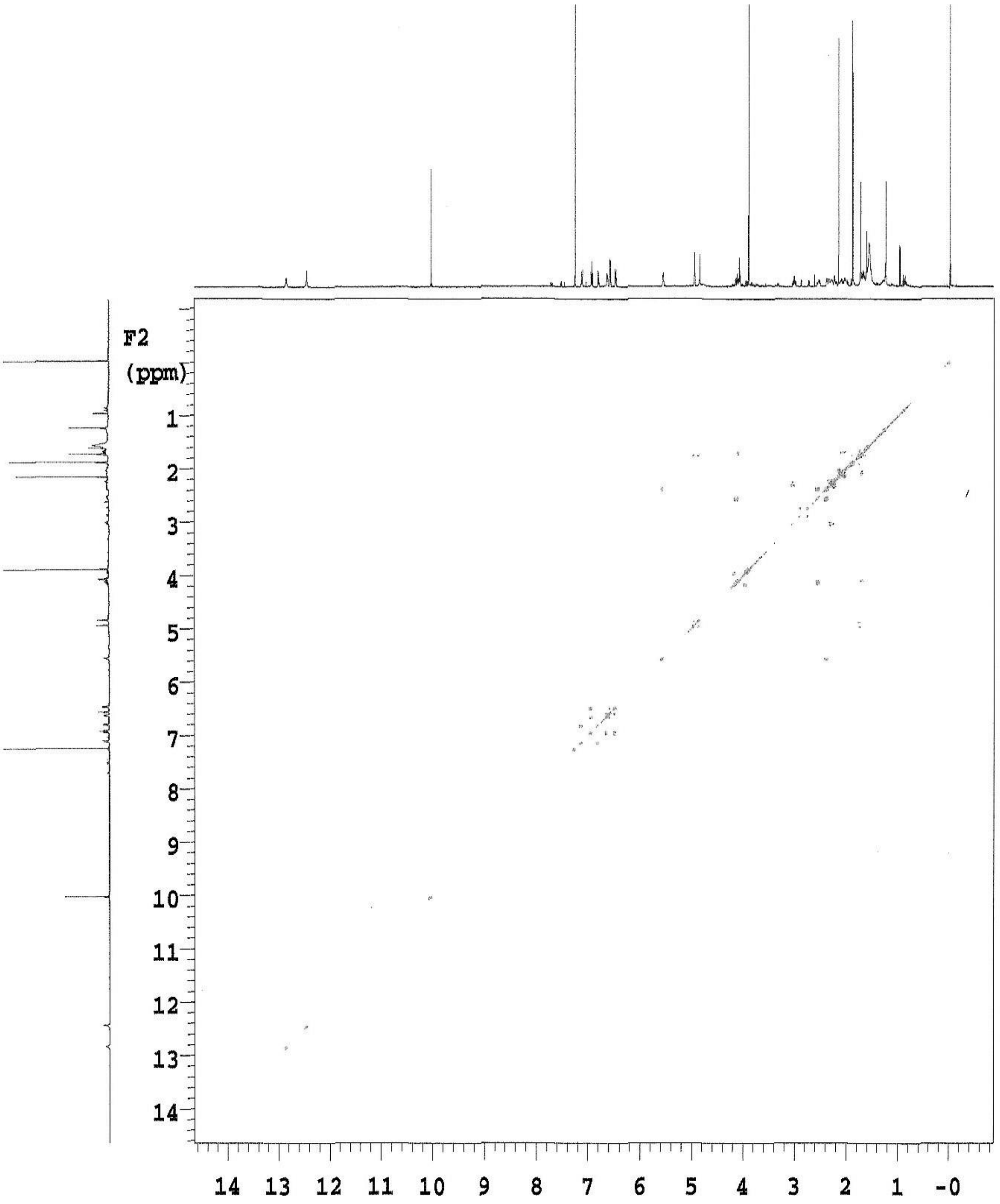

Figura 70: espectro de correlação homonuclear COSY – ^{1}H x ^{1}H de Pmt-2

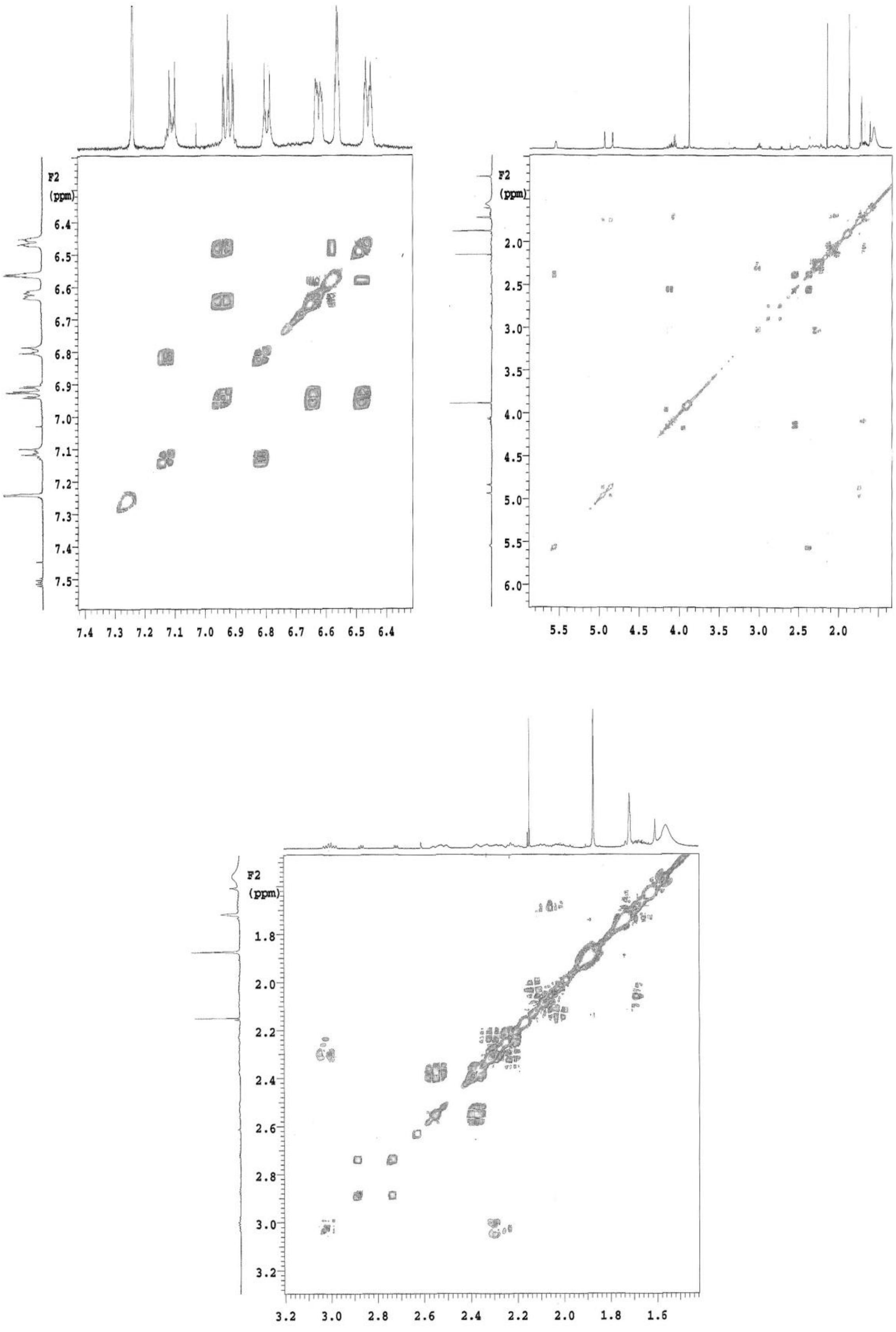

Figura 71, 72 e 73: expansões do espectro de correlação homonuclear COSY – ^{1}H x ^{1}H de Pmt-2

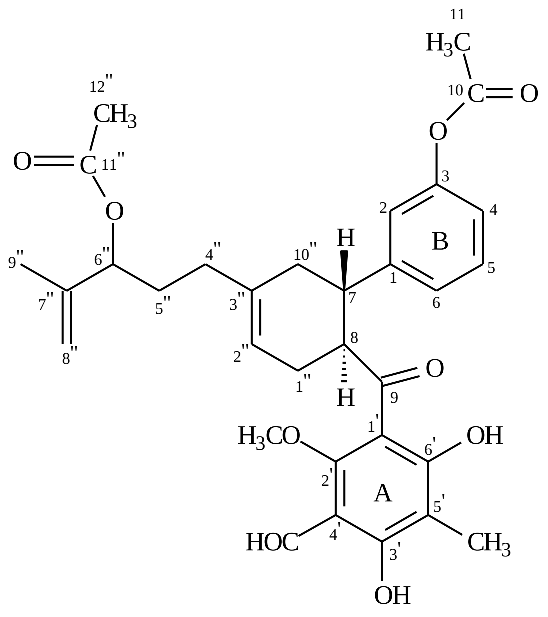

Pmt-2 ACETILADO

Harley
Op-Vicente

Sample: Pmonte-C2.10.4.2-fr5acet.
File: xp

Pulse Sequence: s2pul

Solvent: cdcl3
Temp. 27.0 C / 300.1 K
Operator: vnmr1
VNMRS-500 "varian500.ltf.ufpb.br"

Relax. delay 0.847 sec
Pulse 45.0 degrees
Acq. time 4.153 sec
Width 8012.8 Hz
8 repetitions
OBSERVE H1, 499.5804995 MHz
DATA PROCESSING
Resol. enhancement -0.0 Hz
FT size 131072
Total time 0 min, 50 sec

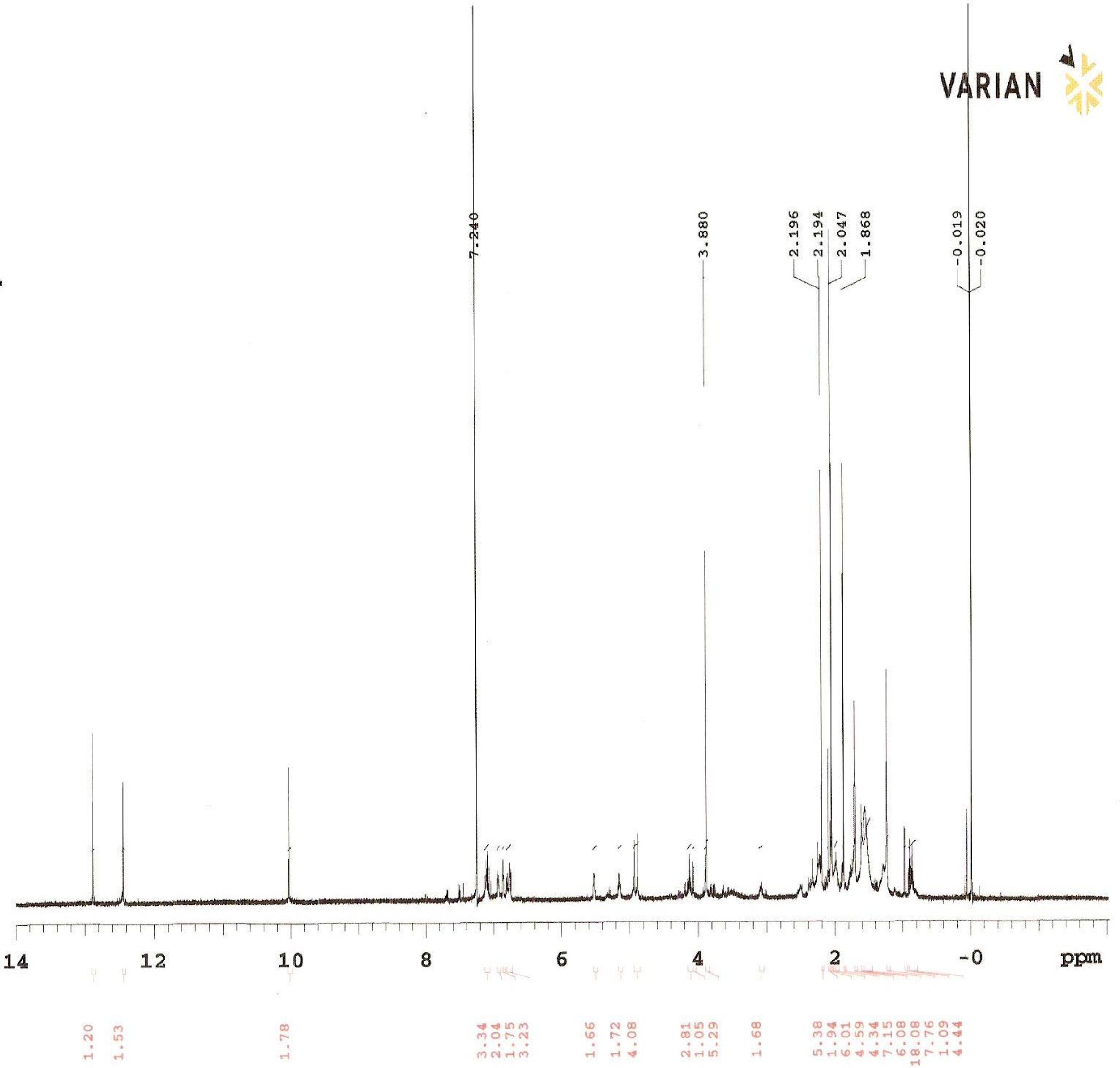

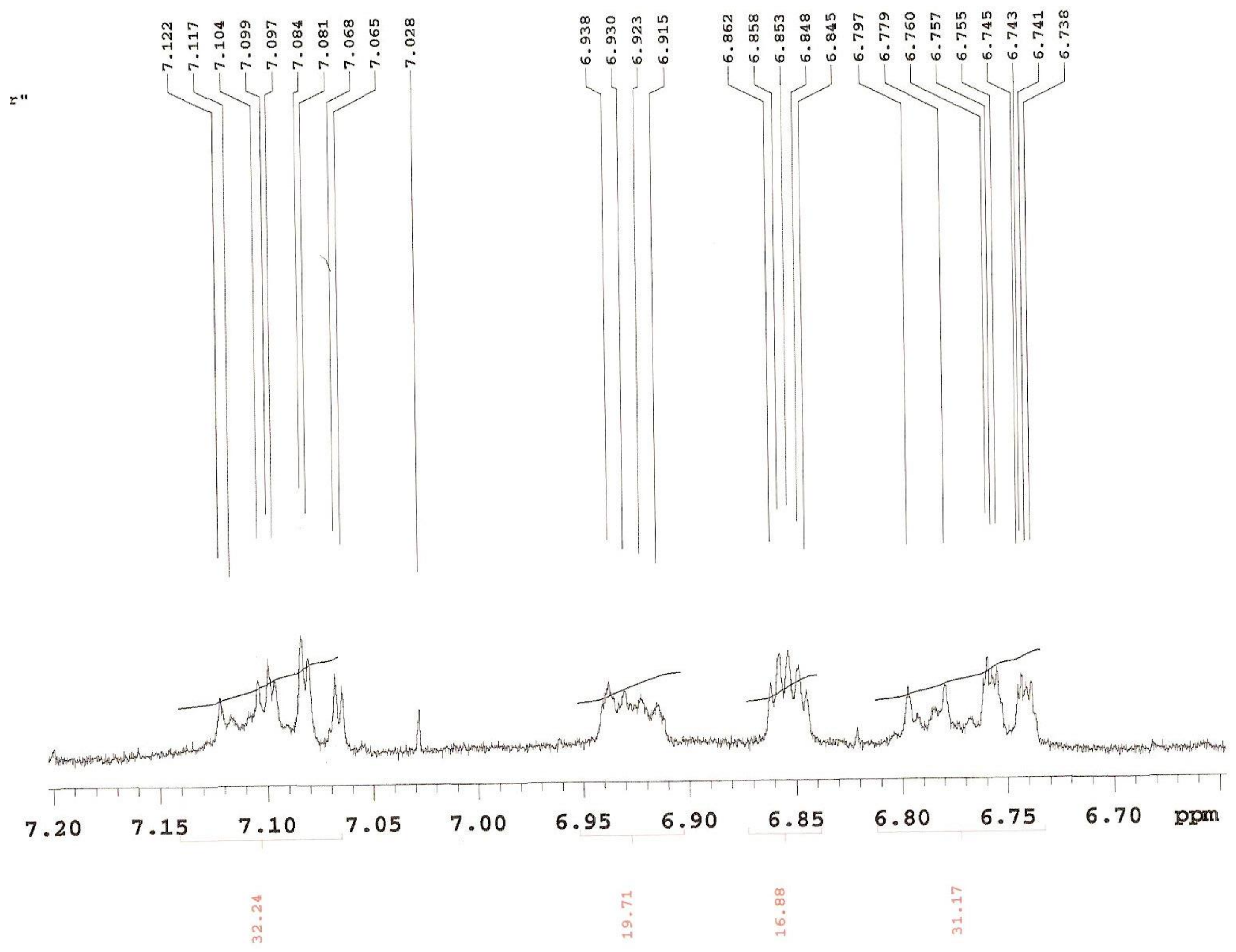

7.122
7.117
7.104
7.099
7.097
7.084
7.081
7.068
7.065
7.028
6.938
6.930
6.923
6.915
6.862
6.858
6.853
6.848
6.845
6.797
6.779
6.760
6.757
6.755
6.745
6.743
6.741
6.738
7.20
7.15
7.10
7.05
7.00
6.95
6.90
6.85
6.80
6.75
6.70
ppm
32.24
19.71
16.88
31.17

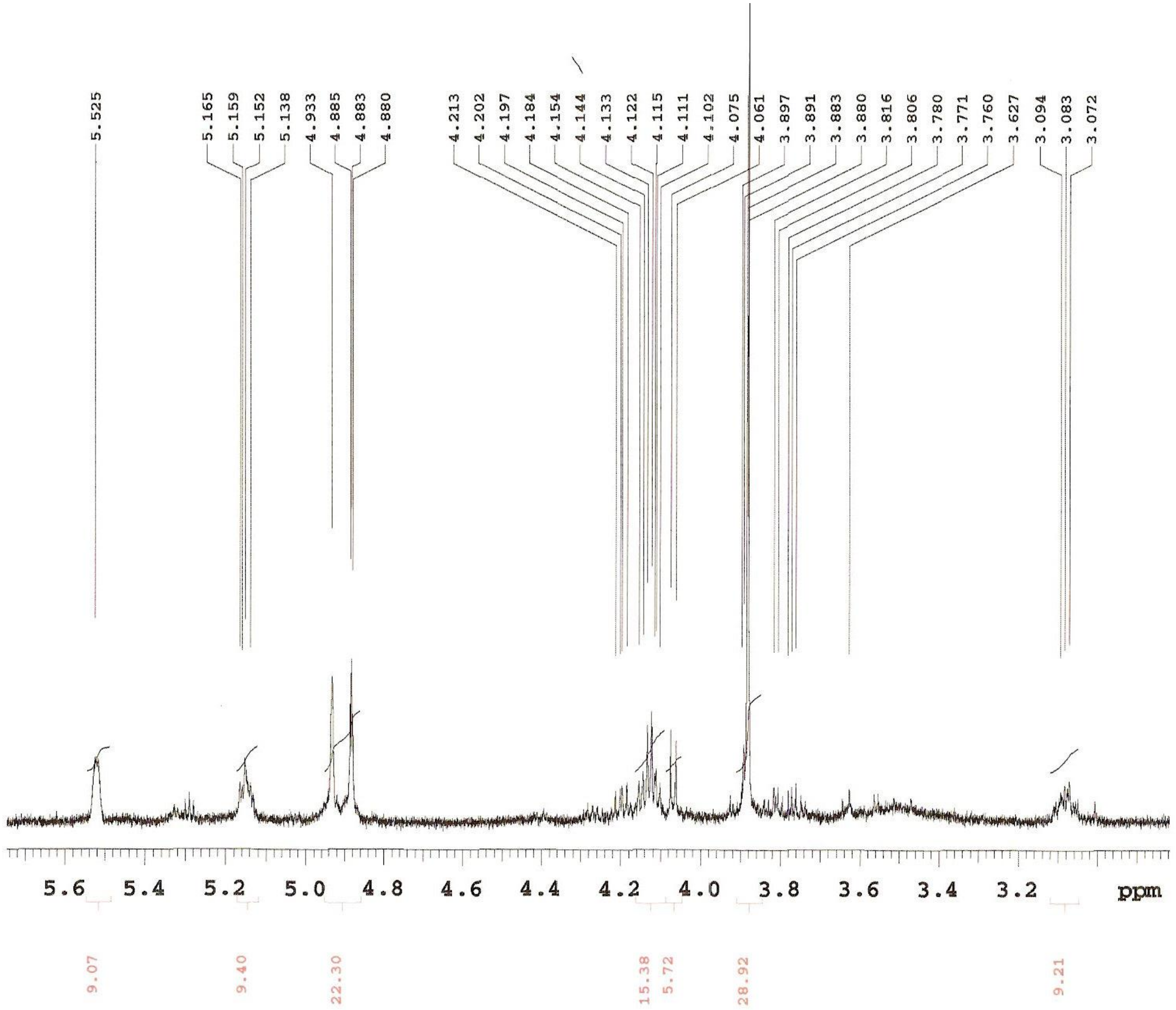

3.072
3.083
3.094
3.627
3.760
3.771
3.780
3.806
3.816
3.880
3.883
3.891
3.897
4.061
4.075
4.102
4.111
4.115
4.122
4.133
4.144
4.154
4.184
4.197
4.202
4.213
4.880
4.883
4.885
4.933
5.138
5.152
5.159
5.165
5.525
9.07
9.40
22.30
15.38
5.72
28.92
9.21
5.6
5.4
5.2
5.0
4.8
4.6
4.4
4.2
4.0
3.8
3.6
3.4
3.2
ppm

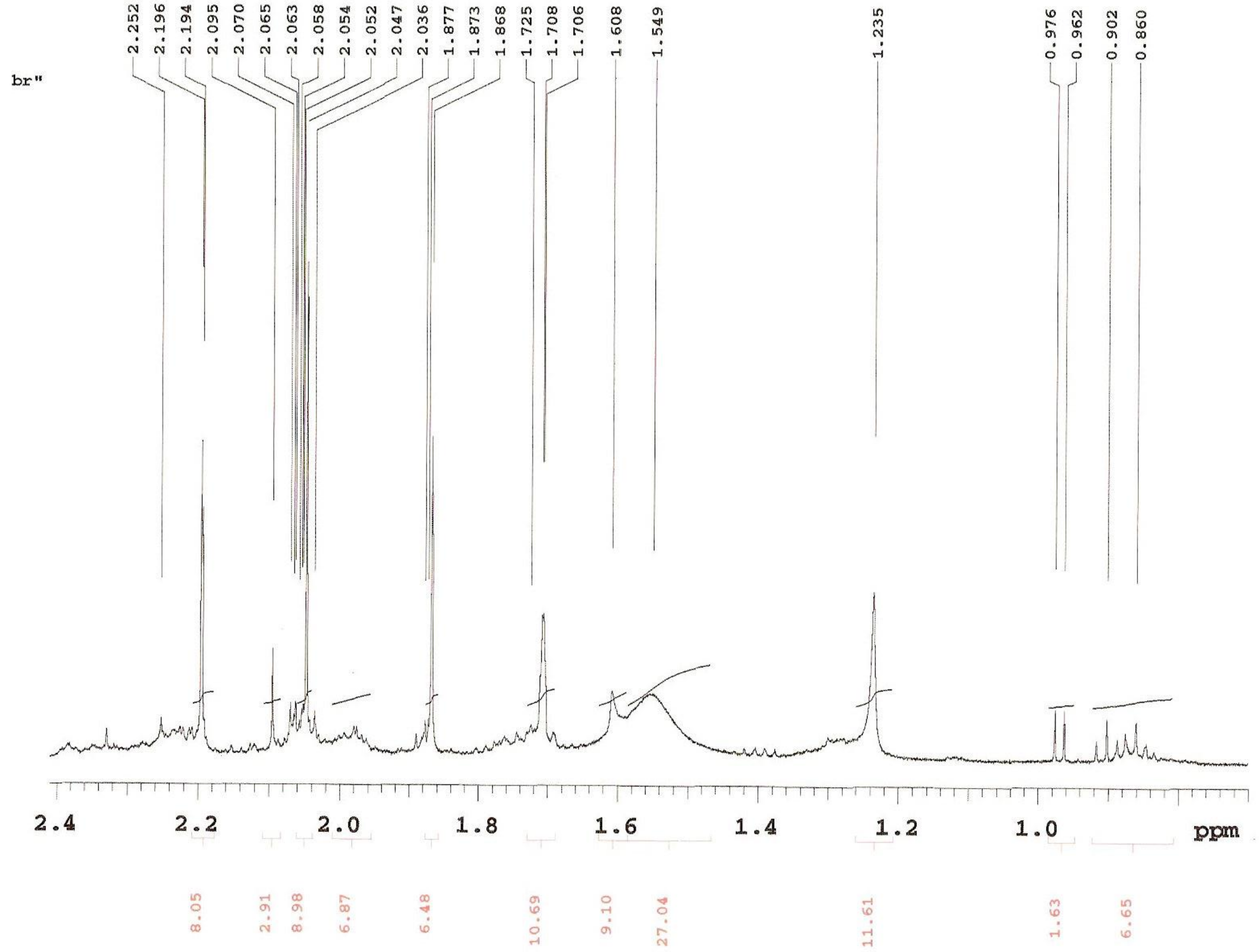
br"
2.252
2.196
2.194
2.095
2.070
2.065
2.063
2.058
2.054
2.052
2.047
2.036
1.877
1.873
1.868
1.725
1.708
1.706
1.608
1.549
1.235
0.976
0.962
0.902
0.860
2.4
2.2
2.0
1.8
1.6
1.4
1.2
1.0
ppm
8.05
2.91
8.98
6.87
6.48
10.69
9.10
27.04
11.61
1.63
6.65

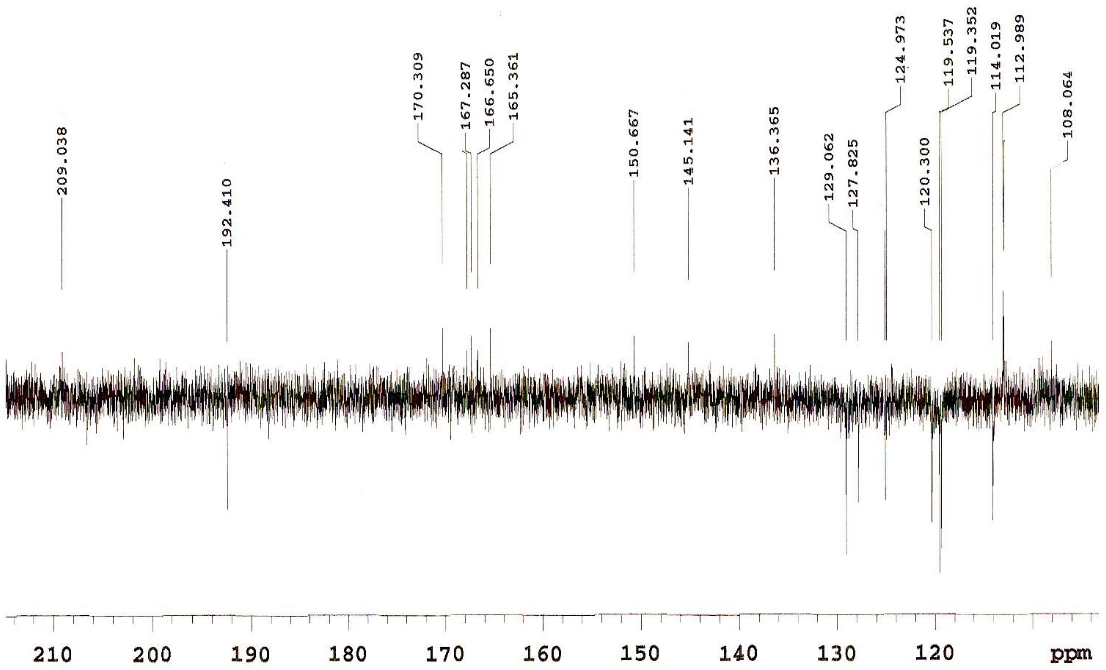

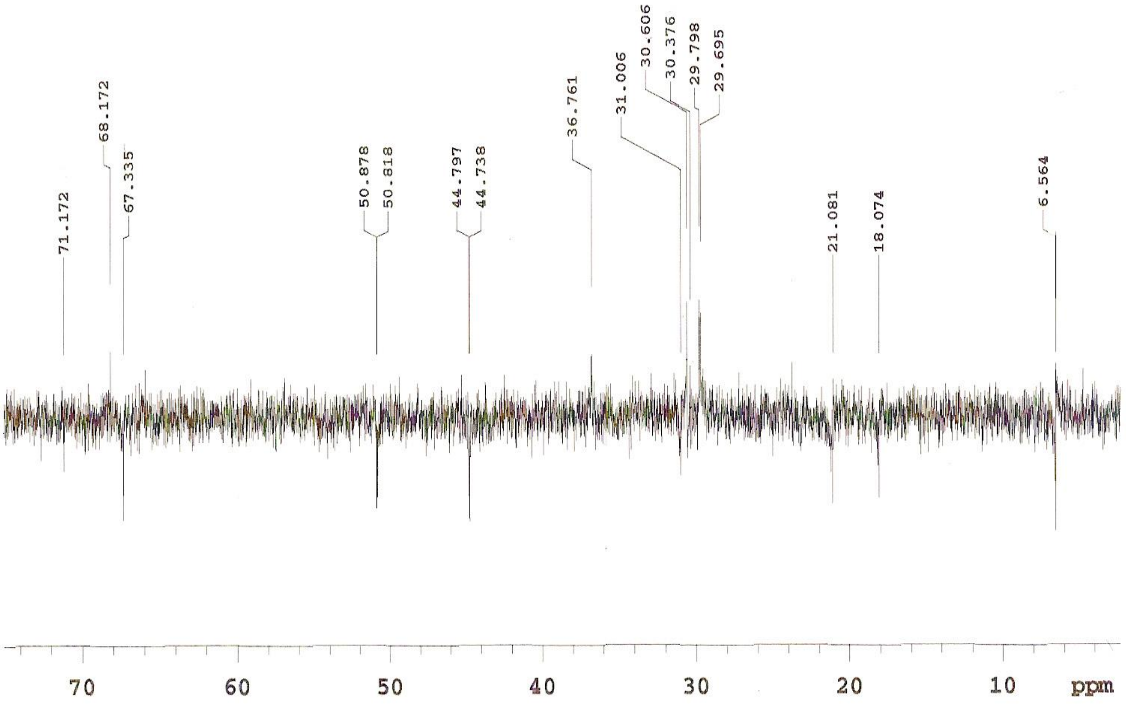

Figura 74 e 75: expansões do espectro de RMN de ^{13}C (δ, 125 MHz, $CDCl_3$) de Pmt-2 acetilado

Espectros de 4

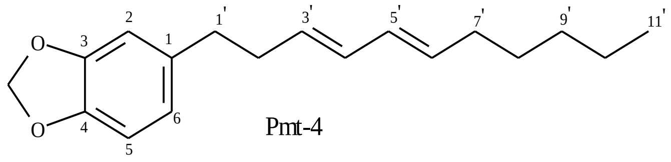

Pmt-4

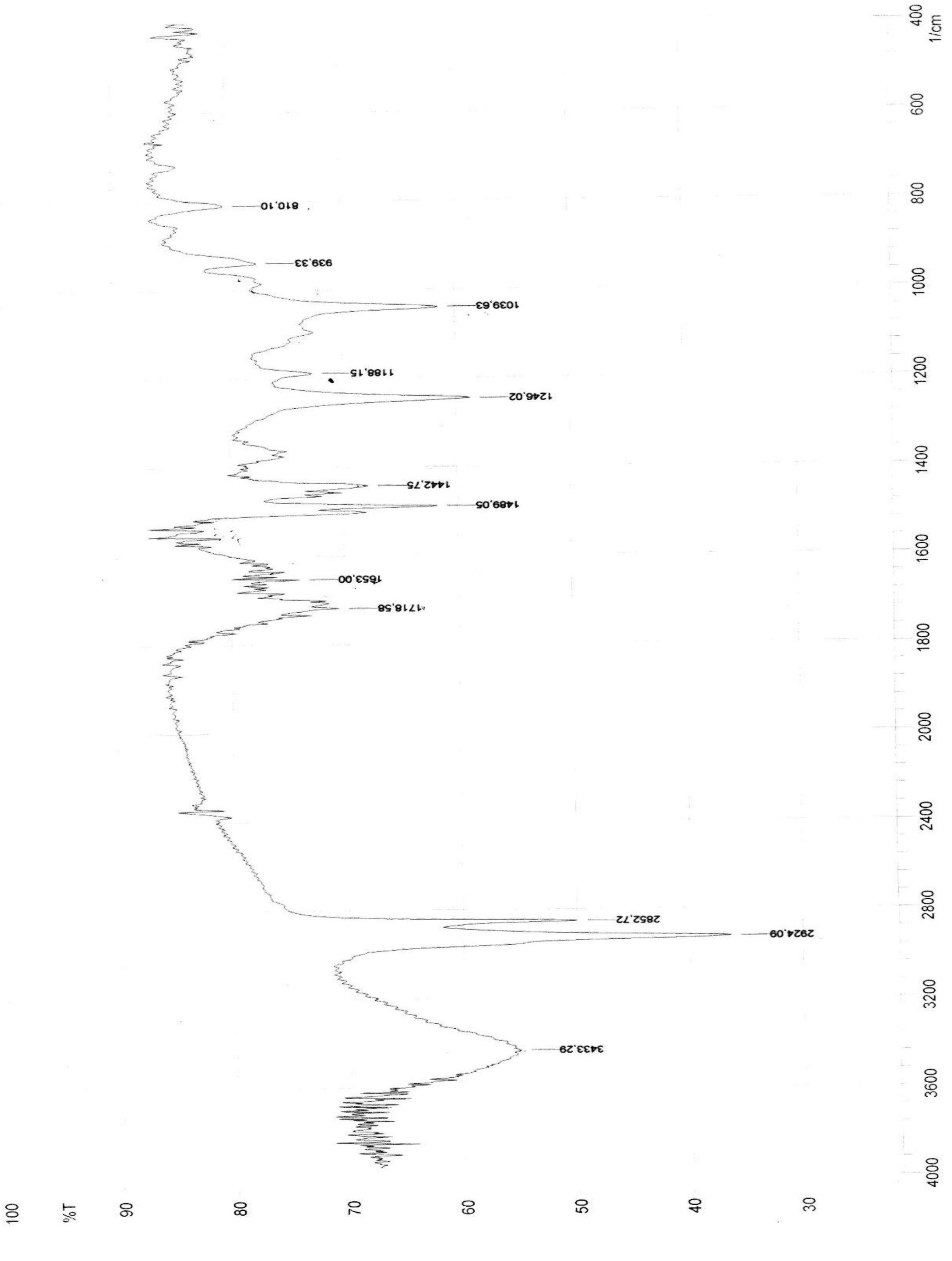

Figura 97: espectro no Infravermelho ($\lambda_{max,}$ KBr, cm^{-1}) de Pmt-4

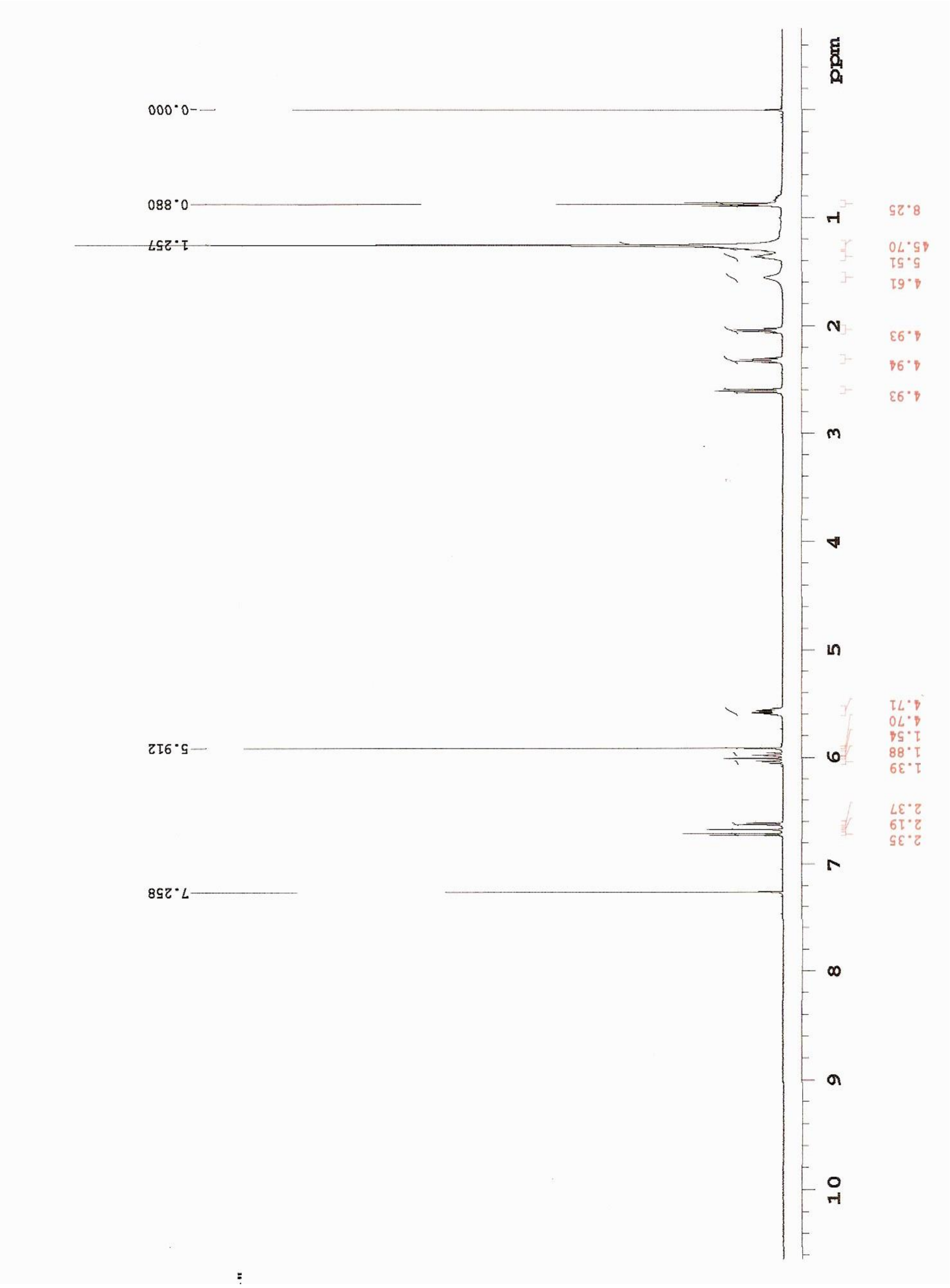

Figura 98: espectro de RMN de ^{1}H (δ, 500 MHz, $CDCl_3$) de Pmt-4

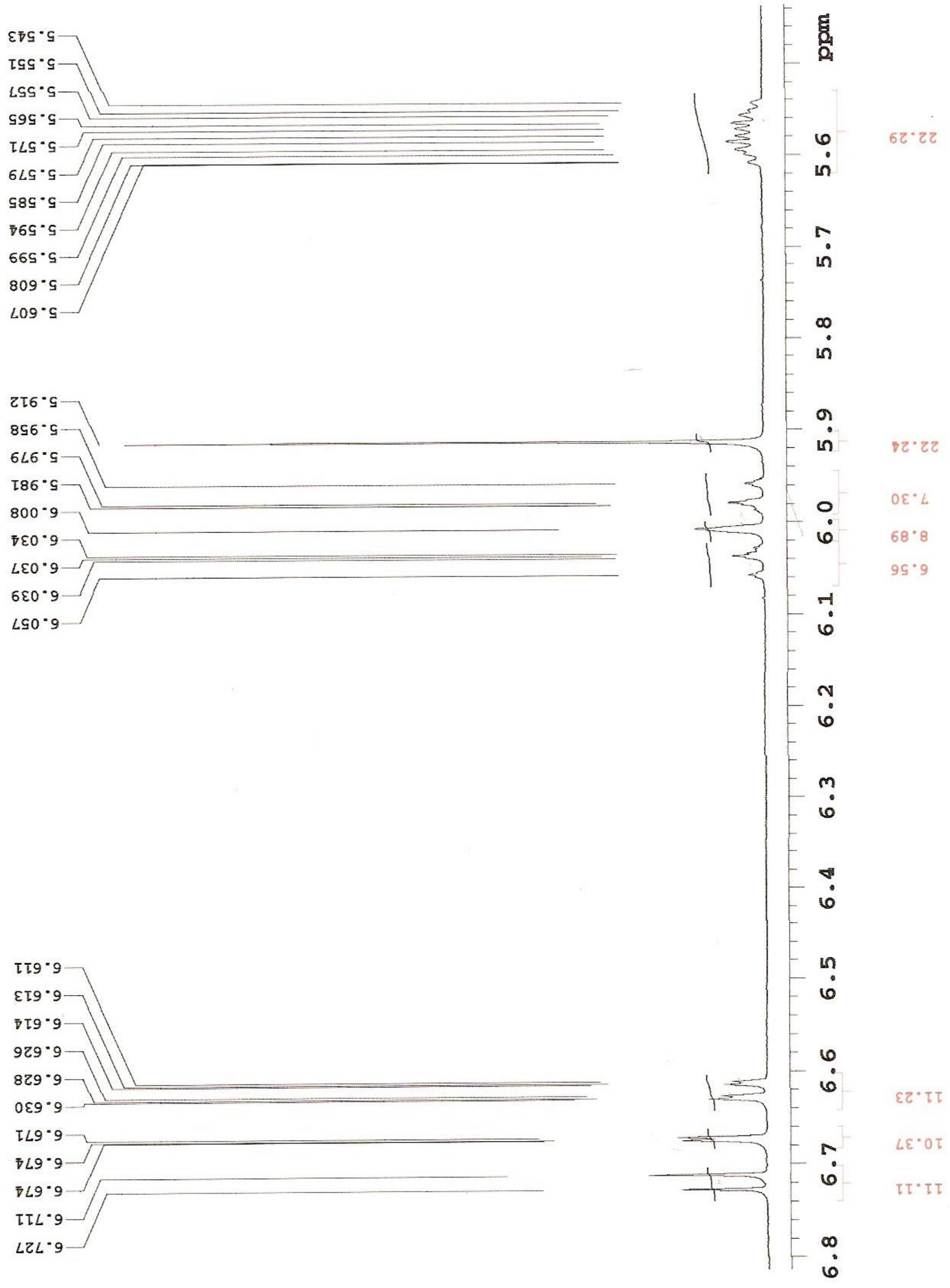

Figura 99: expansão do espectro de RMN de ^{1}H (δ, 500 MHz, $CDCl_3$) de Pmt-4

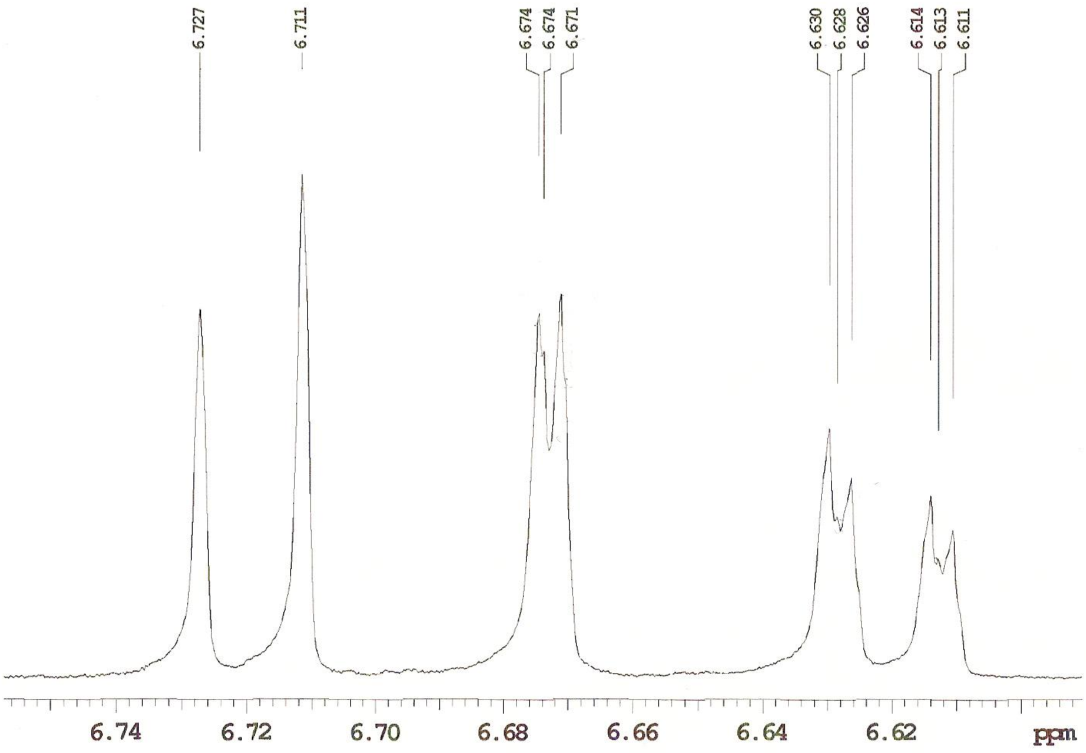

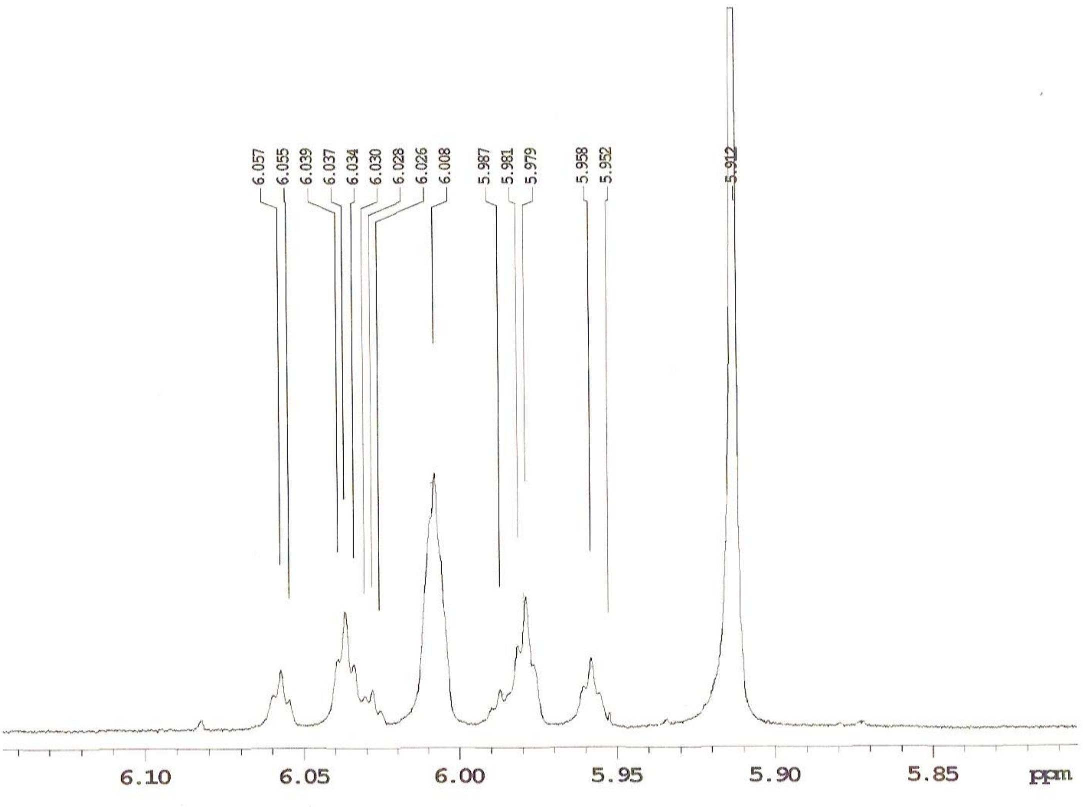

Figuras 100 e 101: expansões do espectro de RMN de ^{1}H (δ, 500 MHz, $CDCl_3$) de Pmt-4

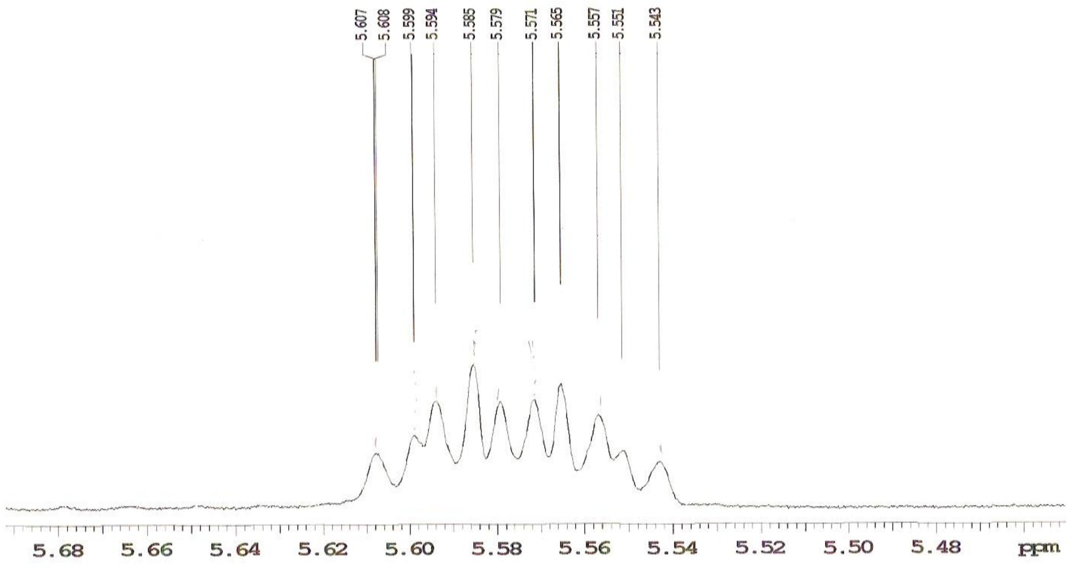

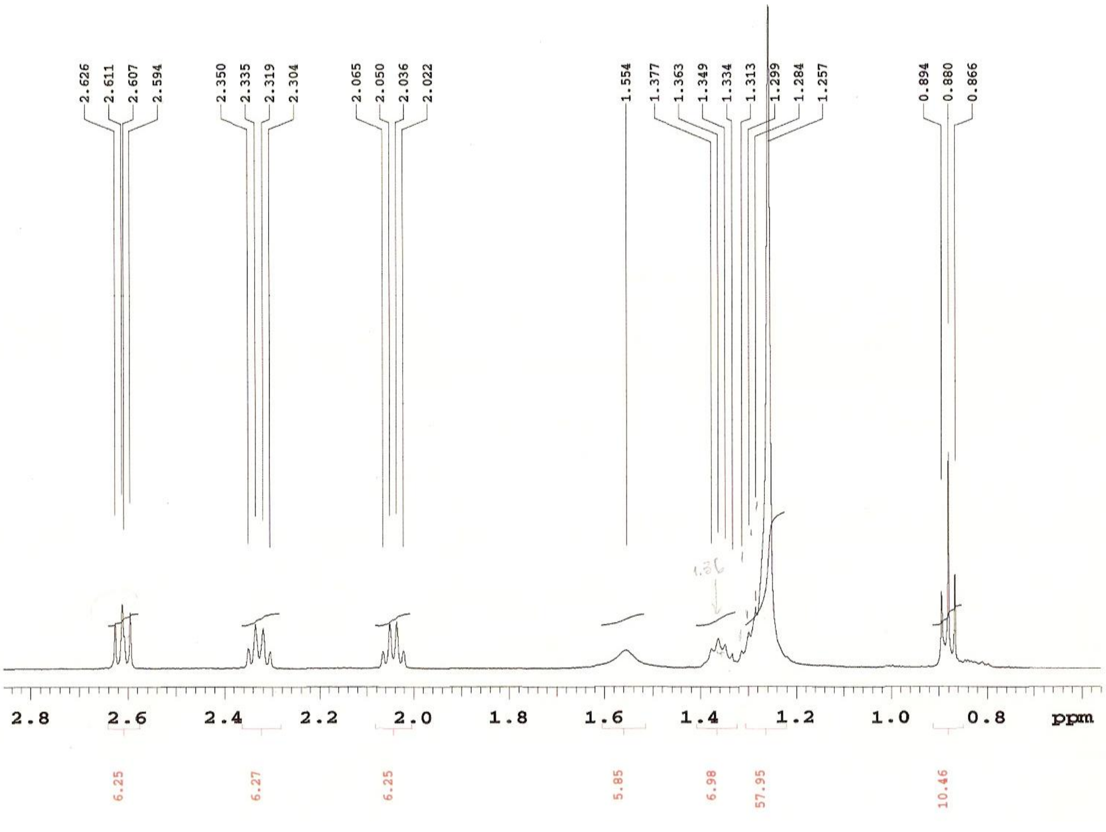

Figuras 102 e 103: expansões do espectro de RMN de ^{1}H (δ, 500 MHz, $CDCl_3$) de Pmt-4

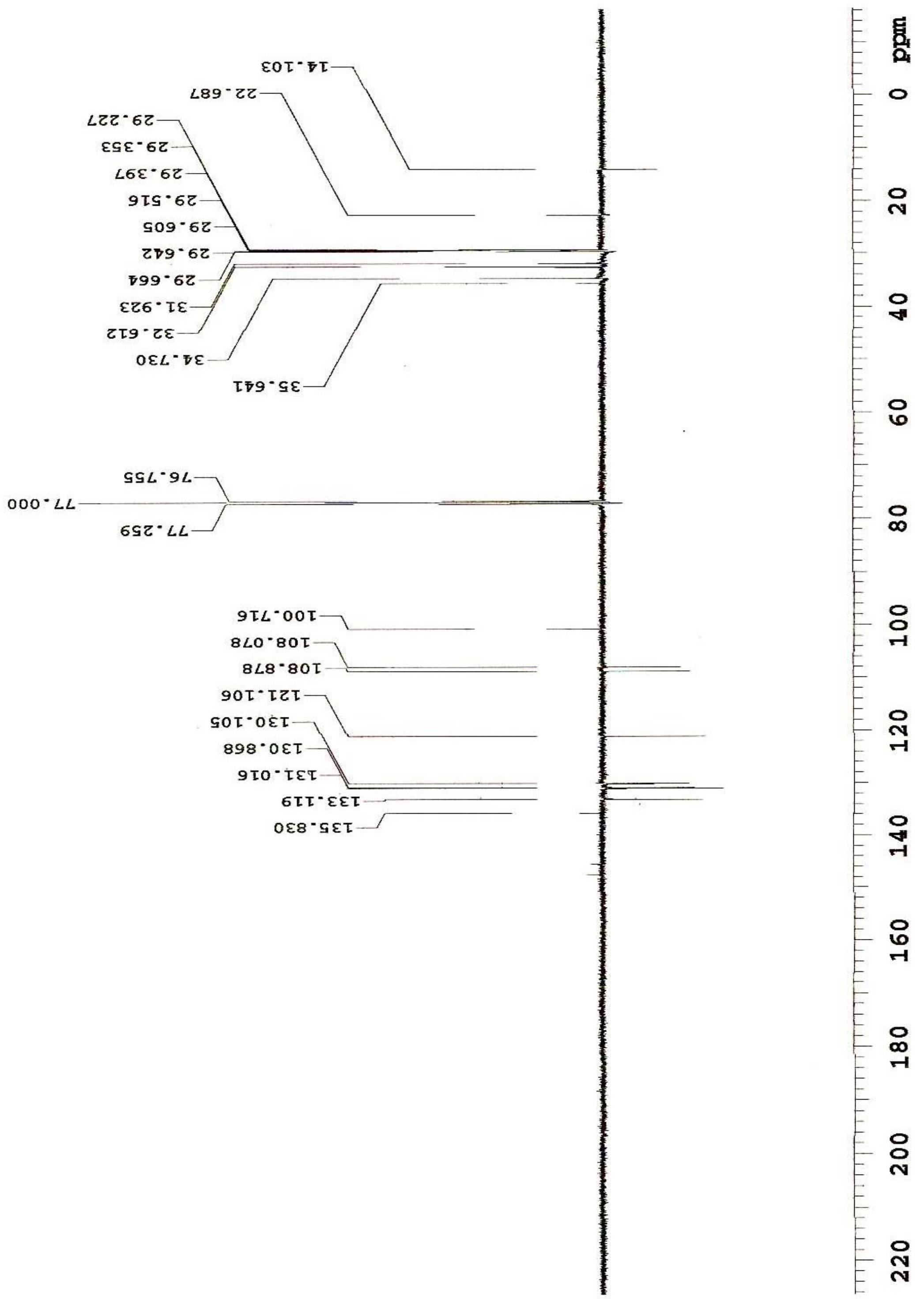

Figura 104: espectro de RMN de ^{13}C (δ, 125 MHz, $CDCl_3$) de Pmt-4

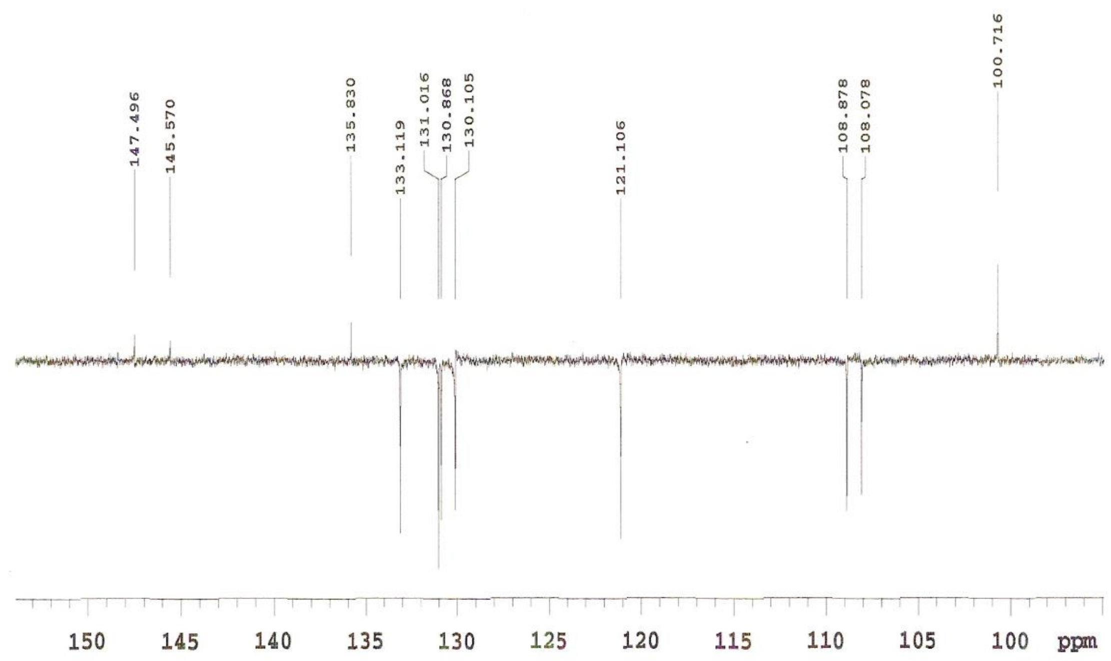

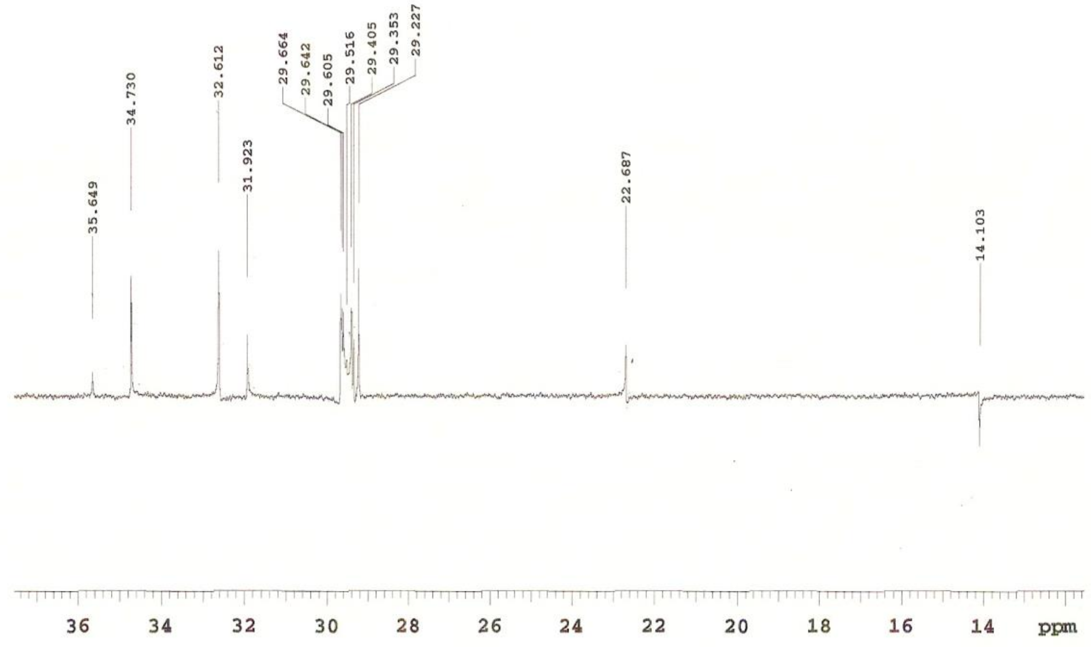

Figuras 105 e 106: expansões do espectro de RMN de ^{13}C (δ, 125 MHz, $CDCl_3$) de Pmt-4

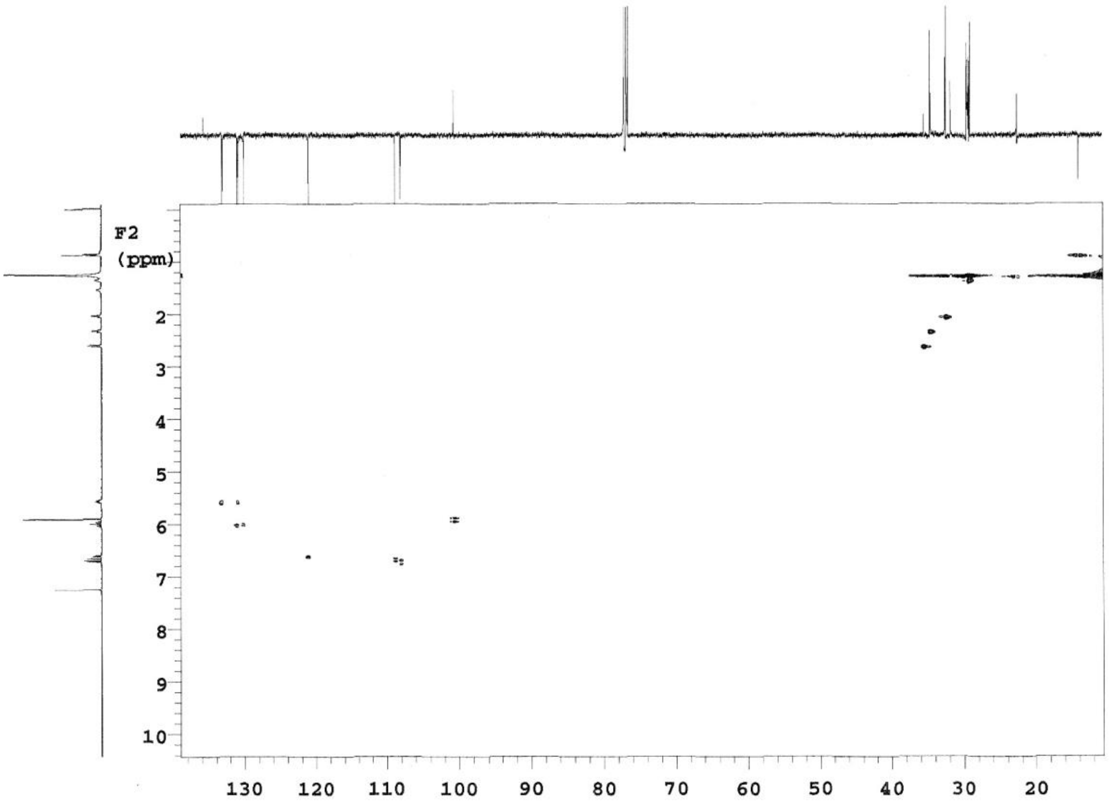

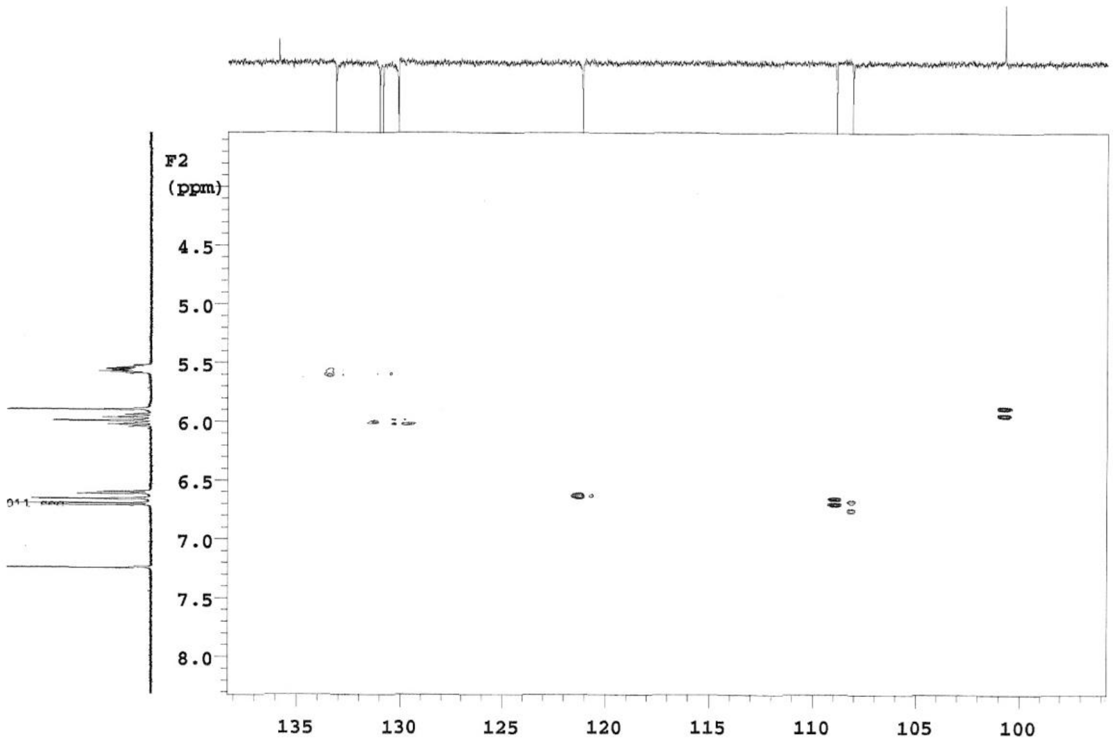

Figuras 107 e 108: espectro de correlação heteronuclear HMQC – ^{1}H x ^{13}C de Pmt-4

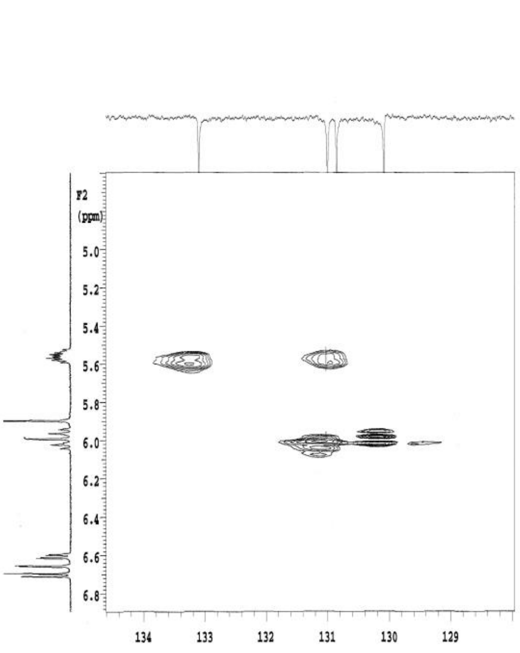

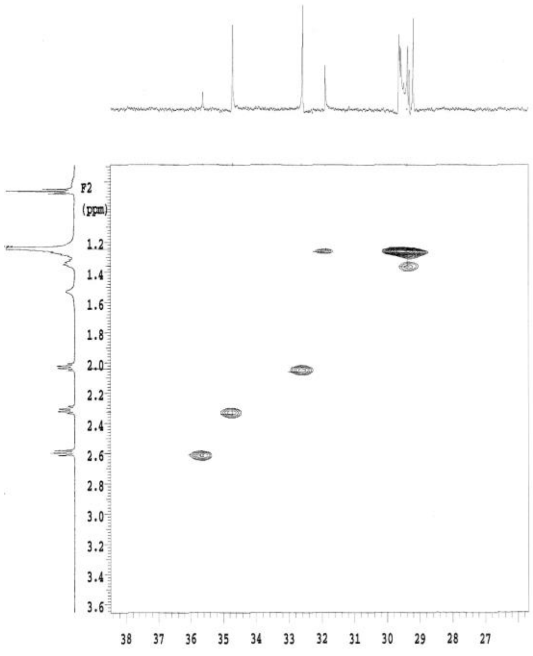

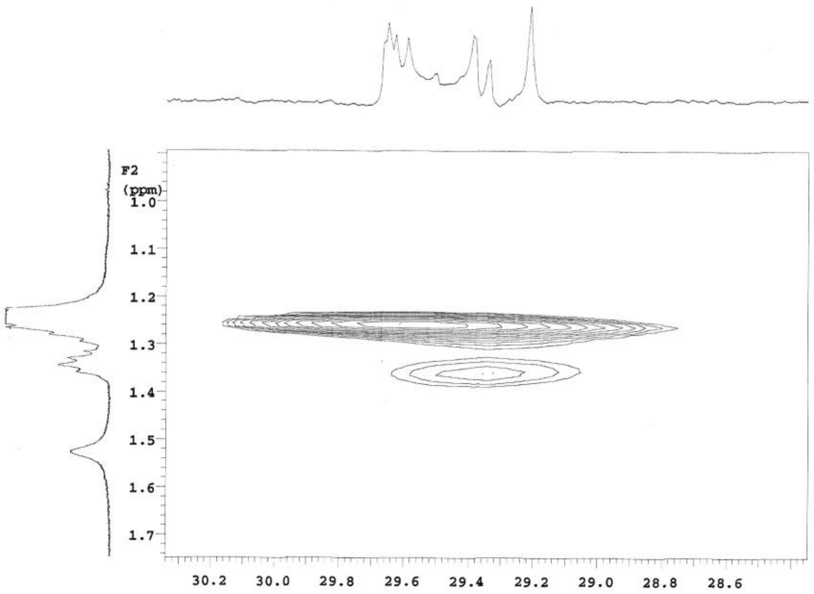

Figuras 109, 110 e 111: expansões do espectro de correlação heteronuclear HMQC – ^{1}H x ^{13}C de Pmt-4

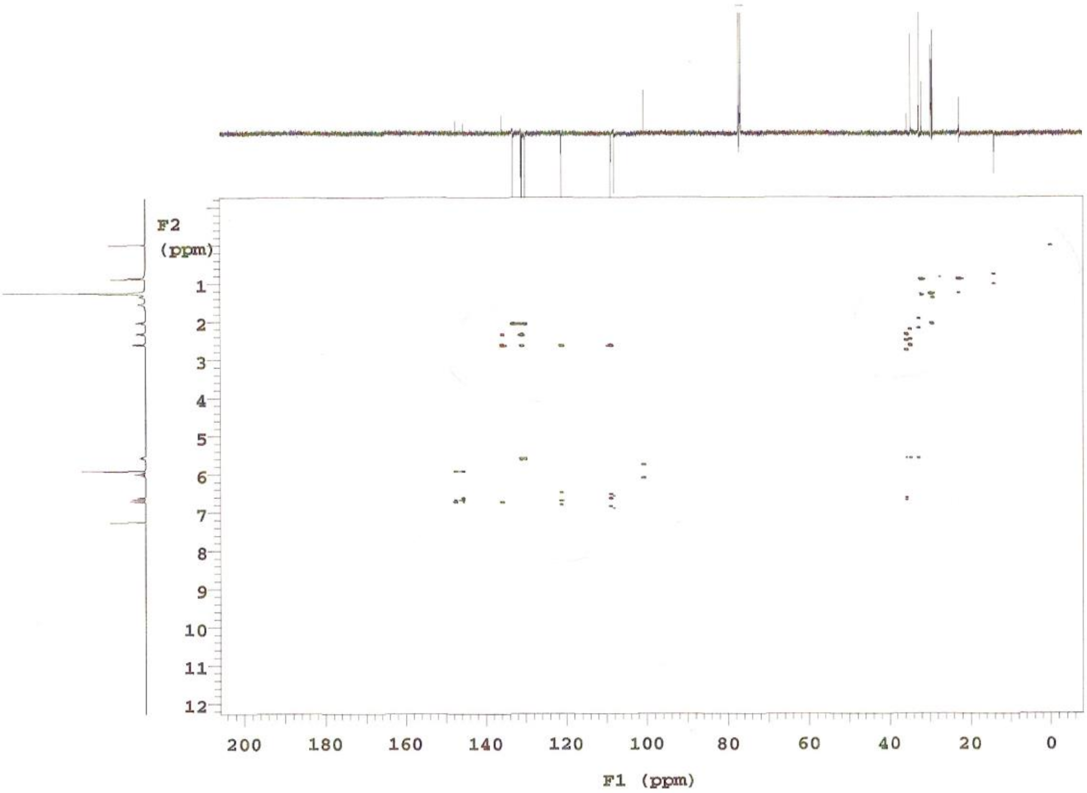

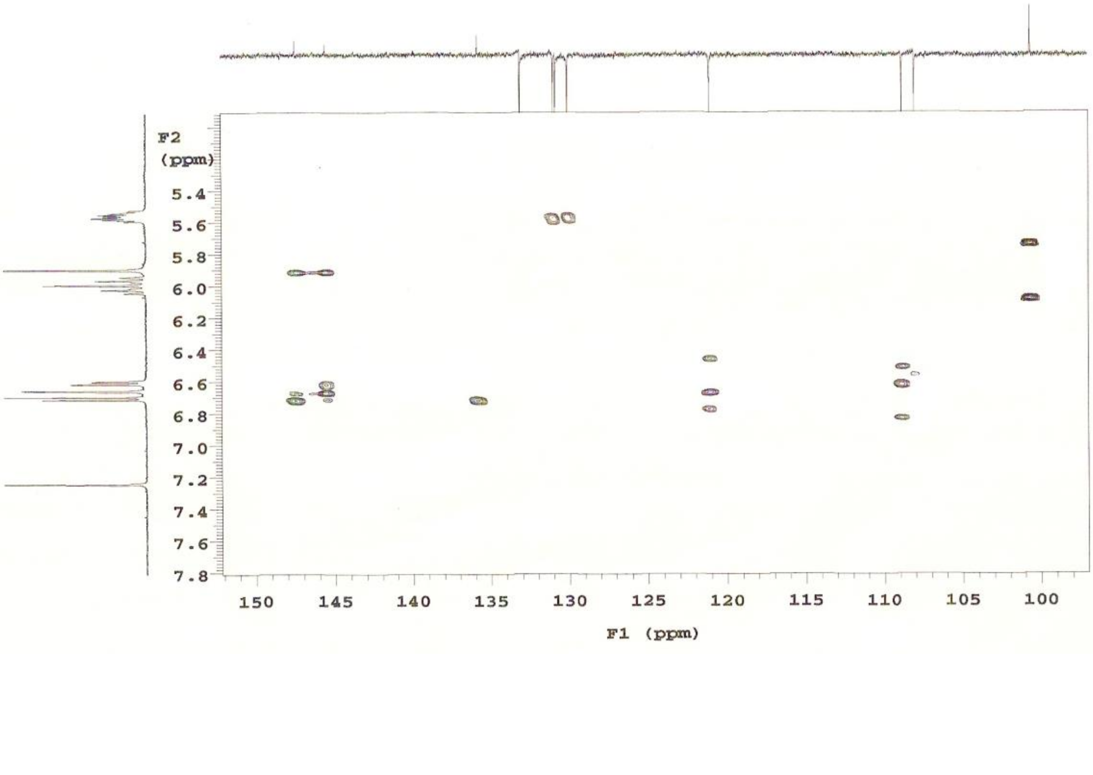

Figuras 112 e 113: expansões do espectro de correlação heteronuclear HMBC – ^{1}H x ^{13}C de Pmt-4

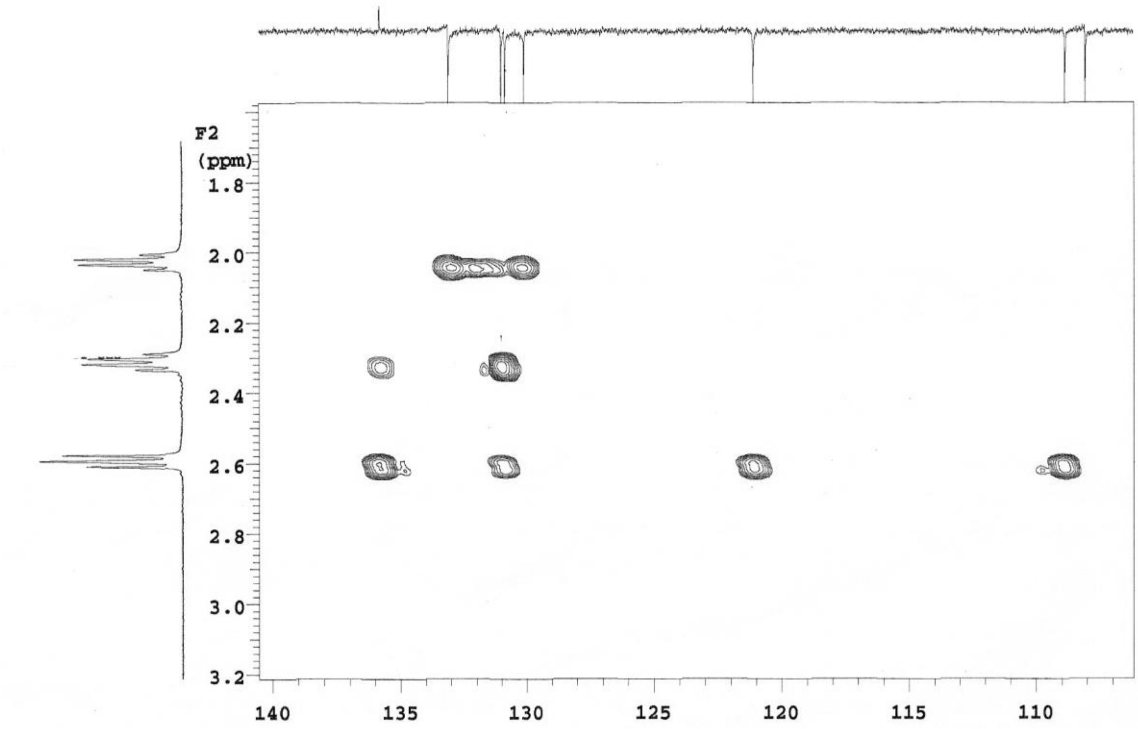

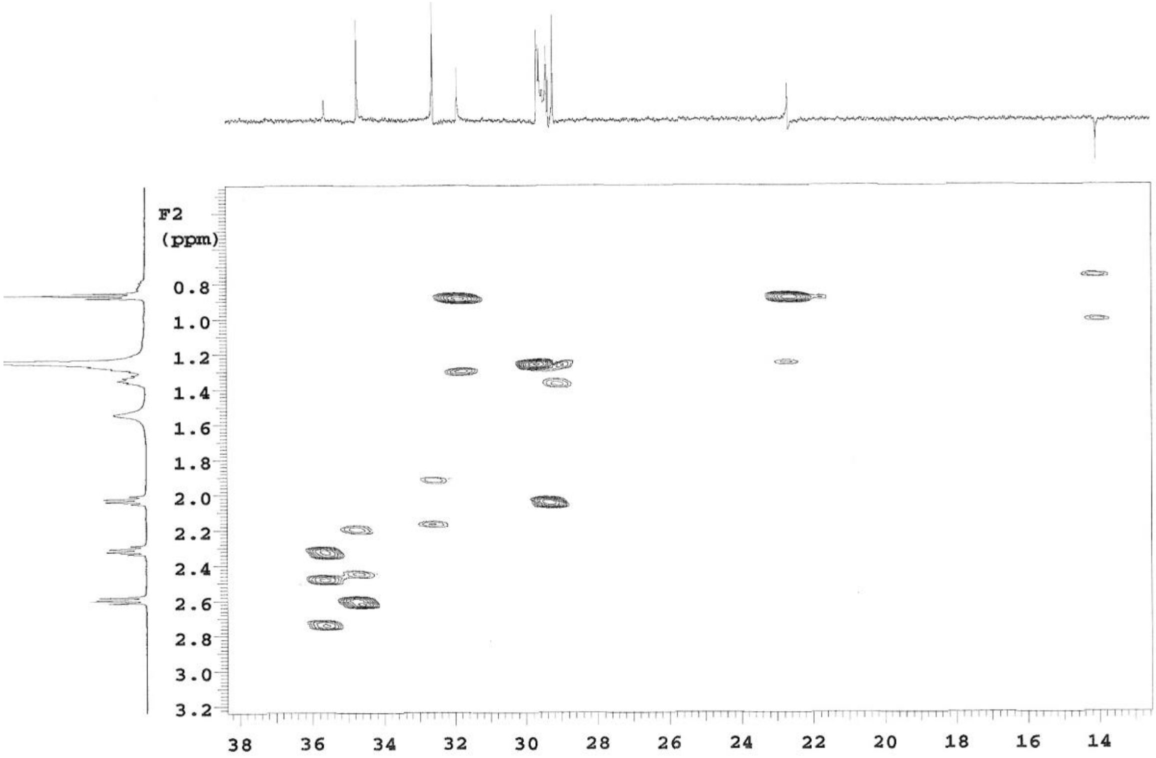

Figuras 114 e 115: expansões do espectro de correlação heteronuclear HMBC – ^{1}H x ^{13}C de Pmt-4

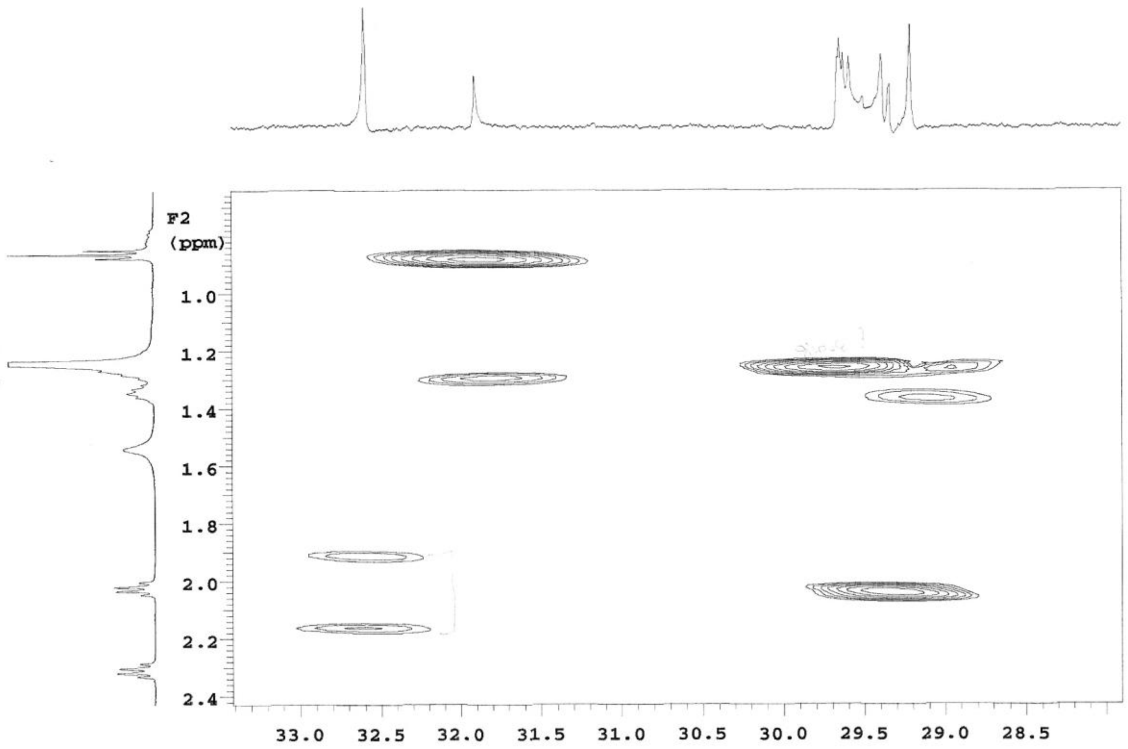

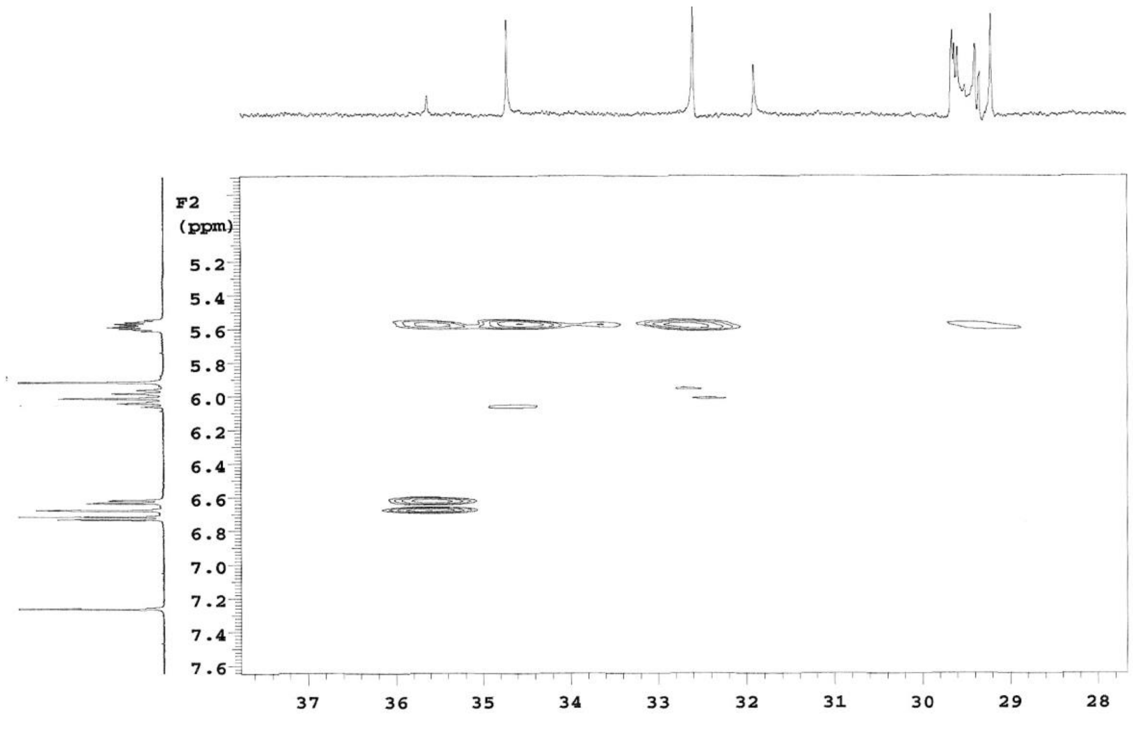

Figuras 116 e 117: expansões do espectro de correlação heteronuclear HMBC – ^{1}H x ^{13}C de Pmt-4

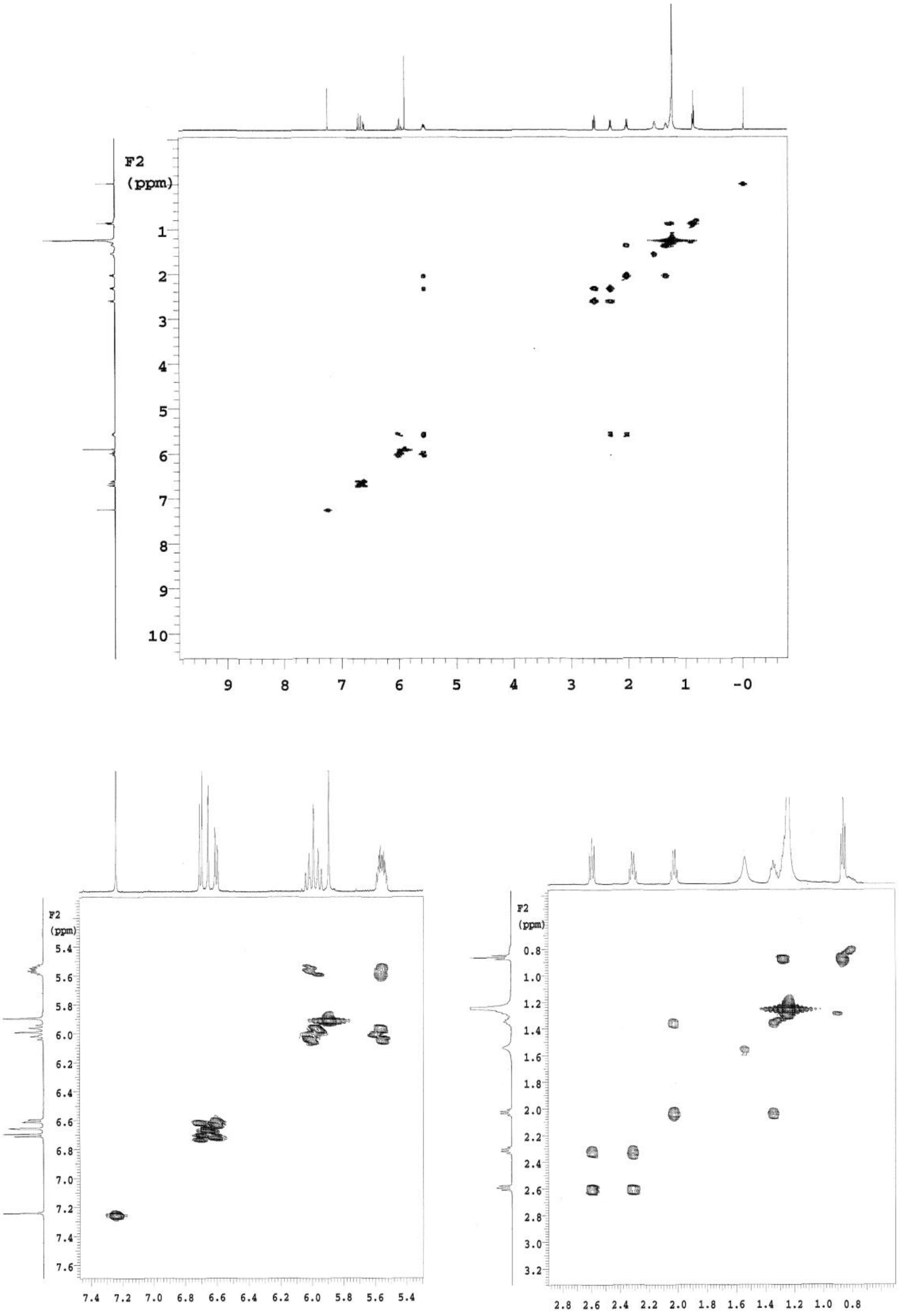

Figuras 118, 119 e 120: expansões do espectro de correlação homonuclear COSY – $^{1}H \times {}^{1}H$ de Pmt-4

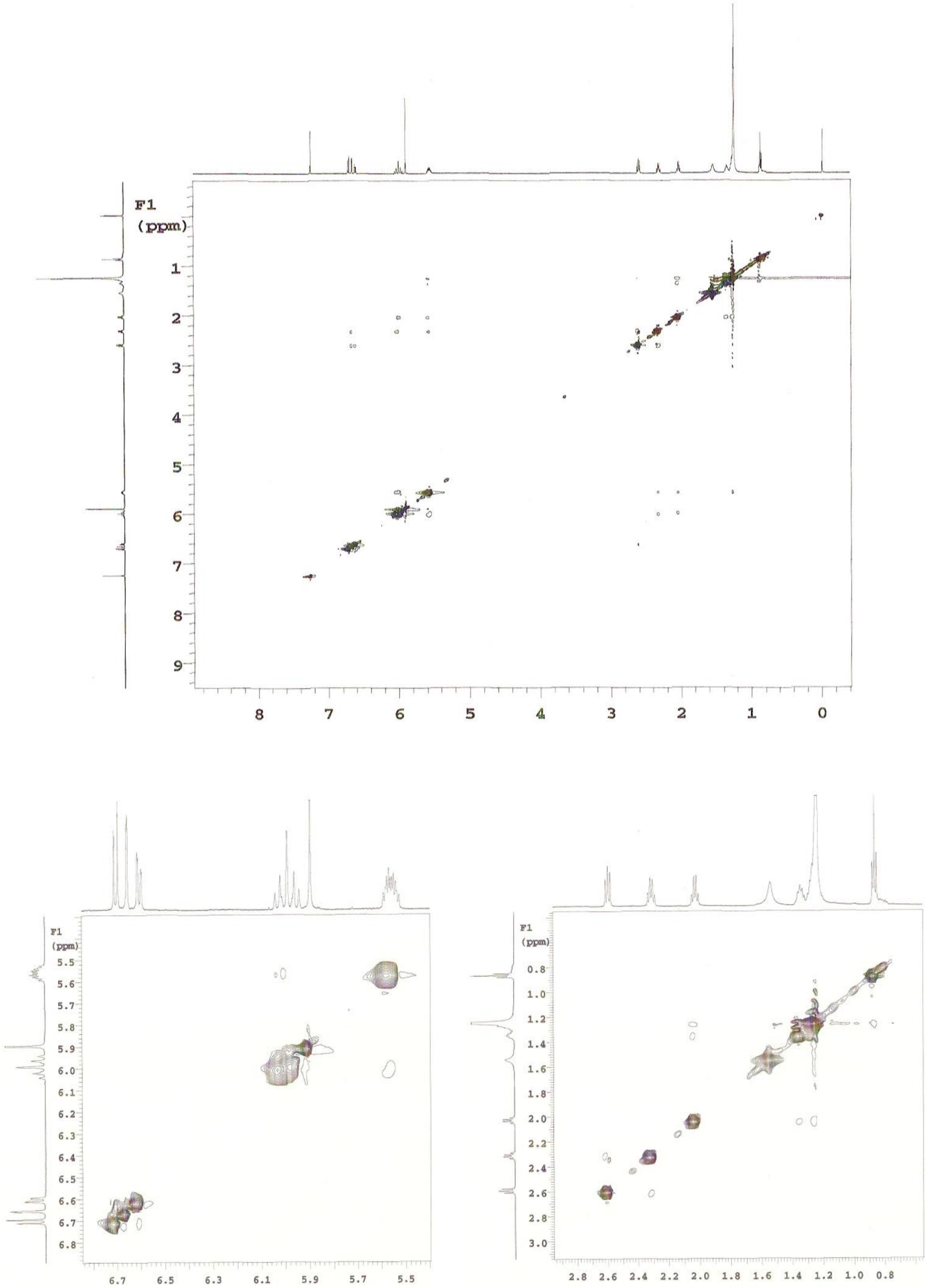

Figuras 121, 122 e 123: expansões do espectro de correlação homonuclear NOESY – ^{1}H x ^{1}H de Pmt-4

5

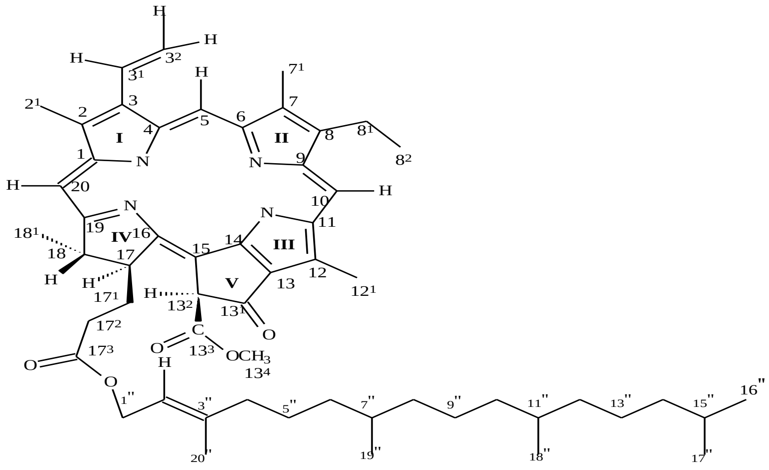

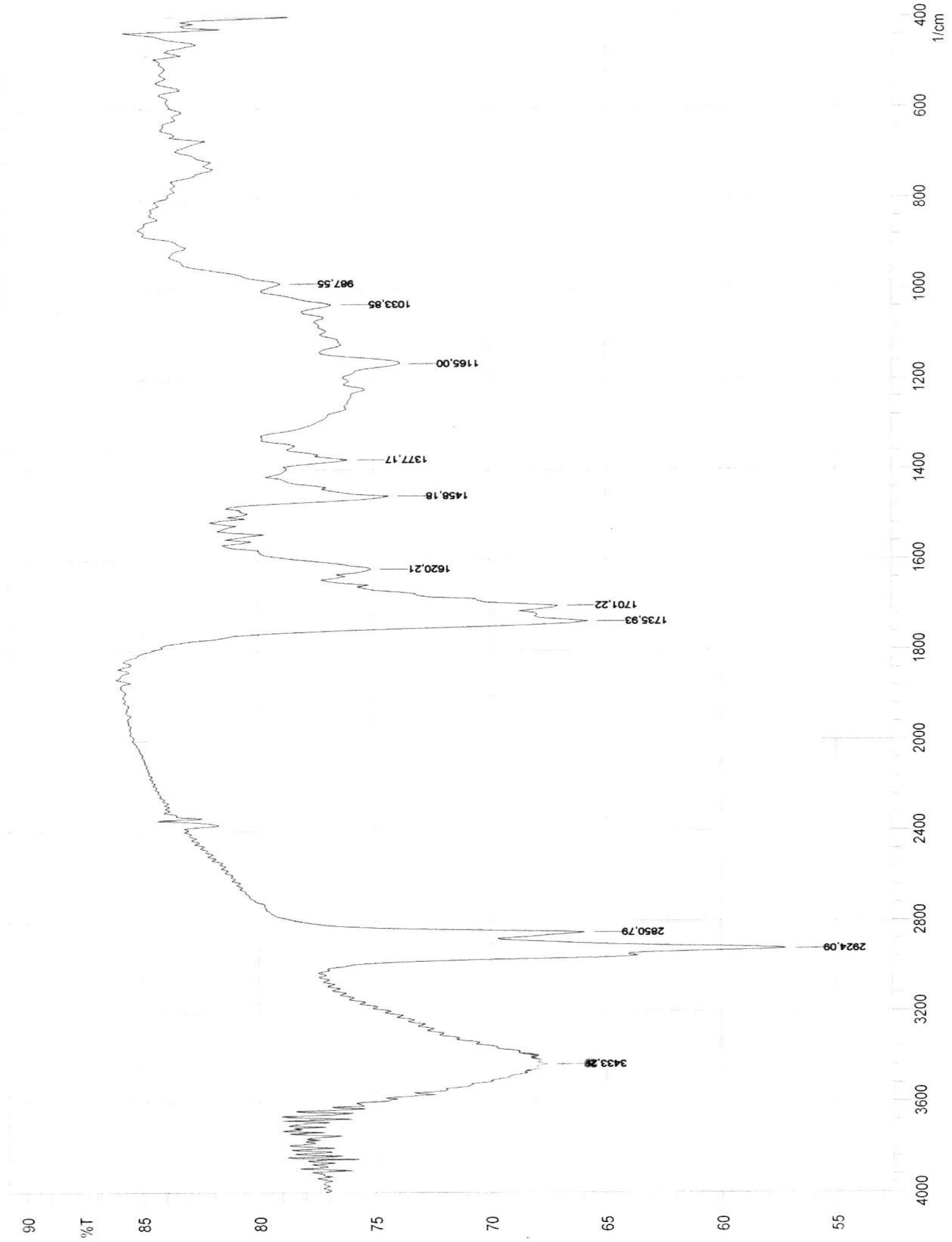

Figura 126: espectro no Infravermelho (λ_{max}, KBr, cm^{-1}) de Pmt-5

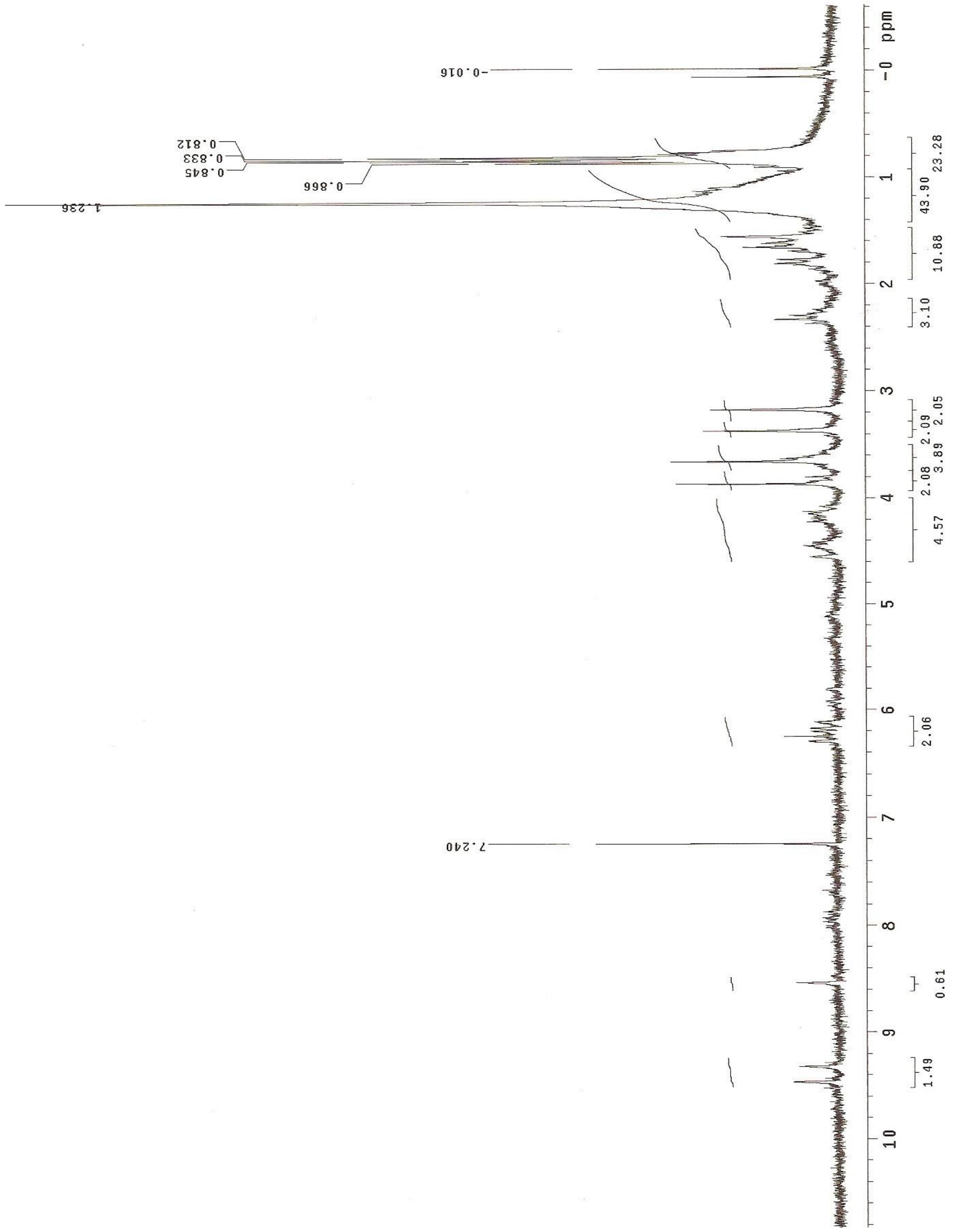

Figura 127: espectro de RMN de ^{1}H (δ, 200 MHz, $CDCl_3$) de Pmt-5

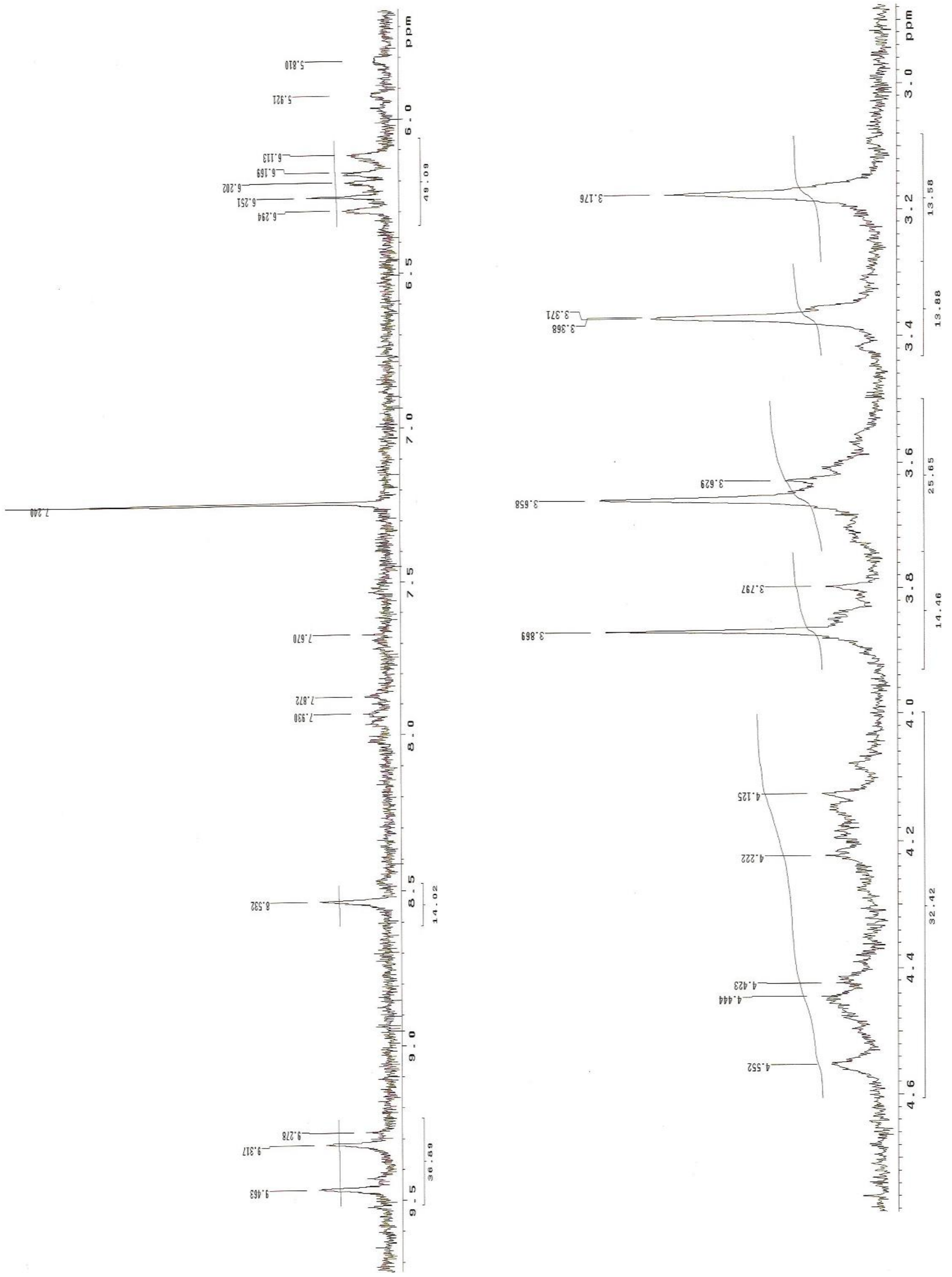

Figura 128 e 129: expansões do espectro de RMN de ¹H (δ, 200 MHz, $CDCl_3$) de Pmt-5

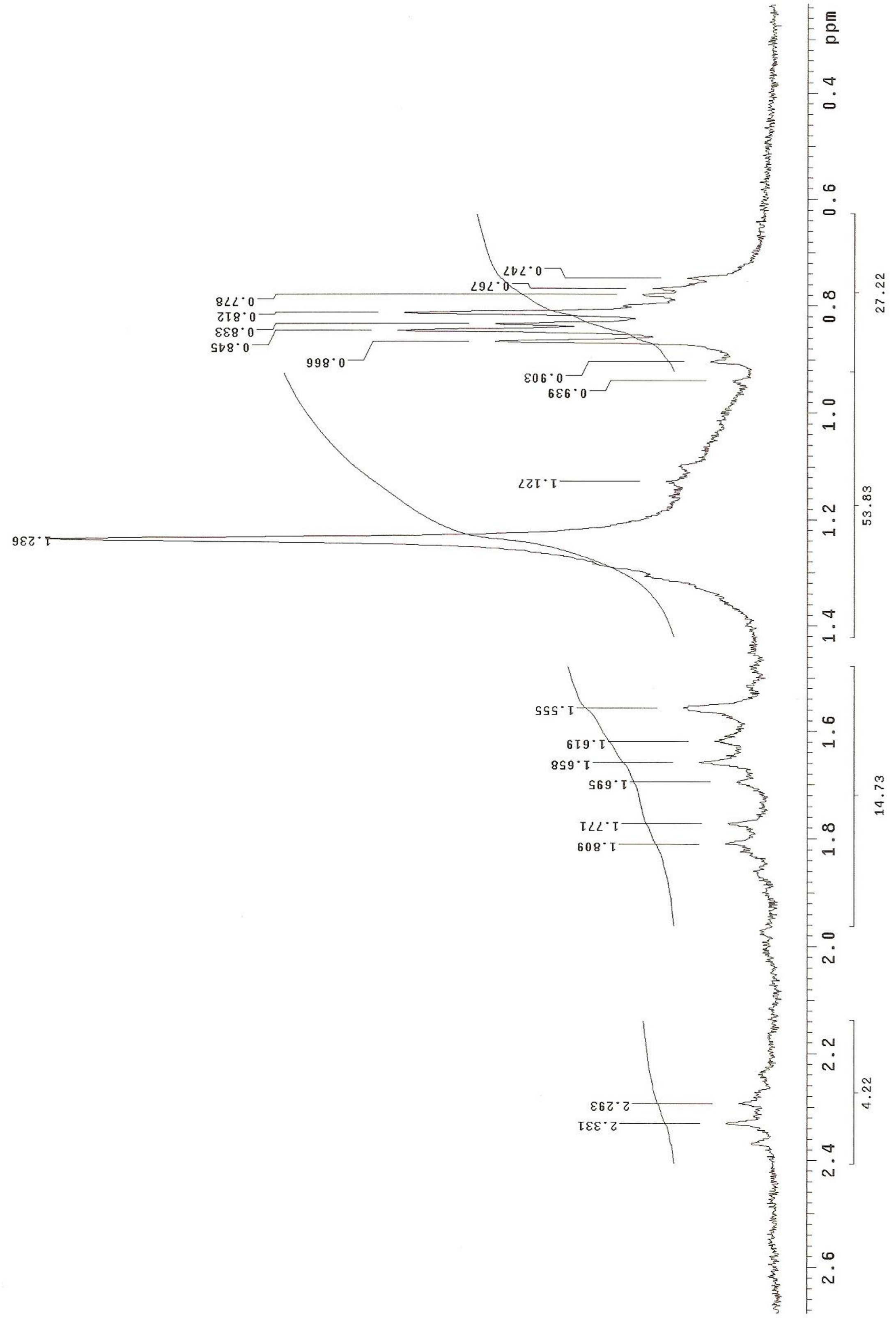

Figura 130: expansão do espectro de RMN de ^{1}H (δ, 200 MHz, $CDCl_3$) de Pmt-5

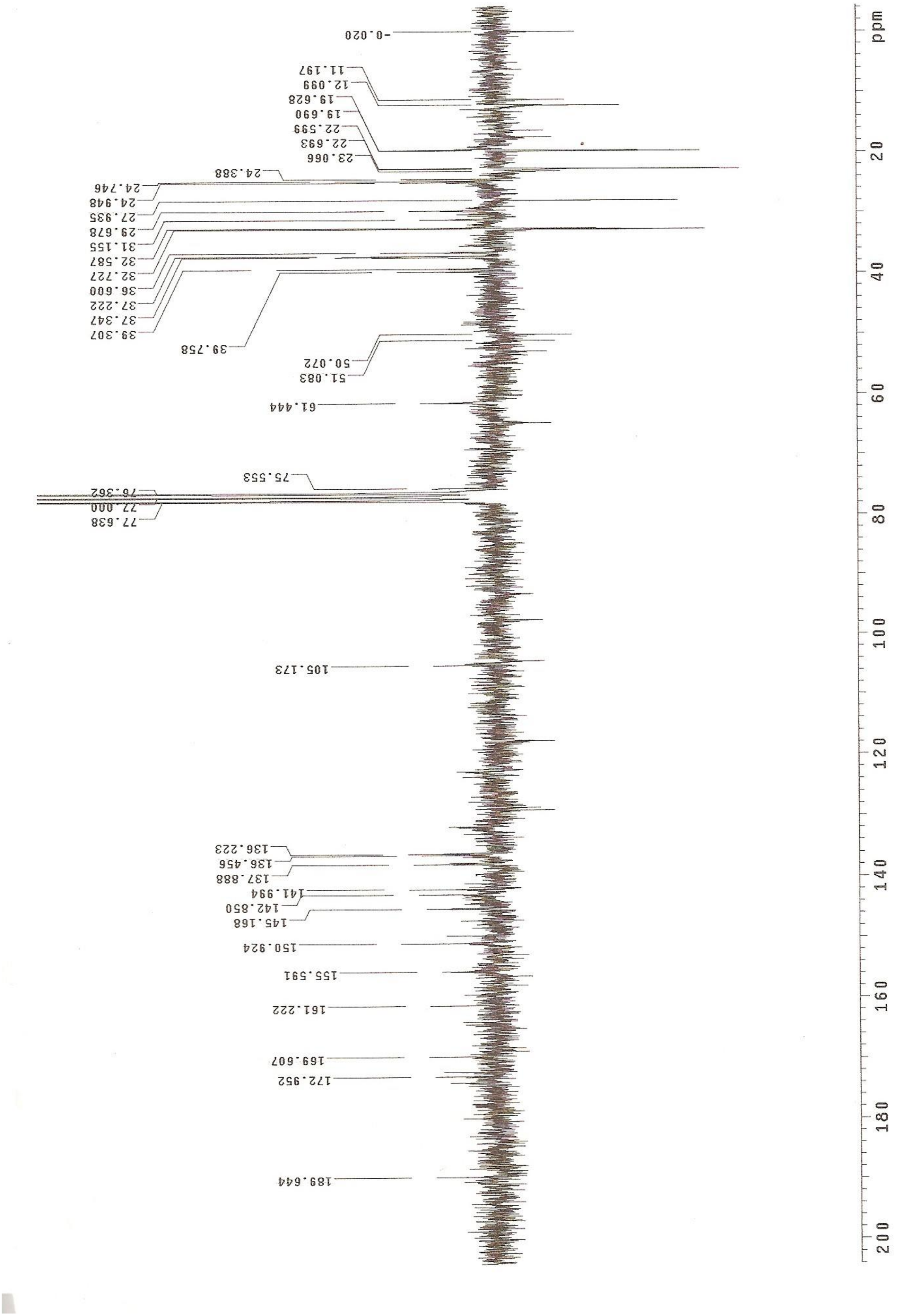

Figura 131: espectro de RMN de ^{13}C (δ, 50 MHz, $CDCl_3$) de Pmt-5

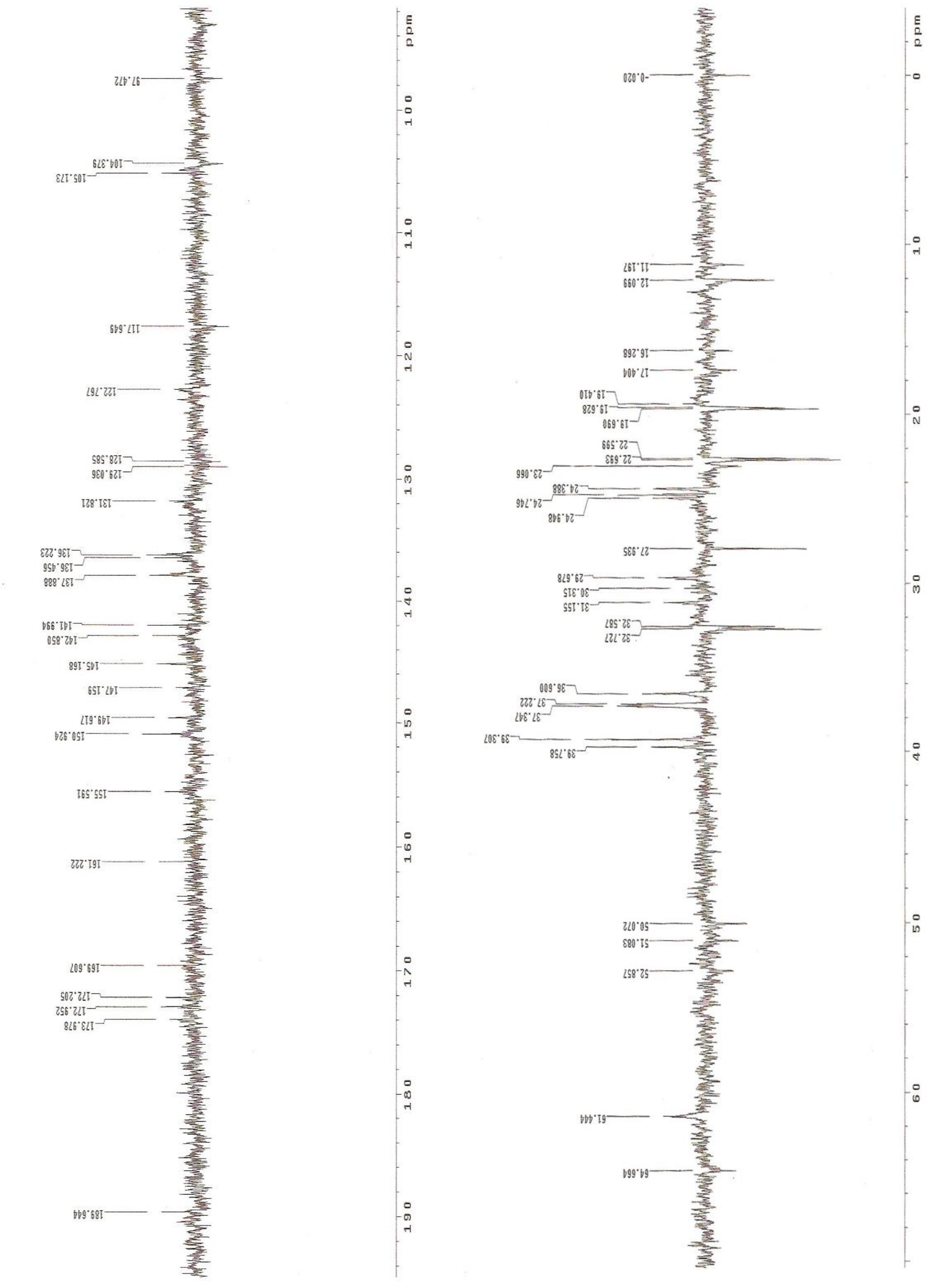

Figura 132 e 133: expansões do espectro de RMN de ^{13}C (δ, 50 MHz, $CDCl_3$) de Pmt-5

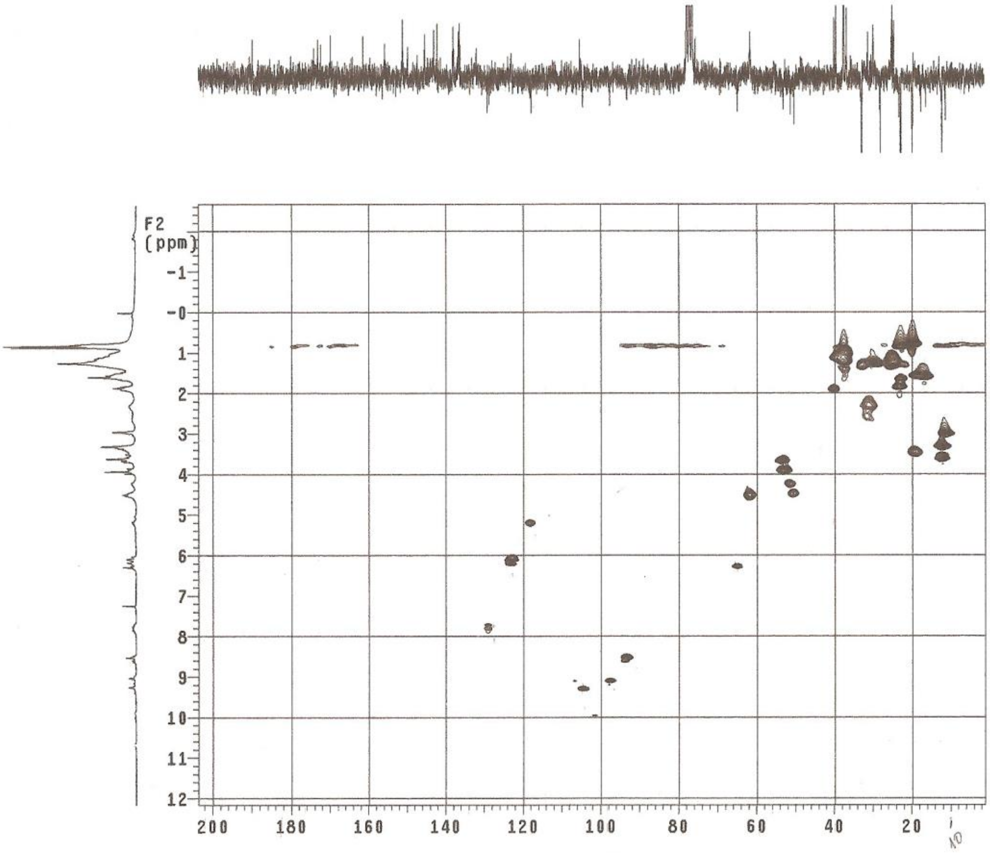

Figura 134: espectro de correlação heteronuclear HMQC – ^{1}H x ^{13}C de Pmt-5

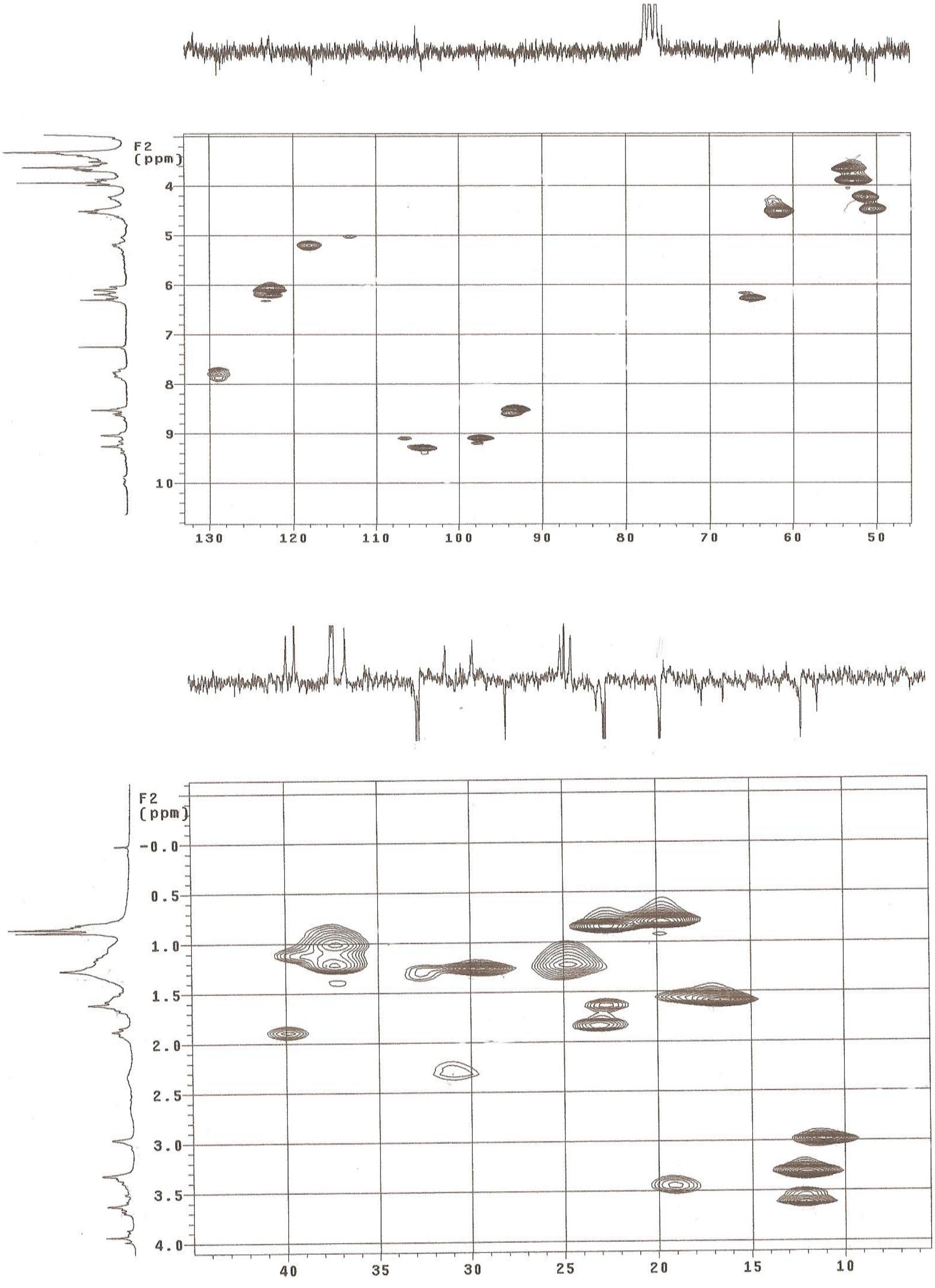

Figura 135 e 136: expansões do espectro de correlação heteronuclear HMQC – ^{1}H x ^{13}C de Pmt-5

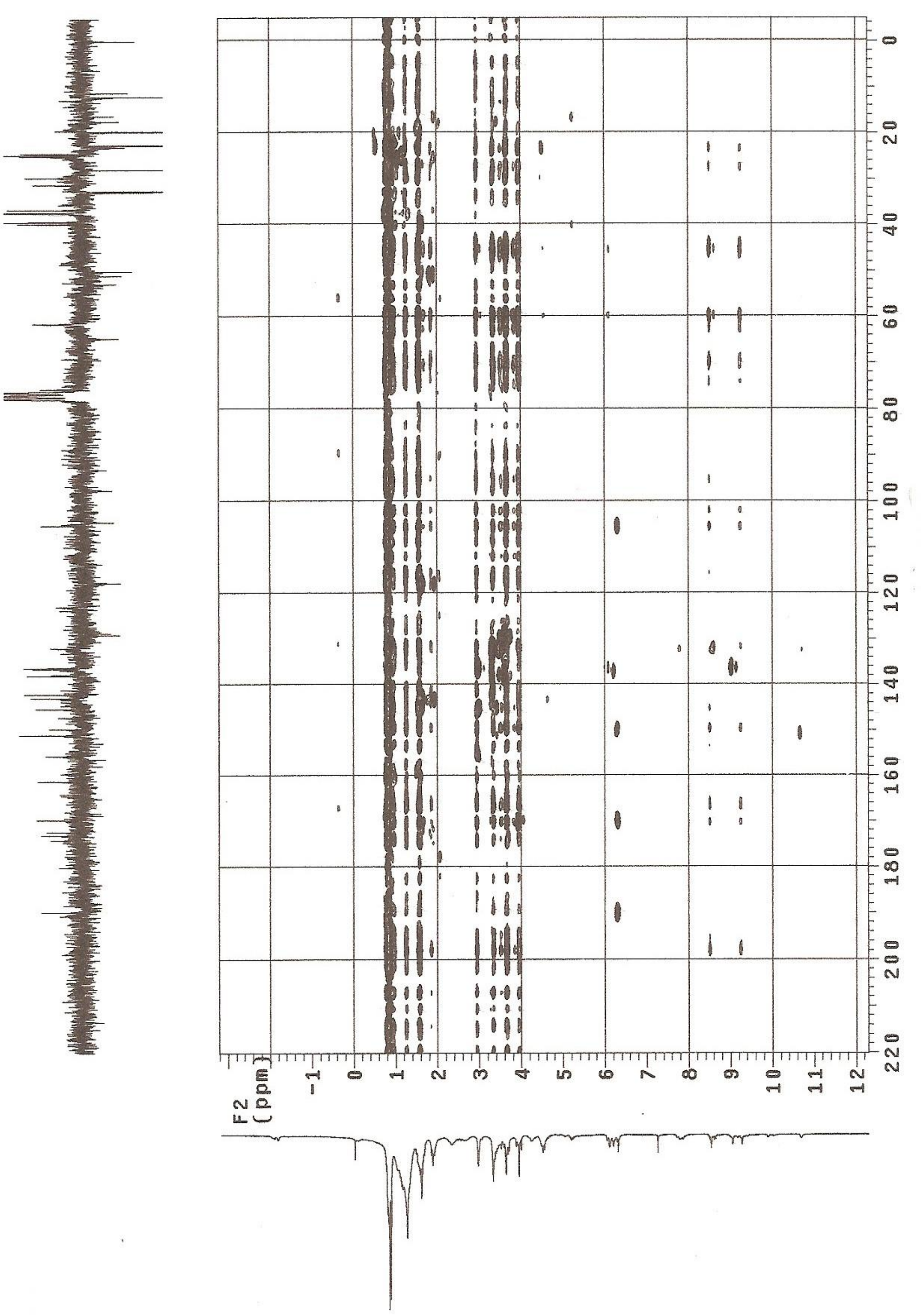

Figura 137: espectro de correlação heteronuclear HMBC – ^{1}H x ^{13}C de Pmt-5

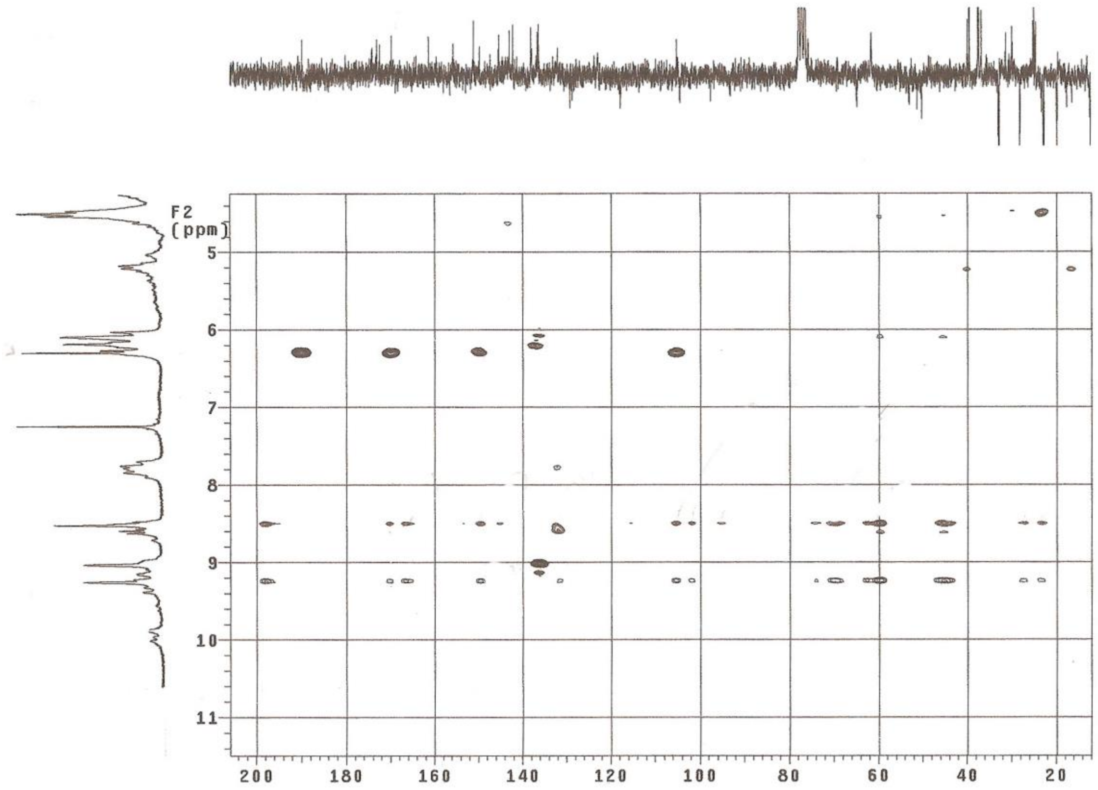

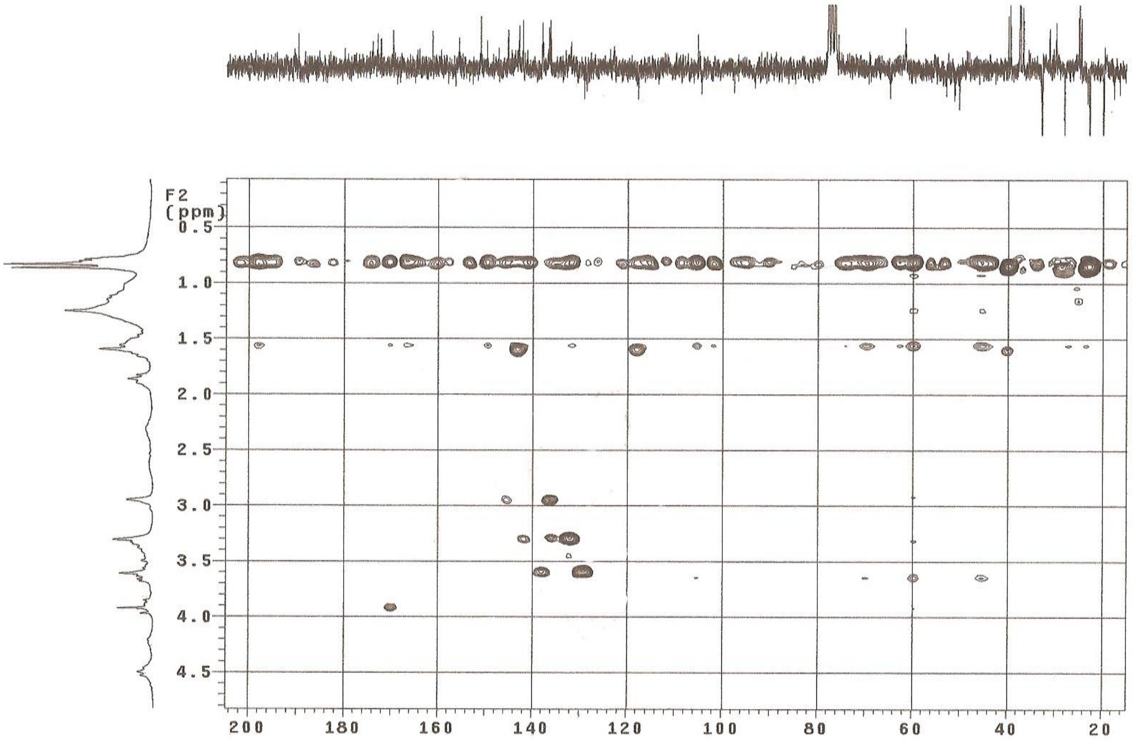

Figura 138 e 139: expansões do espectro de correlação heteronuclear HMBC – ^{1}H x ^{13}C de Pmt-5

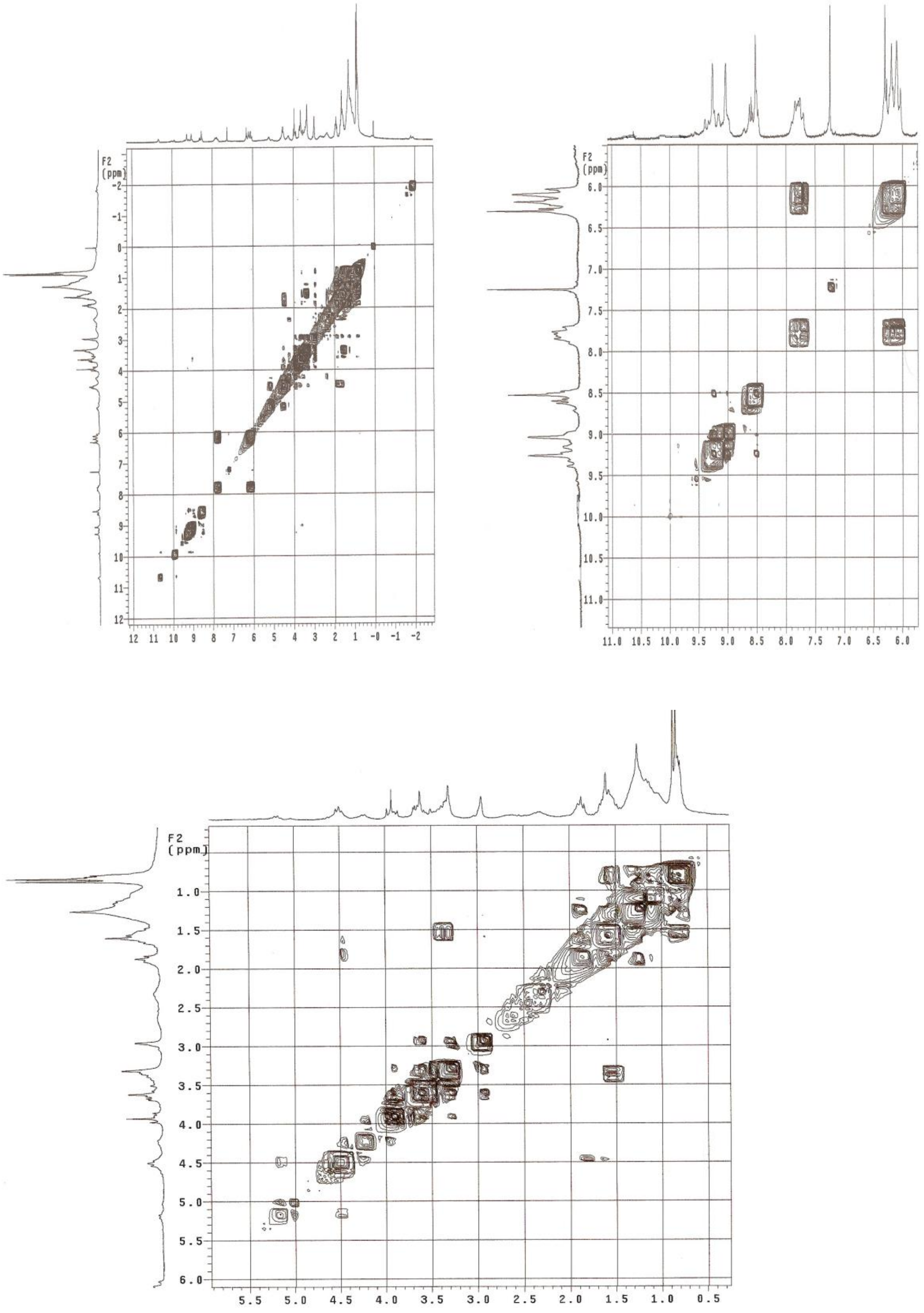

Figura 140, 141 e 142: expansões do espectro de correlação homonuclear COSY – ^{1}H x ^{1}H de Pmt-5

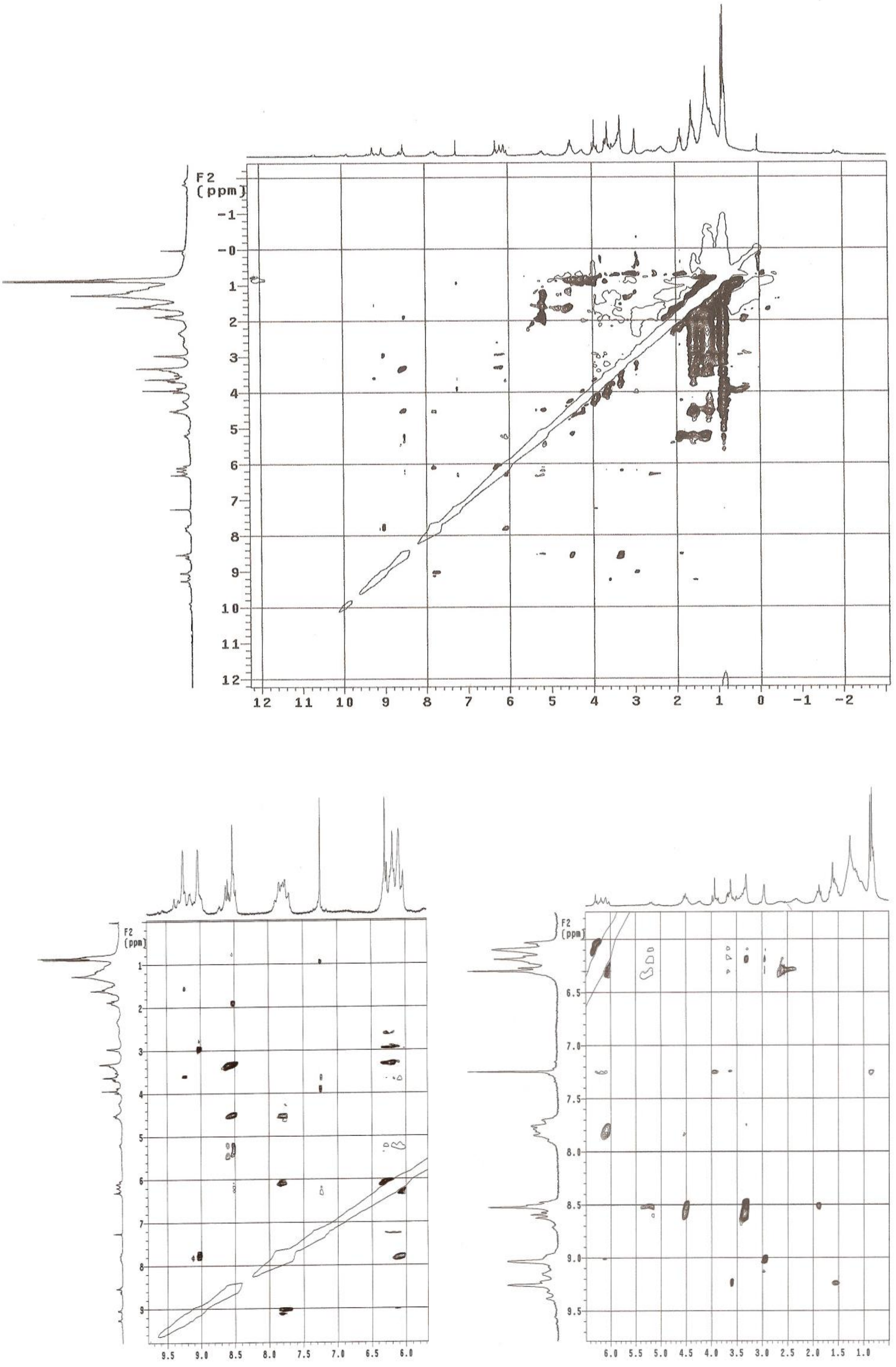

Figura 143, 144 e 145: expansões do espectro de correlação homonuclear NOESY – ^{1}H x ^{1}H de Pmt-5
